# Efficient Assembly of Functional RNA by *in Situ* Phosphate Activation and Loop-Closing Ligation

**DOI:** 10.1021/jacs.5c10478

**Published:** 2025-10-20

**Authors:** Jian Zhang, Aleksandar Radakovic, Filip Bošković, Harry R. M. Aitken, Long-Fei Wu, Jack W. Szostak

**Affiliations:** † Howard Hughes Medical Institute, Department of Chemistry, 2462 The University of Chicago, Chicago, Illinois 60637, United States; ‡ Department of Ecology and Evolution, The University of Chicago, Chicago, Illinois 60637, United States; § Howard Hughes Medical Institute, Massachusetts General Hospital, Boston, Massachusetts 02114, United States; ∥ Department of Chemistry and Chemical Biology, 1812 Harvard University, 12 Oxford Street, Cambridge, Massachusetts 02138, United States; ⊥ Frontiers Science Center for Transformative Molecules, State Key Laboratory of Synergistic Chem-Bio Synthesis, School of Chemistry and Chemical Engineering, Zhangjiang Institute for Advanced Study, Shanghai Jiao Tong University, Shanghai, 200240, China

## Abstract

How ribozymes might have emerged prior to the evolution of an efficient RNA replicase remains an open question. A potential solution involves the nonenzymatic ligation of RNA oligomers that are short enough to have been replicated by nonenzymatic chemistry. However, the high concentrations of metal ions that facilitate ligation are incompatible with primitive protocells. In addition, the mode of oligoribonucleotide 5′-phosphate activation is unclear, and in an aqueous environment the competing hydrolysis of such activated intermediates limits ligation efficiency. In this study, we have investigated the template-independent assembly of structured RNAs via loop-closing ligation using a potentially prebiotic *in situ* phosphate activation approach. We demonstrate that the 5′-phosphate of oligonucleotides can be activated through an *N*-methylimidazole-intercepted phospho-Passerini reaction, enabling subsequent loop-closing ligation to efficiently form hairpin structures. Remarkably, this reaction proceeds in the absence of divalent metal ions and remains effective even at low millimolar concentrations of the activating reagent. We identify a novel pathway leading to a phosphorimidazolium species, mediated by an *N*-imidoyl-*N*′-methylimidazolium intermediate. Furthermore, we show that two successive *in situ* activation/loop-closing ligations enable the assembly of a functional RNA Flexizyme with a near-quantitative yield. Our findings provide insight into potential mechanisms for the prebiotic origin of functional RNAs and offer a viable strategy for the chemical synthesis of long RNA molecules.

## Graphical abstract

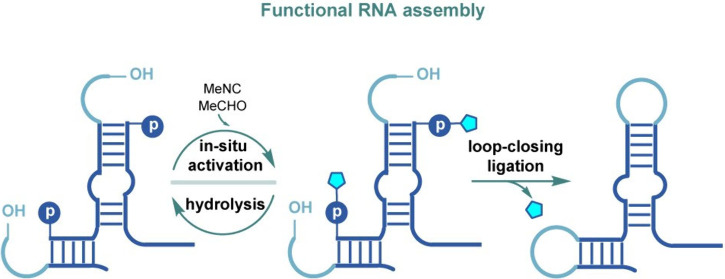

## Introduction

The RNA world hypothesis^1,2^ posits that RNA functioned as both the genetic material and the primary catalytic molecule in early self-replicating protocells. This dual functionality is reflected in modern biology, where RNA acts as the genetic material in RNA viruses and as catalysts in the form of ribozymes.^3,4^ One of the most compelling pieces of evidence supporting this hypothesis is the structural composition of the ribosome—specifically, the fact that the peptidyl transferase center is composed entirely of RNA.^5,6^ This feature is widely regarded as a molecular relic linking the RNA world to contemporary life.^7^ From an evolutionary perspective, RNA-based catalysis may have been progressively replaced by more efficient protein enzymes, leading to the decline of ribozymes and the eventual extinction of life forms that relied exclusively on RNA.^8^ Fortunately, *in vitro* selection facilitates the discovery of functional RNA structures^9,10^ including polymerase ribozymes,^11–13^ raising the possibility of reconstructing a self-replicating RNA world under laboratory conditions. However, a fundamental question remains unresolved: how did the first functional RNA molecules emerge in the absence of preexisting replicases?

The assembly of long, structured functional RNAs likely involved the ligation of short oligoribonucleotides, a process most efficiently achieved through bottom-up, convergent synthesis.^14,15^ However, the formation of phosphodiester bonds to link short unactivated oligonucleotides into longer oligomers is both thermodynamically and kinetically unfavorable in aqueous environment. To overcome this reaction barrier, high-energy precursors are necessary. In modern biology, this is accomplished through enzyme-mediated catalysis, where the chemical energy of phosphate anhydrides, such as ATP, drives phosphodiester bond formation.^16,17^ In contrast, nonenzymatic ligation typically relies on preactivated 5′-phosphorimidazolide oligonucleotides^18–22^ or oligonucleotides ending in a 2′,3′-cyclic phosphate.^23,24^ For imidazole-activated oligonucleotides, the high-energy P–N bond facilitates ligation because imidazole is a good leaving group. However, this high reactivity is accompanied by increased susceptibility to hydrolysis, limiting the ligation efficiency. To improve efficiency, high concentrations of divalent metal ions are often used to lower the ligation energy barrier. However, these ions also promote RNA degradation^25^ and can disrupt fatty acid–based protocell compartmentalization by inducing aggregation.^26^ Moreover, laboratory synthesis of phosphorimidazolides typically involves chemical activation followed by precipitation or high-performance liquid chromatography purification, making it a discontinuous and labor-intensive process of doubtful prebiotic plausibility.

Efficient RNA ligation requires the close proximity and suitable orientation of the 3′–OH and the activated 5′-phosphate, which is typically achieved through a template strand that aligns the two RNA ends.^18–20,23,24^ However, template strands could inhibit ribozyme folding by binding tightly to the ligated product. To address this limitation and enhance ligation efficiency, templates are often removed through purification steps,^11,12,18^ or alternative strategies are employed, such as designing optimized template sequences and/or substituting 3′-hydroxyl groups with more nucleophilic amino groups.^19,20^ Recently, it was found that preactivated short duplexes with single-stranded overhangs could undergo loop-closing ligation via cross-strand reactions, directly forming RNA hairpin structures (**II** to **III**, Figure 1A).^21,22^ This process exploits the proximity of the two strands on the same end of a duplex.^27^ High-throughput screening identified overhang sequences (such as UNNG, CNNG, and GNNA) that efficiently promote loop-closing ligation, and these sequences are frequently found in extant biological tetraloops.^22^ Loop-closing ligation reduces the problem of inhibition by the templates used for splinted ligation. However, RNA assembly involving the closure of multiple loops remains suboptimal due to intrinsic activity limitations and competing hydrolysis.^22^

**Figure 1:**
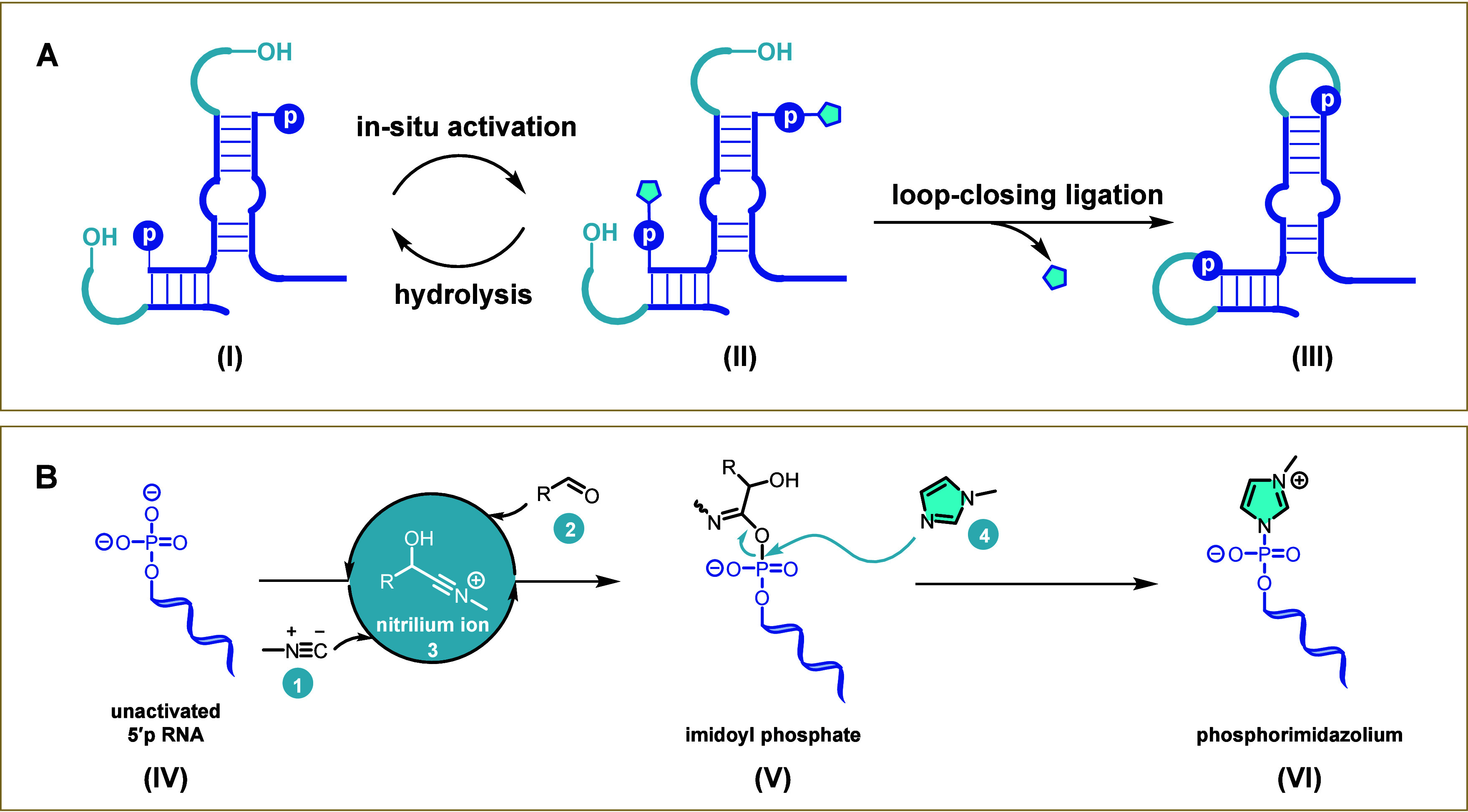
Functional RNA assembly driven by *in situ* phosphate activation and loop-closing ligation. (A) Schematic representation of functional RNA assembly. The 3′-end hydroxyl nucleophile is denoted as OH, the 5′-phosphate group as p, and the imidazole leaving group of the activated 5′-phosphate as a pentagon. (B) Proposed mechanism of *in situ* phosphate activation via a Passerini-type reaction.

We hypothesized that *in situ* phosphate activation, followed by loop-closing ligation chemistry, could address both the prebiotic plausibility of RNA assembly and the inefficiency resulting from hydrolysis (Figure 1A). Methyl isocyanide (MeNC, **1** in Figure 1B) has been proposed as a potentially prebiotic phosphate-activating agent, capable of activating phosphate either through acid catalysis^28^ or a Passerini-type reaction involving an aldehyde (**2**, Figure 1B).^29–31^ We envisioned that dynamically self-assembled short RNA structures (**I**) could undergo activation via an imidazole-intercepted Passerini reaction (Figure 1B), yielding phosphorimidazole-activated oligonucleotides (**II**). These activated intermediates could then undergo loop-closing ligation, generating full-length functional RNA products (**III**). In this study, we have explored conditions compatible with *in situ* phosphate activation and loop-closing ligation, employing methyl isocyanide in combination with a coactivating aldehyde and an *N*-methylimidazole catalyst (Figure 1B). Under optimized conditions, we achieved near-quantitative assembly of a Flexizyme through two concurrent loop-closing ligations. Nuclear magnetic resonance (NMR) spectroscopy identified a *N*-imidoyl-*N*′-methylimidazolium (IMI) species that activates phosphate, forming an *N*-methyl-phosphorimidazolium intermediate which subsequently undergoes metal independent loop-closing ligation. Our findings provide mechanistic insight into the prebiotic self-assembly of structured, functional RNAs and suggest potential applications in chemical RNA synthesis.

## Results

### Essential Components for *in Situ* Phosphate Activation and Loop-Closing Ligation

To model the loop-closing ligation reaction, we used a 6 base pair duplex as the starting material (Figure 2A). The duplex consisted of a phosphate acceptor strand (P1) with a 3′-UUCG overhang and a phosphate donor strand (L1).^22^ To enable quantitative analysis, the phosphate acceptor strand (P1) was labeled at its 5′-end with a BODIPY fluorophore. We hypothesized that the transient nitrilium species (**3**, Figure 1B), formed from MeNC and aldehyde, could be attacked by the 5′-phosphate of the RNA (**IV**) to generate a 5′-imidoylphosphate RNA (**V**), which would subsequently be intercepted by *N*-methylimidazole (**4**, Figure 1B), yielding the active 5′-phosphorimidazolium (**VI**) for ligation.^21,22,29–31^ For initial reaction conditions, we used 1 μM duplex RNA, 200 mM MeNC, 200 mM acetaldehyde, 200 mM *N*-methylimidazole, and 200 mM HEPES (4-(2-hydroxyethyl)-1-piperazineethanesulfonic acid, pH 8), incubated at 18 °C. To our satisfaction, denaturing gel electrophoresis revealed an 88% yield of the loop-closing ligation product after 24 h. We then systematically evaluated various factors that might influence ligation efficiency, including aldehyde type, catalyst type, pH, metal ion presence, and reagent concentrations (Figure 2 and Table 1).

**Figure 2:**
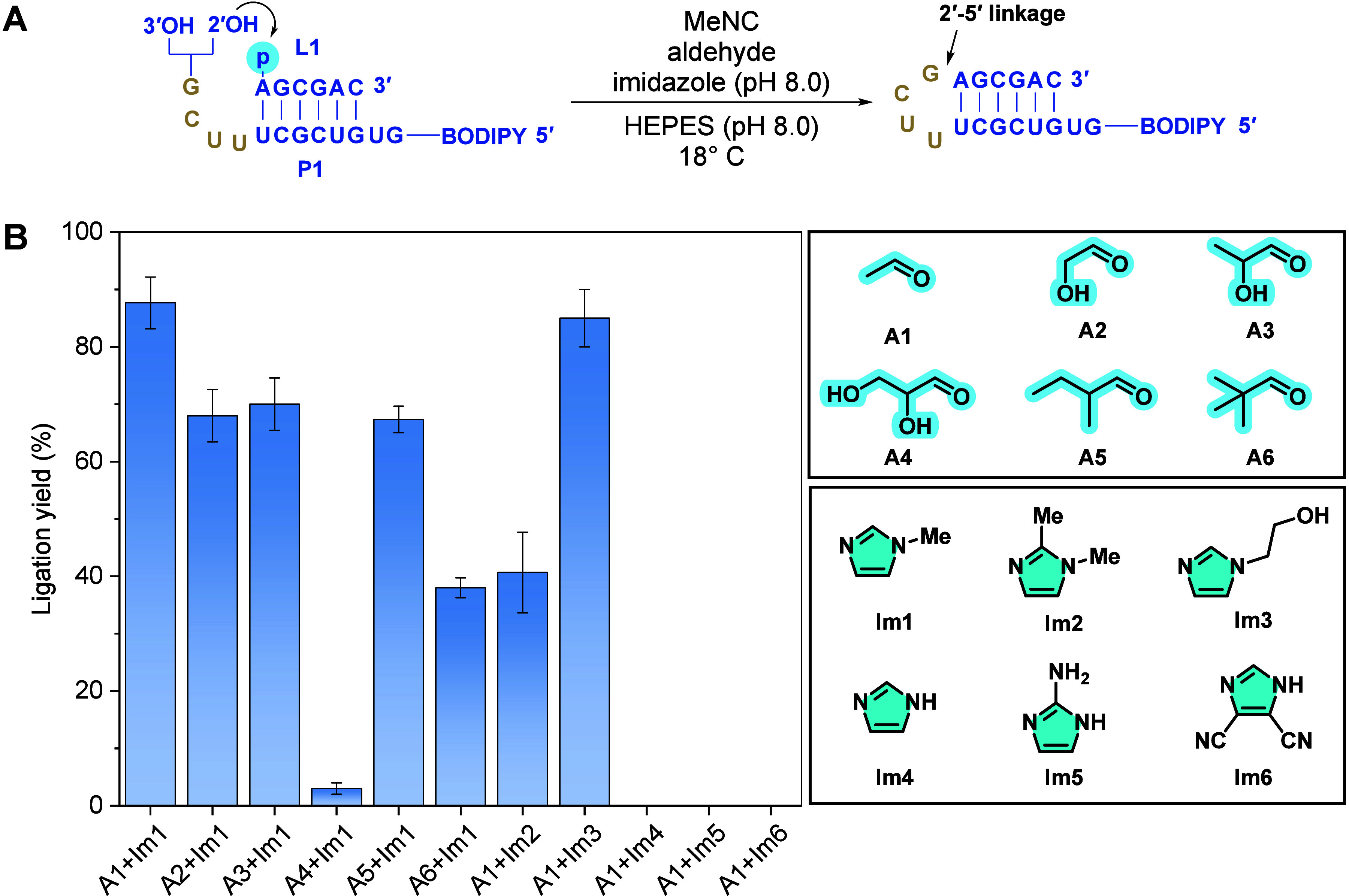
One-pot tandem reaction of oligonucleotide phosphate activation and loop-closing ligation. (A) Schematic representation of *in situ* loop-closing ligation. A, U, G, and C denote the four ribonucleotides; OH represents a hydroxyl group; p indicates a phosphate group. BODIPY serves as a fluorescent label. The loop-closing ligation proceeds predominantly through nucleophilic attack by the 2′-hydroxy group, resulting in a hairpin product with a 2′-5′ phosphodiester linkage. (B) Ligation yields observed with different combinations of aldehydes and imidazoles. The chemical structures of aldehydes and imidazoles are shown on the right. Reactions were conducted with a 12-nt primer P1 (1 μM), a 6-nt ligator L1 (2 μM), imidazole (200 mM, pH 8.0), HEPES (200 mM, pH 8.0), aldehyde (200 mM), and MeNC (200 mM) at 18 °C for 24 h. Error bars represent standard deviations from the mean, *n* = 3 replicates.

**Table 1:** Influence of pH, Metal Ions and Activating Agent Concentration on *in Situ* Activation and Ligation^a^

Entry	pH	Additive/mM	MeNC/mM	**A1**/mM	**Im1**/mM	Ligation yield (SD)/%
1	6.0		200	200	200	5(1)
2	6.5		200	200	200	31(2)
3	7.0		200	200	200	70(1)
4	7.5		200	200	200	87(2)
5	8.0		200	200	200	88(1)
6	8.5		200	200	200	71(2)
7	9.0		200	200	200	52(3)
8	8.0	LiCl/100	200	200	200	82(1)
9	8.0	NaCl/100	200	200	200	82(1)
10	8.0	KCl/100	200	200	200	82(2)
11	8.0	CaCl_2_/25	200	200	200	91(2)
12	8.0	MgCl_2_/25	200	200	200	83(1)
13	8.0	CaCl_2_/100	200	200	200	71(3)
14	8.0	MgCl_2_/100	200	200	200	59(3)
15	8.0	EDTA/1	200	200	200	82(2)
16	8.0^b^		100	100	200	65(1), 72(3)^c^
17	8.0^b^		50	50	200	86(2), 91(2)^c^
18	8.0^b^		25	25	200	92(2), 96(1)^c^
19	8.0^b^		10	10	200	84(3), 95(0)^c^
20	8.0^b^		5	5	200	55(4), 82(3)^c^
21	8.0^b^		2.5	2.5	200	23(1), 46(4)^c^
22	8.0^b^		1	1	200	4(0), 11(0)^c^
23	8.0^b^		25	25	5	13(8)
24	8.0^b^		25	25	25	27(8)
25	8.0^b^		25	25	50	48(11)
26	8.0^b^		25	25	100	76(6)
27	8.0^b^		25	25	400	93(1)

a Reactions were conducted using a 12-nt primer P1 (1 μM), 6-nt ligator L1 (2 μM) (see the sequences of P1 and L1 in Figure 2A), *N*-methylimidazole (200 mM, pH adjusted to match buffer pH), a buffer (200 mM, if added), acetaldehyde, and MeNC at 18 °C for 24 h. MES (2-(*N*-morpholino)­ethanesulfonic acid) buffer was used at pH 6.0 and 6.5; HEPES buffer was used at pH 7.0, 7.5, and 8.0; EPPS (4-(2-hydroxyethyl)­piperazine-1-propanesulfonic acid) buffer was used at pH 8.5 and 9.0.

b Reactions were performed without the addition of buffer, and *N*-methylimidazole solution was adjusted to pH 8.0.

c Yield after 3 days. Standard deviations (SD, *n* = 3) are reported at the appropriate significant digit in parentheses.

Steric and electronic properties of aldehydes significantly affect their reactivity in the Passerini-type reaction. To assess their impact on the activation-ligation system, we tested a series of structurally diverse aldehydes (Figure 2B and Figure S1). All tested *in situ* loop-closing ligation reactions yielded the expected ligated hairpin product, with varying efficiencies. Yields ranged from 3% for glyceraldehyde **A4** to 88% for acetaldehyde **A1** after 24 h. A reduced yield observed with **A4** is likely due to the rapid depletion of the nitrilium intermediate via intramolecular cyclization (Figure S2), which prevents effective phosphate activation. Extending the incubation time to 4 days did not significantly improve ligation yields (Figure S1), consistent with ^1^H NMR observations indicating that most activating reagents were consumed within 24 h (Figures S3 and S4). To confirm the identity of the ligation product, we performed mass spectrometry analysis (Figure S5), denaturing PAGE analysis with authentic synthetic standards (Figure S6), and negative control experiments (Figure S7). It is worth noting that the loop-closing ligation resulted in a 2′-5′ phosphodiester linkage in the hairpin product, rather than the natural 3′-5′ linkage (Figure S6). In the presence of a template that binds both primer (P1) and ligator (L1), enabling a nicked duplex (splint) ligation mode, a total ligation yield of 43% was obtained after 24 h, comprising 34% 3′-5′ linkage and 9% 2′-5′ linkage products (Figure S8). A nicked duplex excluding loop-closing ligation mode proceeded in a similar yield (40%, Figure S9), accompanied by a 3′-5′ to 2′-5′ linkage ratio of 3:1 (Figures S9 and S10). These results highlight the distinct geometric requirements for loop-closing ligation and template-directed ligation, and demonstrate the superiority of loop-closing ligation in assembling hairpin structures.

We then investigated the influence of different imidazole catalysts on reaction efficiency. Both *N*-methylimidazole **Im1** and *N*-hydroxyethylimidazole **Im3** achieved comparable yields 88% and 85%, respectively, after 24 h (Figure 2B). However, introducing a methyl group at the C-2 position of *N*-methylimidazole **Im2** reduced the yield to 41%, likely due to increased steric hindrance impairing the attack of **Im2** on the imidoyl-phosphate intermediate (Figure 1B, **V** to **VI**). In contrast, no ligation products were detected when imidazole **Im4**, 2-aminoimidazole **Im5**, or 4,5-dicyanoimidazole **Im6** were used as catalysts. This suggests that the formation of a positively charged phosphorimidazolium intermediate (Figure 1B, **VI**), rather than a neutral phosphorimidazolide, is crucial for subsequent loop-closing ligation. These findings align with previous observation that *N*-methylimidazole accelerates loop-closing ligation through the exchange of the imidazole leaving group in preactivated phosphorimidazolides.^21,22^

### Factors Impacting *in Situ* Phosphate Activation and Loop-Closing Ligation

The reaction proceeds effectively over a broad pH range (pH 6.0 to 9.0; Table 1, entries 1–7), with optimal yields observed at pH 7.5 and 8.0. Yields decrease sharply below pH 6.5, likely due to protonation of *N*-methylimidazole (p*K*_a_ = 7.2, Figure S11), which reduces its availability toward the imidoyl phosphate intermediate (Figure 1B, **V** to **VI**). Additionally, the nucleophilicity of the 2′,3′-diol is decreased under acidic conditions, limiting attack on the phosphorimidazolium intermediate. The yield also declines at pH values above 8.0, possibly due to several factors including (1) increased hydroxide ion concentrations accelerate hydrolysis of key intermediates, such as nitrilium and phosphorimidazolium species, (2) aldehyde activation, which generally requires protonation,^32^ may be inhibited at higher pH, and (3) deprotonation of uracil (U) and guanine (G) residues (p*K*_a_ ∼ 9.2 ^33^) might disrupt the favorable UUCG overhang conformation required for efficient ligation.

Divalent metal ions are essential for effective nonenzymatic RNA primer extension^30,31,34,35^ and ligation.^18,21–24,36^ However, the high concentrations of metal ions required for such reactions induce fatty acid precipitation and, therefore, appear to be incompatible with fatty acid based model protocell membranes.^26^ To assess the role of metal ions in *in situ* activation and loop-closing ligation, we tested a range of metal salts, including monovalent ions (LiCl, NaCl, KCl) and divalent ions (CaCl_2_, MgCl_2_). At moderate concentrations (100 mM for monovalent and 25 mM for divalent ions), no significant changes in ligation yield were observed (Table 1, entries 8–12). However, at higher concentrations (100 mM Mg^2+^ or Ca^2+^), ligation efficiency decreased (entries 13 and 14), likely due to increased hydrolysis of reactive intermediates **V** and **VI** (Figure 1B). To determine whether divalent metal ions played a catalytic role, we omitted divalent ions from the reaction mixture and added 1 mM EDTA to chelate and sequester any remaining divalent metal ions. The reaction yield was only slightly reduced in the presence of EDTA (88% to 83%; Table 1, entries 5 and 15), indicating that divalent metal ions such as magnesium are not essential for the reaction. This contrasts with systems that rely on preactivated phosphorimidazolide RNA, which require high magnesium concentrations for efficient phosphodiester bond formation.^21,22^

We then examined the effects of activating reagent concentration on ligation efficiency. From a prebiotic perspective, millimolar concentrations of MeNC are more plausible than the high concentrations (hundreds of mM) often employed in model prebiotic chemistry scenarios. To examine the role of reagent concentration, we omitted HEPES buffer to eliminate its influence and adjusted the *N*-methylimidazole solution to pH 8.0. This enabled us find ideal conditions for lower concentrations of activating agents (Table 1, entries 16–22). Reducing the concentrations of the activating reagents from 100 mM to 25 mM improved the ligation efficiency from 65% to 92% within 24 h. However, a further reduction to 1 mM resulted in only 4% yield after 1 day and 11% after 3 days. At higher activating reagent concentrations, chemical modifications were more prevalent, which might explain the smearing of the starting material and product bands during PAGE (Figure S12). Conversely, at lower concentrations, insufficient formation of nitrilium species appears to limit phosphate activation (Figure 1B, **IV** to **V**), thereby reducing ligation yields. Balancing these opposing effects, we found that the concentration of activating reagents required for effective ligation can be as low as 3–10 mM (Table 1, entries 19–21). Notably, a prebiotic reservoir of MeNC stored as an Fe (II) complex (∼ 20 mM) could release approximately 3 mM MeNC upon UV irradiation, providing a plausible prebiotic scenario for phosphate activation.^28,29^

Finally, we examined the effect of *N*-methylimidazole concentration on *in situ* activation and ligation using 25 mM activating reagents. As expected, increasing *N*-methylimidazole concentration from 5 mM to 200 mM improved the ligation yield from 13% to 92% after 1 day. However, no further improvement was observed for 400 mM *N*-methylimidazole, where the yield plateaued at 93% (entries 18 and 23–27, Table 1). This trend is consistent with the proposed mechanism, in which a sufficient concentration of *N*-methylimidazole is required to intercept a transient imidoyl phosphate intermediate and facilitate phosphorimidazolium formation (Figure 1B). In summary, under optimized conditions (pH 8.0, 18 °C, 25 mM activating reagents, and 200 mM *N*-methylimidazole), the model loop-closing ligation reaction achieves up to 92% yield within 24 h. Notably, this process occurs without the need for an external template or divalent metal ions.

### Mechanistic Investigation of *in Situ* Phosphate Activation Chemistry

In a stepwise reaction mechanism, the nitrilium species **3** that forms from nucleophilic attack by an isonitrile on an aldehyde (Figure 3A, step a) reacts with the phosphate species **5** (Figure 3A, step b) to yield imidoyl phosphate **6**.^28–31^ In the typical Passerini reaction pathway, imidoyl phosphate **6** undergoes intramolecular rearrangement to produce a dead-end phosphodiester product **7** (Figure 3A, step e). Both the nitrilium **3** and imidoyl phosphate **6** species are presumed to be transient and hydrolytically unstable in aqueous solution, yielding the byproduct *N*-methyl lactamide **8** upon hydrolysis (Figure 3A, steps c and f).^29^ However, in the presence of a highly nucleophilic imidazole, such as *N*-methylimidazole **4**, nucleophilic attack on the highly reactive imidoyl phosphate **6** can form the more stable phosphorimidazolium intermediate **9** (Figure 3A, step g). This intermediate subsequently facilitates phosphoryl group transfer reactions,^29–31^ including ligation, as demonstrated in this study. Supporting this mechanism, when methyl isocyanide (MeNC), acetaldehyde, and adenosine monophosphate (AMP) were combined, a moderate amount of Passerini product **7** was observed (Figure 3B, top). However, with the addition of *N*-methylimidazole, a substantial accumulation of phosphorimidazolium **9** occurred within 2 h (Figure 3B, bottom).

**Figure 3:**
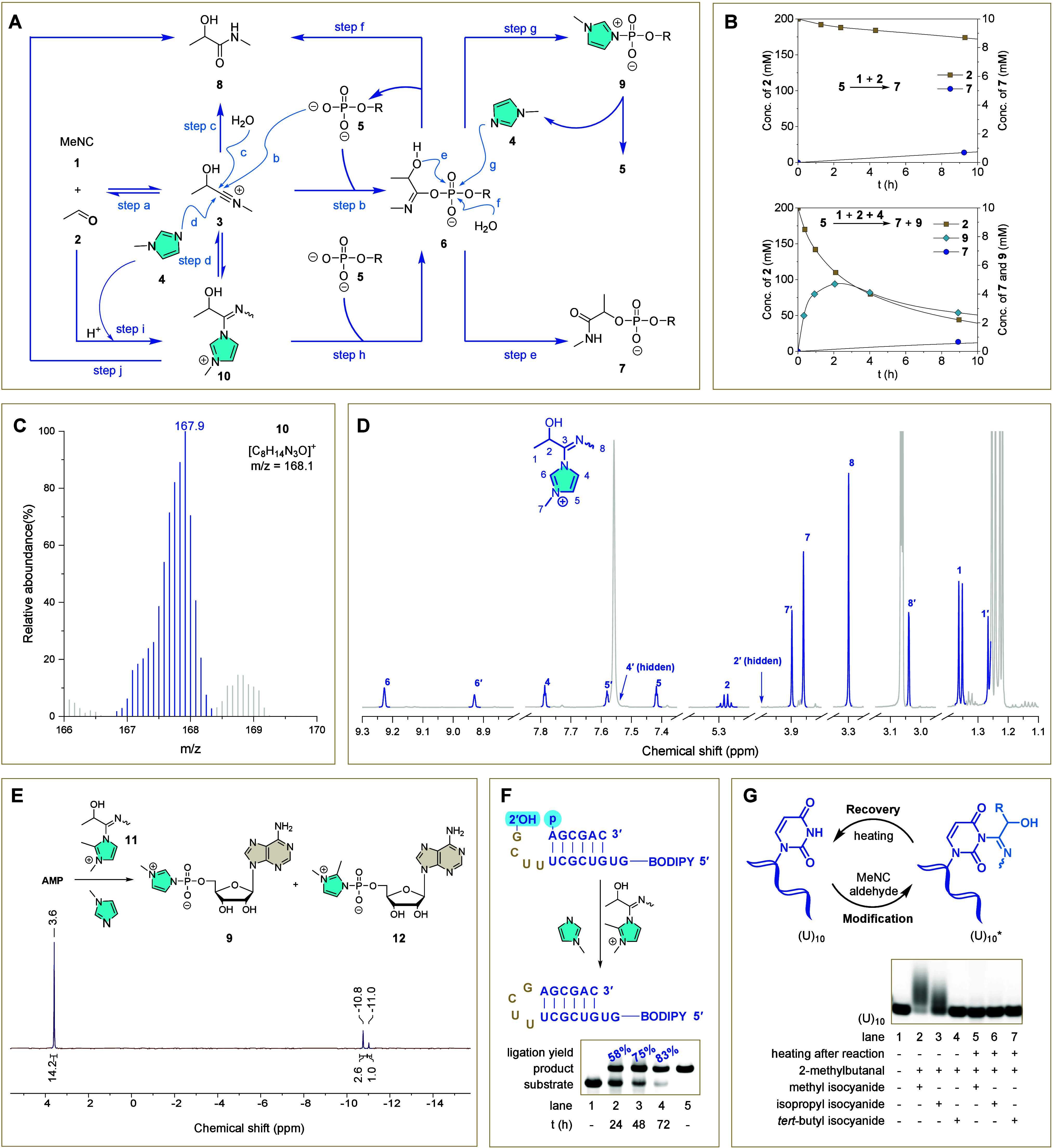
Mechanistic details of the *in situ* phosphate activation and competing side-reactions. (A) Previously proposed mechanism of phosphate activation via the Passerini-type reaction and competing pathways. (B) Reactions of MeNC (**1**, 200 mM), acetaldehyde (**2**, 200 mM), and AMP (**5**, 10 mM) at room temperature, in the absence (top) and presence (bottom) of *N*-methylimidazole (**4**, 200 mM) were monitored by ^1^H NMR and ^31^P NMR. (C) Mass spectrum of the IMI **10**. The observed mass (*m*/*z*: 167.9) matches the calculated mass (*m*/*z*: 168.1) for the imidazolium intermediate **10**, corresponding to [C_8_H_14_N_3_O]^+^. (D) ^1^H NMR spectrum of IMI **10** with assigned proton signals for each of the two isomers. The minor isomer is denoted with prime notation. (E) ^31^P NMR spectrum showing AMP activation by IMI **11**, leading to phosphorimidazolium formation. The reaction mixture contained 25 mM AMP (pH 8.0), 200 mM *N*-methylimidazole **4** (pH 8.0), 150 mM IMI **11**, and 10% D_2_O, and was monitored by ^31^P NMR. After 2 h, yields of 6% (**9**, 11.0 ppm) and 15% (**12**, 10.8 ppm) were observed, with a total AMP conversion of 21% (3.6 ppm), calculated from ^31^P NMR signal integration (ratio of AMP:**9**:**12** = 14.2:1:2.6). (F) The *in situ* loop-closing ligation enabled by IMI **11**. Upon the addition of IMI **11** as an activating reagent, the loop-closing hairpin product was obtained with yields of 58% (day 1, lane 2), 75% (day 2, lane 3), and 83% (day 3, lane 4). The reaction mixture consisted of P1 (1 μM), L1 (2 μM), *N*-methylimidazole **4** (200 mM), and IMI **11** (20 mM). Lanes 1 and 5 show the fluorescently labeled substrate and authentic ligation product reference, respectively. (G) Base modification and thermal recovery of (U)_10_ oligonucleotide. The (U)_10_ oligonucleotide underwent modification in the presence of methyl or isopropyl isocyanide (lanes 2 and 3) due to nucleophilic attack of uridine N3 on the nitrilium ion, resulting in a smeared appearance in PAGE bands (U)_10_*. These reversible modifications were removed by heating at 95 °C for 15 min (lanes 5 and 6). Reaction conditions for modification: (U)_10_ (1 μM), isocyanide (200 mM), 2-methylbutanal (200 mM), HEPES (200 mM, pH 8.0), rt, 24 h.

We observed that acetaldehyde consumption was faster in the presence of imidazole (Figure 3B, bottom vs top), prompting further investigation. To examine this effect, we monitored the control reaction of methyl isocyanide (**1**, 25 mM) with acetaldehyde (**2**, 25 mM) using ^1^H NMR. After 23 h, less than 10% of the starting materials had converted into the expected byproduct, *N*-methyl lactamide **8**. Surprisingly, when the reaction was conducted in the presence of imidazole, *N*-methylimidazole, 1,2-dimethylimidazole, or HEPES (Figures S13–S15), acetaldehyde **2** was consumed at a significantly higher rate which led to faster formation of compound **8**. When catalyzed by *N*-methylimidazole or 1,2-dimethylimidazole, new species accumulated before gradually hydrolyzing. Mass spectrometry and NMR spectroscopy identified these species as the *N*-imidoyl-*N*′-methylimidazoliums (IMI) **10** and **11** (Figures 3C, 3D, and S26–47).^37,38^ We propose that *N*-methylimidazole functions as a catalyst, promoting the formation of compound **8** via IMI intermediates (**10** or **11**), which are generated either through a stepwise mechanism (Figure 3A, steps a and d) or a concerted reaction mechanism (Figure 3A, step i). This, in turn, accelerates aldehyde consumption (Figure 3A, step j).

While the IMI **10** is too labile to be isolated (Figure S14), IMI **11** is stable enough (Figure S15) to be semipurified by evaporating volatile components (isocyanide **1**, aldehyde **2**, and H_2_O) from the reaction mixture containing MeNC **1**, acetaldehyde **2** and 1,2-dimethylimidazole under reduced pressure (Supplementary methods). This suggests that, if reversible, the potential equilibrium between IMI **11** and a mixture of **3** and 1,2-dimethylimidazole is strongly biased toward the formation of IMI **11** during the evaporation process. The isolation of **11** prompted us to question whether the IMI intermediates lead to dead-end products by consuming activating agents, or if they can instead function as nitrilium surrogates for phosphate activation (Figure 3A, step h or steps d-b). When semipurified intermediate **11** was tested for its ability to activate adenosine 5′-phosphate (AMP), it successfully generated the 5′-phosphorimidazolium species (**9** and **12**, Figure 3E). Furthermore, when intermediate **11** was used to drive loop-closing ligation, 58% of the ligated product was observed after 1 day, with an improved yield of 84% after extending the reaction time by 2 days (Figure 3F). In conjunction with the observation that IMI **11** does not readily revert to **3** and 1,2-dimethylimidazole, these findings clearly demonstrate that the newly identified IMI intermediates serve as surrogates for nitrilium **3** in phosphate activation within aqueous solutions (Figure 3A, step h).

### Reversibility of Nucleobase Modification under *in Situ* Activation Conditions

As noted above, RNA is likely to undergo chemical modification under our *in situ* activation conditions (Figure S12). It is well established that uridine (U) and guanosine (G) are highly susceptible to base modification by carbodiimides at pH 8 due to the nucleophilicity of their respective *N*3 and *N*1 positions,^39^ which have p*K*_a_ values of approximately 9.2.^33^ When not engaged in base pairing, these nucleobases act as nucleophiles upon deprotonation and can attack activating reagents. This property has been widely utilized in RNA structural probing.^40,41^ Under our *in situ* activation conditions, we observed that RNA modifications were severe and depended on the presence of both methyl isocyanide (MeNC) and aldehyde but not on other additives (Figure S16). To identify the modification sites, we incubated fluorophore-labeled oligonucleotide 10-mers, (A)_10_, (C)_10_, (U)_10_, and (GA)_5_, with 200 mM isocyanide and 2-methylbutanal at pH 8.0 at room temperature for 24 h (Figure 3G and S17). RNA gel electrophoresis revealed no apparent modifications for (A)_10_ and (C)_10_, whereas (U)_10_ was heavily modified (Figures 3G, lanes 2 and 3), and (GA)_5_ exhibited slight modification (Figure S17). These results indicate that the sites of modification are not the internal 2′-hydroxyl groups of RNA but rather the nucleobases U and G, as with RNA modification by carbodiimides.^39^

To further confirm the nature of the modifications, individual nucleoside monophosphates (A, U, G, C) were subjected to activation conditions and monitored by ^1^H NMR and ^31^P NMR (Figures S18 and S19). Uridine monophosphate was converted into a new species after 18 h, which was determined to be *N*3-imidoyl uridine formed via nucleophilic attack by deprotonated N3 on the reactive nitrilium species (Figure S20). Notably, *N*3-imidoyl uridine was thermally unstable, hydrolyzing back to uridine after 8 days at room temperature (Figure S21), with the process accelerating to 30 min upon heating at 95 °C (Figures S22). While guanosine monophosphate showed no significant change after heating under same conditions (Figure S22), the 4% modification observed in (GA)_5_ was reduced to 1% following heating (Figure S17). Importantly, chemically modified side products of loop-closing ligation were converted into the desired ligation products without significant degradation when heated at 95 °C for 3 min (Figure S23). These findings suggest that RNA modification is likely unavoidable under continuous nonenzymatic *in situ* activation conditions. However, modifications can be minimized through optimized reaction conditions and postreaction hydrolysis, potentially mirroring fluctuating environmental conditions on the early Earth.^42–44^

### The *in Situ* Activation Enhances Efficiency Compared to Stepwise Preactivation

Previously established RNA ligation methods relied on the presynthesis and purification of 5′-phosphorimidazolide RNA, followed by reaction with a second RNA strand containing an overhang to facilitate loop-closing ligation.^21,22^ Optimal yields in this process require both high concentrations of *N*-methylimidazole and divalent metal ions. However, under these conditions, ligation yields rarely exceed 65%, even with the best overhang sequences.^22^ In contrast, the *in situ* activation approach developed here significantly simplifies the process. By directly mixing unactivated substrate RNAs with MeNC, an aldehyde, and *N*-methylimidazole, the ligation efficiency is markedly improved. To compare the two strategies, previously tested loop-closing ligation constructs were subjected to *in situ* activation conditions. Remarkably, yields of 96% and 81% were achieved for UUCG and CAGG overhangs, respectively (Figure 4), whereas the preactivation approach yielded only 69% and 28% for the same overhangs.^22^ These findings underscore the substantial advantages of *in situ* activation, demonstrating its superior efficiency and practical applicability due to significantly improved ligation yields and the streamlined procedure.

**Figure 4:**
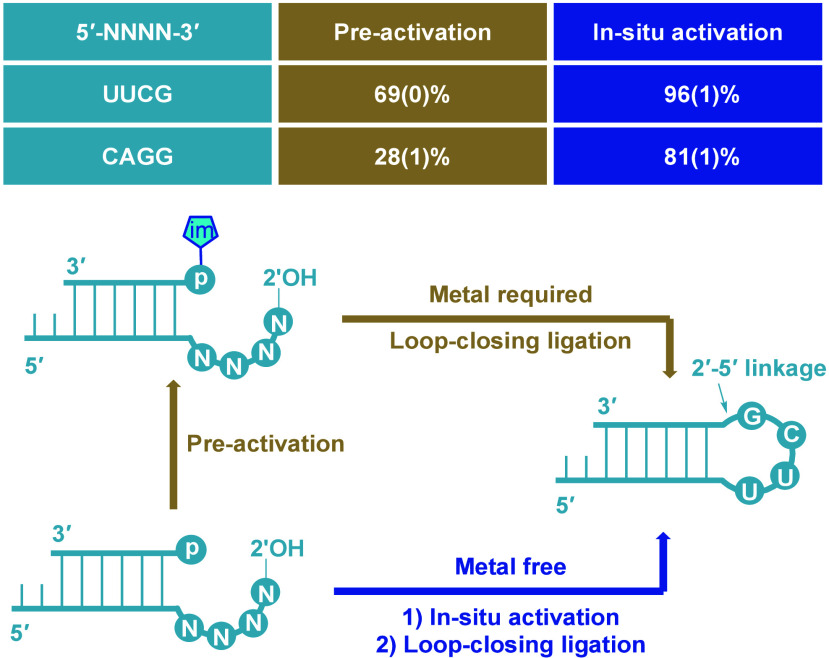
Comparison of loop-closing ligation via preactivation and *in situ* activation. RNA assembly via the preactivation pathway requires two separate steps: Activation of oligonucleotides to generate 5′-phosphorimidazolide (Im-p-RNA) intermediates, followed by loop-closing ligation in the presence of magnesium ions. In contrast, the *in situ* activation and loop-closing ligation approach is performed in a one-pot reaction, eliminating the need for intermediate purification and proceeding in the absence of metal ions. Reaction conditions for preactivation ligation: 12-nt primer P1 or P2 (1 μM), activated 6-nt ligator Im-p-L1 (1 μM), MgCl_2_ (50 mM), *N*-methylimidazole (200 mM, pH 8.0), NaCl (100 mM) at room temperature (18 °C) for 4 h. Reaction conditions for *in situ* loop-closing ligation: 12-nt primer P1 or P2 (1 μM), 6-nt ligator L1 (2 μM), *N*-methylimidazole (200 mM, pH 8.0), acetaldehyde (25 mM), and MeNC (25 mM) at room temperature (18 °C) for 3 days. Oligonucleotide sequences (P1, P2, and L1) are shown in Table S1. The 5′-NNNN-3′ represents the overhang sequence of primer P1 or P2. Standard deviations (SD, *n* = 3) are reported at the appropriate significant digit in parentheses.

### Near-Quantitative Assembly of Functional RNA via Multistep *in Situ* Loop-Closing Ligations

Flexizyme (**dFx**), an *in vitro*-evolved ribozyme,^45^ has been developed as an efficient tool for RNA 3′-OH aminoacylation^46^ and genetic code reprogramming.^47^ In this study, we employed a mutated Flexizyme (**dFx-mut**) as a model to evaluate the efficacy of *in situ* activation and loop-closing ligation. To facilitate this process, **dFx-mut** was split at its loop regions into three parts (**F1**, **F2**, and **F3**) for sequential ligation (Figure 5A, left). These fragments were incubated at pH 8 with MeNC, acetaldehyde, and *N*-methylimidazole at 18 °C. After 2 days, products with one or two loops closed were observed in 70% and 18% yields respectively (lane 2, Figure 5A, right). The high efficiency of ligation of the loop between **F1** and **F2** (88% in total of **F1**–**F2** and **F1**–**F2**–**F3**) contrasts with the low efficiency of loop-closing ligation between **F2** and **F3** (18% observed for **F1**–**F2**–**F3**). We suspected that the bulky triethylammonium counterion introduced during the desalting stage of oligomer synthesis, along with competing interactions such as **F3** self-dimer formation, might have decreased pairing between **F2** and **F3**. The addition of 100 mM NaCl substantially enhanced efficiency, yielding 84% of the complete product **F1**–**F2**–**F3** and only 9% of the partial product **F1**–**F2** (lane 3, Figure 5A, right). While increasing the concentration of activating reagents to 100 mM did not improve the yield (lane 4, Figure 5A, right), splitting the addition of the activating agents into two portions (one per day) led to the more efficient assembly of three short fragments (**F1**, **F2**, **F3**) of the mutated Flexizyme (**F1**–**F2**–**F3**) in 90% yield (lane 5, Figure 5A, right).

**Figure 5:**
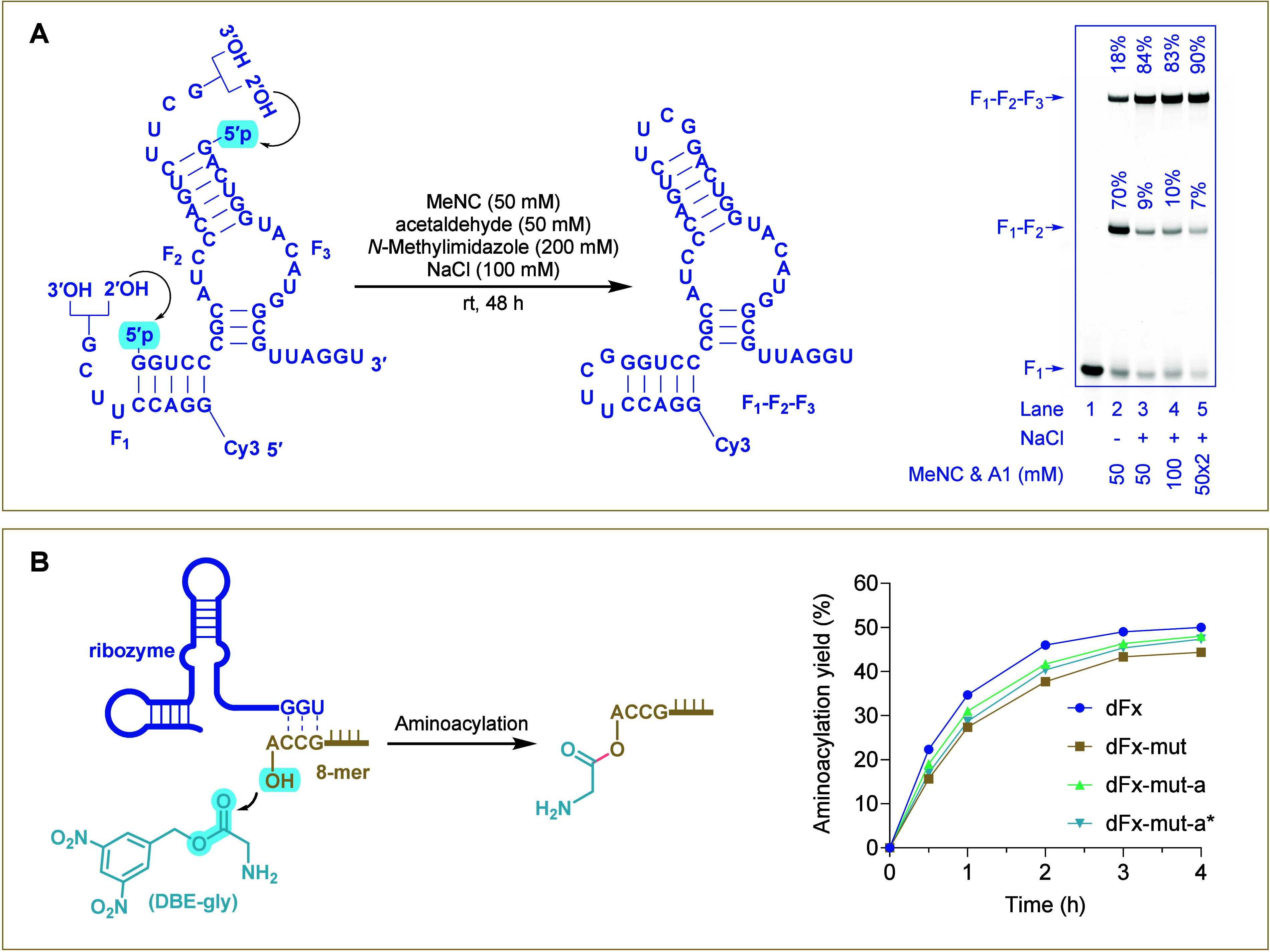
Assembly and activity evaluation of functional RNA. (A) Schematic representation of **dFx-mut** assembly from three shorter oligomers (**F1**, **F2**, and **F3**) via sequential phosphate activation and loop-closing ligation is shown on the left. The **F1** was labeled at its 5′-end with a Cy3 fluorophore to monitor ligation reactions. On the right, a denaturing PAGE image displays the ligation reactions under the following conditions: **F1** (1 μM), **F2** (2 μM), **F3** (4 μM), *N*-methylimidazole (200 mM, pH 8.0), NaCl (100 mM), acetaldehyde, and MeNC, incubated at room temperature for 48 h. The notation 50 × 2 indicates that two batches of activating agents (50 mM acetaldehyde and 50 mM MeNC each) were added separately at time points 0 and 24 h. (B) Flexizyme-catalyzed aminoacylation of an RNA 8-mer by DBE-gly is represented on the left. The right panel shows aminoacylation yields over time for four different ribozyme samples: **dFx**, a reference ribozyme with previously demonstrated catalytic efficacy. **dFx-mut**, a mutated version prepared via RNA synthesizer. **dFx-mut-a**, a mutated version prepared via *in situ* phosphate activation and loop-closing ligation. dFx-mut-a*, dFx-mut-a after heating-treatment at 95 °C for 1 h to remove potential base modifications introduced during *in situ* activation and ligation. Reaction conditions for aminoacylation: 8-mer RNA substrate (0.5 μM), Flexizyme (0.5 μM), HEPES (100 mM, pH 8.0), MgCl_2_ (100 mM), DBE-gly (5 mM) at 0 °C. Yields represent the mean values from triplicate experiments.

Next, we assessed the catalytic activity of the *in situ*-assembled Flexizyme (Figures 5B and S24), considering both the incorporation of UUCG motifs into the loop regions of **dFx-mut** and potential chemical modifications. Two reference ribozymes were used as controls: **dFx** and **dFx-mut**, both synthesized with standard 3′-5′ phosphodiester linkages by solid phase RNA synthesis. **dFx-mut** retained 70% of **dFx** activity based on corresponding yields at 0.5 h, confirming that substituting the loop sequences with UUCG does not greatly impair ribozyme function. Similarly, the *in situ*-assembled full-length Flexizyme, whether heat-untreated (**dFx-mut-a**,) or heat-treated (dFx-mut-a*), exhibited comparable activity to **dFx-mut** (Figure 5B). A comparison between **dFx-mut** and dFx-mut-a* demonstrated that the 2′-5′ phosphodiester linkages present in dFx-mut-a* did not affect the Flexizyme activity.^21,48^ The comparable activity observed between **dFx-mut-a** and dFx-mut-a* suggests that the modification of the former was minimal. These findings demonstrate that none of the UUCG loop mutations, 2′-5′ phosphodiester linkages, or minor chemical modifications significantly impact ribozyme function. This study establishes that functional RNA can be efficiently assembled from shorter fragments through *in situ* phosphate activation and multistep loop-closing ligations, offering a scalable strategy for RNA synthesis.

## Discussion

In this study, we investigated the assembly of long, functional RNAs from short RNA fragments. Unlike previous work that employed preactivated phosphorimidazolides,^18–22^ RNA fragments here were activated *in situ* by methyl isocyanide and aldehydes, addressing the potential for prebiotic accessibility of activated RNAs. The resulting activated phosphorimidazolium can be regenerated after hydrolysis, thus allowing loop-closing ligation to proceed to completion. This approach requires only methyl isocyanide, an aldehyde, and an *N*-methylimidazole catalyst, and is independent of divalent metal ions, which can be detrimental to RNA or fatty acid membranes. As a model for functional RNAs, a mutated Flexizyme was successfully assembled in near-quantitative yield through loop-closing ligation events at multiple ligation sites, enabling access to more complex RNA architectures. Collectively, our findings demonstrate the efficacy of *in situ* phosphate activation coupled with loop-closing ligation for the assembly of native, functional RNAs.

Previously, RNA fragments bearing highly nucleophilic amino functionalities have been employed to assemble longer RNAs through either template-directed ligation or template-free loop-closing ligation.^19,20,49^ In some of these reactions, magnesium ions modestly enhance the efficiency of ligation, probably by facilitating proper folding or base pairing. For loop-closing ligation employing preactivated phosphorimidazolides,^21,22^ both Mg^2+^ and *N*-methylimidazole are required for high efficiency, with a 69% yield observed within 4 h (Figure S25). Extraordinarily, the *in situ* ligation examined in this study was found to be independent of divalent metal ions. In fact, either Mg^2+^ or *N*-methylimidazole can promote loop-closing ligation with preactivated substrates, though less effectively. When used individually, *N*-methylimidazole yields 30% while Mg^2+^ yields 13% after 4 h (Figure S25). The catalytic magnesium ions in the ligation reactions may act as (1) Lewis acids to coordinate the phosphorimidazolide oxygen or the hydroxyl nucleophile, or (2) general bases to deprotonate the terminal 2′,3′-diol. As for *N*-methylimidazole, it enhances ligation efficiency by forming a more electrophilic phosphorimidazolium that possesses a permanent positive charge. Provided that the activating agents are present, the active 5′-phosphorimidazolium species can be continuously regenerated after hydrolysis under *in situ* activation conditions. This persistent regeneration facilitates efficient loop-closing ligation with the 5′-phosphorimidazolium intermediate, overshadowing the minor catalytic contribution of Mg^2+^ (Figure S25 and Table 1, entries 5 and 12).

The efficient loop-closing ligation in the absence of divalent metal ions would enhance compatibility with fatty acid protocells, which are unstable even at low millimolar concentrations of divalent metal ions.^26^ Nonenzymatic RNA copying by primer extension requires high concentrations of divalent metal ions,^30,31^ such as Mg^2+^, Mn^2+^ or Fe^2+^.^34,35^ We envisage that an RNA copying chemistry requiring low millimolar concentrations of divalent metal ions would enable continuous replication and joining of short RNAs, in line with the potentially prebiotic chemistry described here. A low metal system could also facilitate the nonenzymatic RNA copying and loop-closing ligation coherently inside a model protocell composed of fatty acids.

The investigation of phosphate activation mechanism allowed us to uncover a previously unrecognized phosphate activation pathway involving a key *N*-imidoyl-*N*′-methylimidazolium (IMI) intermediate. Early research shows that imidazole accelerates the hydrolysis of imido esters by nucleophilic catalysis.^50^ Here we demonstrate that imidazole promotes the activation of phosphate through IMI intermediates. These IMI intermediates are reminiscent of azole-stabilized immonium salts,^37^ which have been employed in peptide synthesis. Our results provide a new method for developing activating reagents that are compatible with aqueous conditions. Similar to the formation of IMIs, the nucleophilic bases, especially uridine, undergo modification during continuous *in situ* activation. However, most modified moieties can be efficiently reversed via hydrolysis without RNA degradation. This reversible modification could be beneficial because it would slow down the reannealing of complementary RNA strands following strand separation and modification. This, in turn, could facilitate downstream processes such as template-directed copying and ligation, as unmodified regions remain accessible for short oligonucleotide binding and subsequent reactions. Our finding highlights the robustness of our approach in maintaining RNA integrity under prolonged, potentially prebiotic activation conditions. Compared to the stepwise preactivation and ligation procedure, the *in situ* approach streamlines experimental handling while achieving significantly higher yields. The near-quantitative ligation developed here holds promise for the nonenzymatic synthesis of long RNAs, offering a scalable and efficient method for assembling functional RNA molecules.^51,52^

## Supplementary Material



## Notes

Materials and methods, Supporting Figures and Tables, Denaturing Urea-PAGE images, NMR spectra (PDF)

## #

Verdox, 1 Gill Street, Woburn, Massachusetts 01801, United States
