# Supplementary material for: Efficient Assembly of Functional RNA by in Situ Phosphate Activation and Loop-Closing Ligation
Source: J Am Chem Soc. 2025 Oct 20;147(43):39212–22. doi: 10.1021/jacs.5c10478 (PMC12576782; doi:10.1021/jacs.5c10478)
Supplement: Supplementary file 1 [file ja5c10478_si_001.pdf]

Supporting Information

Efficient Assembly of Functional RNA by *In Situ* Phosphate Activation and Loop-Closing Ligation

Jian Zhang[1], Aleksandar Radakovic[2], Filip Bošković[1], Harry R. M. Aitken[3,4], Long-Fei Wu[5]* and Jack W. Szostak[1]*

[1]Howard Hughes Medical Institute, Department of Chemistry, The University of Chicago, Chicago, Illinois 60637, USA.

[2]Department of Ecology and Evolution, The University of Chicago, Chicago, Illinois 60637, USA.

[3]Howard Hughes Medical Institute, Massachusetts General Hospital, Boston, Massachusetts 02114, USA.

[4]Department of Chemistry and Chemical Biology, Harvard University, 12 Oxford Street, Cambridge, Massachusetts 02138, USA.

[5]Frontiers Science Center for Transformative Molecules, State Key Laboratory of Synergistic Chem-Bio Synthesis, School of Chemistry and Chemical Engineering, Zhangjiang Institute for Advanced Study, Shanghai Jiao Tong University, Shanghai, China

*To whom correspondence should be addressed; Email: lfwu@sjtu.edu.cn and jwszostak@uchicago.edu

Table of Contents

Materials and General 4

Polyacrylamide gel electrophoresis 5

Oligonucleotide synthesis 5

Fluorophore labeling of oligonucleotides 6

General operation of one-pot *in situ* phosphate activation and loop-closing ligation 6

Procedures of template-directed ligation 7

General operation of loop-closing ligation by pre-activation strategy 9

Synthesis of *N*-imidoyl-*N*′-methylimidazolium (IMI) **11** 9

AMP activation by *N*-imidoyl-*N*′-methylimidazolium (IMI) **11** 10

Loop-closing ligation employing *N*-imidoyl-*N*′-methylimidazolium (IMI) **11** 10

Assembly of Flexizyme by *in situ* loop-closing ligations 10

Activity evaluation of Flexizymes 11

Screening of aldehydes for *in situ* activation 12

Proposed mechanism of nitrilium ion depletion via intramolecular cyclization 13

Activation reagents consumption over time 14

Yields of *in situ* loop-closing ligation over time 15

Characterization of the hairpin product of *in situ* activation and loop-closing ligation 16-18

  High resolution mass spectra 16

  Comparison with authentic samples 17

  Regioselectivity validation by control experiments 18

Results of template-directed ligation 19-21

  Template-directed ligation of P1 and L1 19

  Template-directed ligation of P3 and L1 20-21

pK_a measurement of *N*-methylimidazole 22

RNA modification by *in situ* activation chemistry revealed by denaturing PAGE 23

Passerini-type reaction promoted by imidazoles and HEPES 24

Passerini-type reaction promoted by *N*-methylimidazole 25

Passerini-type reaction promoted by 1,2-dimethylimidazole 26

Nucleobase modification by *in situ* activation 27-34

  Modification of oligonucleotides P1 27

  Modification of oligonucleotides 10-mers $(A)_{10}$, $(U)_{10}$, $(C)_{10}$, $(GA)_5$ 28

  ^{1}H NMR of mononucleotides treated with activating reagents 29

  ^{31}P NMR of mononucleotides treated with activating reagents 30

  Proposed modification mechanism 31

Hydrolysis of modified uridine monophosphate over time at room temperature 32
Accelerated hydrolysis of modified uridine monophosphate by heating 33
Recovery of oligonucleotides by heating 34
Denaturing PAGE image of Flexizyme-catalyzed aminoacylation. 35
Loop-closing ligation using pre-activated Im-p-L1 36
Characterization of *N*-imidoyl-*N′*-methylimidazolium (IMI) **10** 37-46
^{1}H NMR 37
^{13}C NMR 38
2D NMR (HMBC, HSQC, H-H COSY) 39-46
Characterization of *N*-imidoyl-*N′*-methylimidazolium (IMI) **11** 47-58
^{1}H NMR 47
^{13}C NMR 48
2D NMR (HMBC, HSQC, H-H COSY) 49-58
Characterization of Passerini product **7** 59-63
^{1}H NMR 59
^{31}PNMR 60
^{13}CNMR 61
H-H COSY 62
HSQC 63
Mass spectrum of phosphorimidazolium 64
Table S1. Sequences of oligoribonucleotides in this work 65-66
Table S2. Chemical structures of fluorescently labeled oligoribonucleotides 67
References 68

Materials and General

Methyl isocyanide (or methyl isonitrile) was prepared according to literature procedures[1], and the concentration of the methyl isocyanide stock solution (0.5 M or 1 M in water) was calibrated by NMR using trimethyl phosphate as the internal standard. For safety, reactions involving isocyanide were carried out in a well-ventilated fume hood, as was polyacrylamide gel electrophoresis (PAGE). Acetaldehyde and *N*-methylimidazole were obtained from Sigma-Aldrich. Acetaldehyde is highly volatile, so it was used as an aqueous solution, with its concentration determined by NMR using trimethyl phosphate as the internal standard. The *N*-methylimidazole (or other imidazole) stock solutions (1 M or 2 M) were adjusted to appropriate pH before use. DBE-gly (3,5-dinitrobenzyl ester of glycine) was synthesized as reported previously[2]. Water used for HPLC was purified using a Millipore® EQ-7000 system. Ambion™ nuclease-free water for reactions was purchased from Thermo Fisher Scientific. BODIPY FL NHS ester was purchased from Thermo Fisher Scientific (Catalog number: D2184) or Sigma-Aldrich (Catalog number: SML3990). Exonuclease T (M0265L) and NEBuffer™ 4 (B7004S) were purchased from New England Biolabs. Other reagents and solvents were obtained from Acros Organics, Combi-Blocks, Sigma-Aldrich, Tokyo Chemical Industry Co., or Thermo Fisher Scientific, and were used without further purification. All solutions were aqueous unless otherwise specified.

RNA oligomers were synthesized using an Expedite 8909 Oligo Synthesizer at a 1 µmol scale and purified by denaturing PAGE or reverse-phase HPLC. Phosphoramidites used for RNA synthesis were purchased from Glen Research and ChemGenes. 1 µmol scale pre-packed synthesis columns were acquired from Glen Research. Additional RNA oligomers were purchased from Integrated DNA Technologies (IDT) and purified by PAGE.

All pH adjustments were made using a SevenCompact™ pH/Ion S220 equipped with an Orion™ ROSS™ PerpHecT™ Micro Glass Bodied Combination pH Electrode (8220BNWP from Thermo Fisher Scientific). Nuclear magnetic resonance spectra (^{1}H NMR, ^{13}C NMR, ^{31}P NMR, ^{1}H-^{1}H COSY, HSQC, HMBC) were recorded with Bruker Model DMX 400, 500, or 600. The HOD (residual water) suppression technique was used to minimize interference from the water signal when samples were suspended in a 90% H_2O/10% D_2O mixture for ^{1}H NMR analysis. Chemical shifts (δ) are shown in ppm. Coupling constants (*J*) are given in Hertz (Hz), and the notations s, d, and m represent singlet, doublet, and multiplet signals, respectively. For time-course

experiments, the reaction mixtures were incubated in NMR tubes and monitored by ^{1}H NMR and/or ^{31}P NMR. High-resolution mass spectra (HRMS) were recorded on an Agilent 6230 time-of-flight (TOF) mass spectrometer. Low-resolution mass spectra (LRMS) were recorded on an LTQ XL™ linear ion trap mass spectrometer.

Polyacrylamide gel electrophoresis (Denaturing urea-PAGE)

Analytical polyacrylamide gel electrophoresis was performed using 20% polyacrylamide gels containing 8 M urea (0.75 mm thick, 20 cm long) at 20 W in 1X TBE buffer (89 mM Tris-borate, 2 mM EDTA, pH 8.3) for 1 to 2 hours. Fluorescently labeled RNA oligomers were imaged with an Amersham RGB Biomolecular Imager (GE Healthcare Life Sciences, Marlborough, MA) and quantified with ImageQuant™ (GE Healthcare Life Sciences, Marlborough, MA). For oligonucleotide purification, a 1.5 mm thick gel was used.

Oligonucleotide synthesis

Oligonucleotides were either purchased from Integrated DNA Technologies or synthesized in-house using an Expedite 8909 solid-phase oligonucleotide synthesizer. 1 µmol scale pre-packed synthesis columns were obtained from Glen Research. Phosphoramidites and reagents for the Expedite synthesizer were purchased from Glen Research or ChemGenes. Cleavage from the solid support was carried out using 1 mL of AMA (a 1:1 mixture of 28% aqueous ammonium hydroxide and 40% aqueous methylamine) for 30 minutes at room temperature, followed by deprotection in the same solution for 30 minutes at 65 °C. The deprotected oligonucleotides were lyophilized, resuspended in 100 µL of dimethyl sulfoxide (DMSO), and treated with 125 µL of triethylamine (TEA) trihydrofluoride at 65 °C for 2.5 hours to remove *tert*-butyldimethylsilyl protecting groups from the 2′-hydroxyls. After deprotection, oligonucleotides were purified by preparative 20% polyacrylamide gel electrophoresis, desalted using Waters Sep-Pak C18 cartridges, and characterized by high-resolution mass spectrometry on an Agilent 6230 time-of-flight (TOF) mass spectrometer.

Fluorophore labeling of oligonucleotides

BODIPY FL NHS ester (12.5 µL, 10 mM in DMSO) was added to a solution containing 5′-amino modified oligonucleotides (P1-amino, P2-amino, P3-amino, H1-3′o-amino, H1-2′o-amino, H2-3′o-amino, H2-2′o-amino, as listed in Table S1) and sodium borate buffer (pH 8.5). The final concentrations were 100 µM 5′-amino modified oligonucleotide, 100 mM sodium borate, and 2.5 mM BODIPY™ FL NHS ester in a total volume of 50 µL. The reaction mixture was wrapped in aluminum foil to protect it from light and incubated for 24 hours at room temperature. To precipitate the labeled oligonucleotide, NH_4OAc (5 M, 40 µL) and isopropanol (600 µL) were added, and the mixture was cooled on dry ice for 10 minutes. After centrifugation at 21,130 rcf (relative centrifugal force) for 5 minutes, the supernatant was decanted. The resulting pellet was washed twice with cold 80% ethanol (2 × 400 µL) and dried under reduced pressure for 10 minutes. The solid residue was resuspended in water (20 µL) and was used without further purification.

General operation of one-pot *in situ* phosphate activation and loop-closing ligation

A reaction mixture containing a primer (1 µM), ligator (2 µM), buffer (0-200 mM), additive (0-100 mM), imidazole catalyst (5-400 mM), aldehyde (1-200 mM) and methyl isonitrile (1-200 mM)[a], was incubated at room temperature (18 °C) in a well-ventilated fume hood. 1 µL of the reaction mixture was quenched in 9 µL buffer containing 90% formamide and 50 mM EDTA (pH 8.0) at 24 hour intervals[b]. The quenched solution was heated at 95°C for 3 minutes[c] and analyzed by 20% denaturing urea-PAGE.

[a]The concentrations used are listed in Table 1 of the main text. [b]Shorter time intervals were applied in time course experiments (Figure S4). [c]0 minutes (Figure S12, Figure S16).

Procedures of template-directed ligation

Template-directed ligation of P1 and L1

P1, T1 (5′-GUCGCUCGAAAGCGACAC-3′), and HEPES were pre-mixed before the addition of L1, *N*-methylimidazole, acetaldehyde, and methyl isocyanide, resulting in a reaction mixture containing P1 (1 μM), L1 (2 μM), template T1 (2 μM), *N*-methylimidazole (200 mM), HEPES (200 mM), acetaldehyde (200 mM), and methyl isocyanide (200 mM). The mixture was incubated at room temperature (18 °C) in a well-ventilated fume hood for 24 hours. To quench the reaction, 1 μL of the mixture was added to 9 μL of quenching buffer containing 84% formamide, 50 mM EDTA (pH 8.0), and 20 μM c-T1 RNA (5′-GUGUCGCUUUCGAGCGAC-3′). The quenched sample was heated at 90 °C for 1 minute and then slowly cooled to 23 °C at a rate of 0.1 °C/s. The resulting mixture was analyzed by 20% denaturing urea-PAGE.

Template-directed ligation of P3 and L1

(1) Ligation reaction and product analysis:

P3, T2 (5′-AAAAGUCGCUCGAACCAUCUGGAAAA-3′), and HEPES were pre-mixed before the addition of L1, *N*-methylimidazole, acetaldehyde, and methyl isocyanide. The final reaction mixture contained P3 (1 μM), L1 (2 μM), template T2 (2 μM), *N*-methylimidazole (200 mM), HEPES (200 mM), acetaldehyde (200 mM), and methyl isocyanide (200 mM). The reaction was incubated at room temperature (18 °C) for 24 hours in a well-ventilated fume hood. To quench the reaction, 1 μL of the mixture was added to 9 μL of quenching buffer containing 88% formamide, 50 mM EDTA (pH 8.0), and 20 μM c-T2 (5′-UUUUCCAGAUGGUUCGAGCGACUUUU-3′). The quenched sample was heated at 90 °C for 1 minute and then slowly cooled to 23 °C at a rate of 0.1 °C/s. The resulting mixture was analyzed by 20% denaturing urea-PAGE.

(2) Scaled-up ligation and product purification:

To determine the ratio of 2′-5′ to 3′-5′ linkages at the ligation site, a scaled-up reaction (200 μL total volume) was performed, followed by Exonuclease T digestion. After the 31-hour ligation, the mixture was snap-cooled on dry ice and placed under vacuum to remove volatile components (H_2O, methyl isocyanide, and acetaldehyde). To the remaining residue, 10 μL GlycoBlue™ Coprecipitant

(15 mg/mL, AM9516, Invitrogen™), 10 µL 5 M NaOAc, and 1 mL ethanol were added. The mixture was cooled on dry ice for 30 minutes, then centrifuged at 21,130 rcf for 10 minutes. The supernatant was carefully removed, and the pellet was washed twice with 1 mL of cold 80% ethanol. The washed pellet was resuspended in 50 µL of 90% formamide containing 50 mM EDTA (pH 8.0), and 10 µL of c-T2 (800 µM) was added. The mixture was heated at 90 °C for 3 minutes and slowly cooled to 21 °C at 0.1 °C/s. The resulting sample was purified by 20% denaturing urea-PAGE to isolate the ligation product. The product was extracted from the gel, desalted using Waters Sep-Pak C18 cartridges, and concentrated by lyophilization at -20 °C. The resulting product was dissolved in 15 µL of nuclease-free water. The concentration of the purified oligonucleotide was determined by fluorescence intensity using HPLC, with BODIPY-labeled H2 as a standard.

(3) Exonuclease T Digestion:

BODIPY-labeled H2-3′o, H2-2′o, an H2-3′o/H2-2′o mixture, or the purified ligation product was mixed with NEBuffer™ 4 (B7004S, New England Biolabs) at 21 °C. Exonuclease T (M0265L, New England Biolabs) was added and gently mixed to yield a reaction mixture containing 1 µM BODIPY-labeled oligoribonucleotide, 1× NEBuffer™ 4, and 10 units of Exonuclease T. The mixture was incubated at 21 °C. At each time point, 1 µL of the reaction mixture was added to 9 µL of quenching buffer containing 90% formamide and 50 mM EDTA (pH 8.0), followed by analysis using 20% denaturing urea-PAGE.

1× Buffer Components:

50 mM Potassium Acetate

20 mM Tris-acetate

10 mM Magnesium Acetate

1 mM DTT

pH 7.9@25°C

General operation of loop-closing ligation by pre-activation strategy

Step 1:

A 10 μL mixture containing L1 (100 μM), imidazole (100 mM), *N*-(3-Dimethylaminopropyl)-*N'*-ethylcarbodiimide hydrochloride (80 mM) was incubated at room temperature for 2 hours. Then 300 μL cold $NaClO_4$ (50 mM in acetone) was added, vortexed, and centrifuged at 21,130 rcf for 3 minutes at 2 °C. The supernatant was decanted. The resulting pellet was washed twice using 300 μL cold $NaClO_4$ (50 mM in acetone) and quickly dried by gentle N_2 flow. The final pellet was dissolved in 100 μL H_2O, and the concentration of Im-p-L1 was confirmed by UV absorbance at 260 nm using a NanoDrop spectrophotometer.

Step 2:

A 20 μL mixture containing primer (P1 or P2, 1 μM), NaCl (100 mM), additives (50 mM $MgCl_2$ and/or 200 mM *N*-methylimidazole if added, see Figure S25), and Im-p-L1 (1 μM) was incubated at 18 °C (note: the Im-p-L1 was added last). 1 μL of the reaction mixture was quenched in 9 μL buffer containing 90% formamide and 50 mM EDTA (pH 8.0) at certain time points. The quenched solution was then analyzed by 20% denaturing urea-PAGE.

Synthesis of *N*-imidoyl-*N'*-methylimidazolium (IMI) 11

Step 1: Reaction

800 μL H_2O, 800 μL 1,2-dimethylimidazole (1 M, pH 8.0), 800 μL acetaldehyde (1 M), and 1.6 mL methyl isocyanide (0.5 M) were mixed, resulting in a solution (4 mL in total) containing 200 mM 1,2-dimethylimidazole, 200 mM acetaldehyde, and 200 mM methyl isocyanide. The reaction mixture was incubated at room temperature (18 °C) for 5 hours.

*Step 2: Preparation of the stock solution of IMI **11***

After incubation at 18 °C for 5 hours, residual acetaldehyde, isocyanide, and water were removed by lyophilization at 0 °C. The resulting product was suspended in 800 μL H_2O, resulting in a stock solution of IMI **11**.

Step 3: Concentration calibration of the stock solution of IMI ***11***

A 50 μL aliquot of the stock solution was mixed with 400 μL D_2O and 50 μL 1M 2,6-lutidine (reference for calibration) in an NMR tube for IMI **11** concentration calibration by ^{1}H NMR. The stock solution contained IMI **11** (*cis* and *trans*) and 1,2-dimethylimidazole. The concentration of **11** (*cis* plus *trans*) was calculated as 0.3 M. This stock solution was used for AMP activation and loop-closing ligation.

Note: To improve resolution, the stock solution was buffered with triethylammonium bicarbonate (pH 8.5) for further ^{1}H NMR, ^{13}C NMR, ^{1}H-^{13}C HMBC, ^{1}H-^{13}C HSQC, and ^{1}H-^{1}H COSY characterization.

AMP activation by *N*-imidoyl-*N'*-methylimidazolium (IMI) 11

25 μL AMP (0.5 M, pH 8.0), 100 μL *N*-methylimidazole (1 M), 75 μL H_2O, 50 μL D_2O, and 250 μL IMI **11** solution (100 mM) were mixed, resulting in final concentration of 25 mM AMP, 200 mM *N*-methylimidazole, and 50 mM IMI **11**. The mixture was incubated at room temperature (18 °C), and a ^{31}P NMR spectrum was taken after 2 hours.

Loop-closing ligation employing *N*-imidoyl-*N'*-methylimidazolium (IMI) 11

A reaction mixture containing 1 μM P1, 2 μM L1, 200 mM *N*-methylimidazole (pH 8.0), and 20 mM IMI **11** was incubated at 20 °C in a well-ventilated fume hood. At 24 h, 48 h, and 72 h, 1 μL aliquots were taken, mixed with 9 μL of 90% formamide (50 mM EDTA, pH 8.0), and heated at 95 °C for 3 min before analysis by 20% denaturing urea-PAGE.

Assembly of Flexizyme by *in situ* loop-closing ligations

Step1: 10 μL **F1** (10 μM), 10 μL **F2** (20 μM), 20 μL **F3** (40 μM), 10 μL *N*-methylimidazole (2 M, pH 8.0), 20 μL NaCl (1 M), and 10 μL H_2O were mixed first. Then 10 μL acetaldehyde (0.5 M) and 10 μL methyl isocyanide (0.5 M) were added to the mixture in a well-ventilated fume hood. The subsequent mixture was incubated at room temperature.

Step 2: After 1 day, an additional 10 μL acetaldehyde (0.5 M) and 10 μL methyl isocyanide (0.5 M) were added to reaction mixture.

Step 3: After 2 days, 20 μL glycogen was added to the resulting solution. The mixture was transferred to a 1.5 mL tube, followed by the addition of 400 μL cold absolute ethanol and 40 μL sodium acetate (3 M, pH 5.5). The resulting mixture was stored at −80 °C freezer for 30 minutes, centrifuged at 21,130 rcf for 10 minutes, and the supernatant was carefully decanted. The pellet was washed twice with 80 μL cold 75% ethanol.

Step 4: The final precipitate was dissolved in 100 μL 90% formamide (50 mM EDTA, pH 8.0), and the sample was divided into two aliquots. The first aliquot was directly subjected to PAGE purification to obtain **dFx-mut-a**. The second aliquot was heated at 95 °C for 1 hour before PAGE purification to obtain **dFx-mut-a***.

Activity evaluation of Flexizymes

A 10 μL solution containing 0.5 μM 8-mer substrate (5′-FAM-AGAAGCCA-3′), 0.5 μM Flexizyme, 100 mM HEPES (pH 8.0), 100 mM $MgCl_2$, and 5 mM dinitrobenzyl ester of glycine (DBE-gly) was incubated at 0 °C. Aliquots of 1 μL were taken at 0.5 h, 1 h, 2 h, 3 h, and 4 h, quenched with 9 μL of acidic quench buffer, and analyzed by acidic 20% denaturing urea-PAGE. The gel was run in an acidic running buffer (100 mM sodium acetate) at 4 ºC and visualized with a Typhoon 9410 imager. The gel image is shown in Figure S24.

Note: (1) The DBE-gly was added as solution in DMSO, so the reaction mixture consisted of 2 μL DMSO and 8 μL H_2O. (2) Acidic quenching buffer: 10 mM EDTA (pH 8.0), 100 mM sodium acetate (pH 5.0), 150 mM HCl, 75% (v/v) formamide in H_2O. (3) The acidic 20% polyacrylamide gel was made with 100 mM sodium acetate (pH 5.0), instead of the usual 1x Tris-Borate-EDTA.

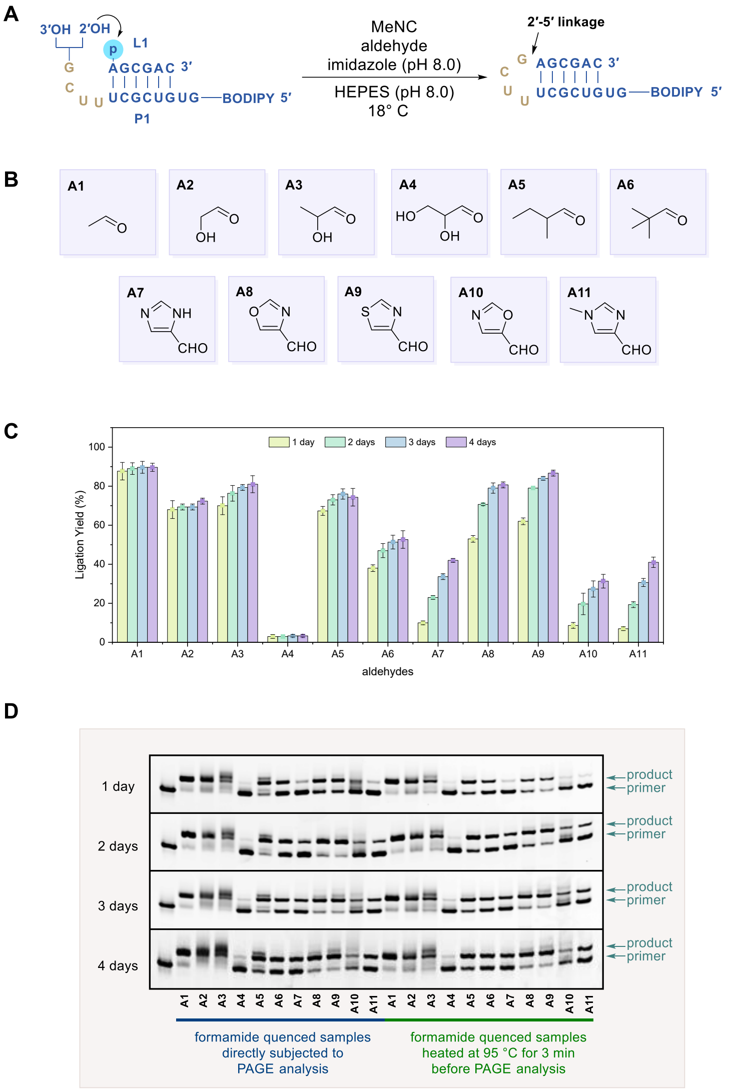

Figure S1. Loop-closing ligation by *in situ* phosphate activation. (**A**) The one-pot *in situ* loop-closing ligation using various aldehydes. Reactions were conducted with a 12-nt primer (1 µM), 6-nt ligator (2 µM), *N*-methylimidazole (200 mM, pH 8.0), HEPES (200 mM, pH 8.0), aldehyde (200 mM), MeNC (200 mM) at 18°C. (**B**) Chemical structures of aldehydes. (**C**) Ligation yields obtained with different aldehydes over four days. **(D)** Gel image of the reactions. The upper bands correspond to the loop-closing ligation product, while the lower bands represent the dye-labeled primer. Additional bands, attributed to base modification, were observed but could be removed upon heating. More complex aldehydes, including 2-methylbutyraldehyde (**A5**), pivaldehyde (**A6**), and aromatic aldehydes (**A7**–**A11**), were also tested to compare reactivity, despite the lack of elucidated prebiotic synthetic routes for these compounds. Notably, nitrogen-containing aromatic aldehydes (**A7**–**A11**) functioned as co-activating agents, though they required longer activation times. These results highlight the remarkable flexibility in the selection of aldehyde partners, demonstrating that this activation chemistry is broadly applicable regardless of which specific aldehydes were prebiotically available. Error bars represent standard deviations from the mean, n = 3 replicates.

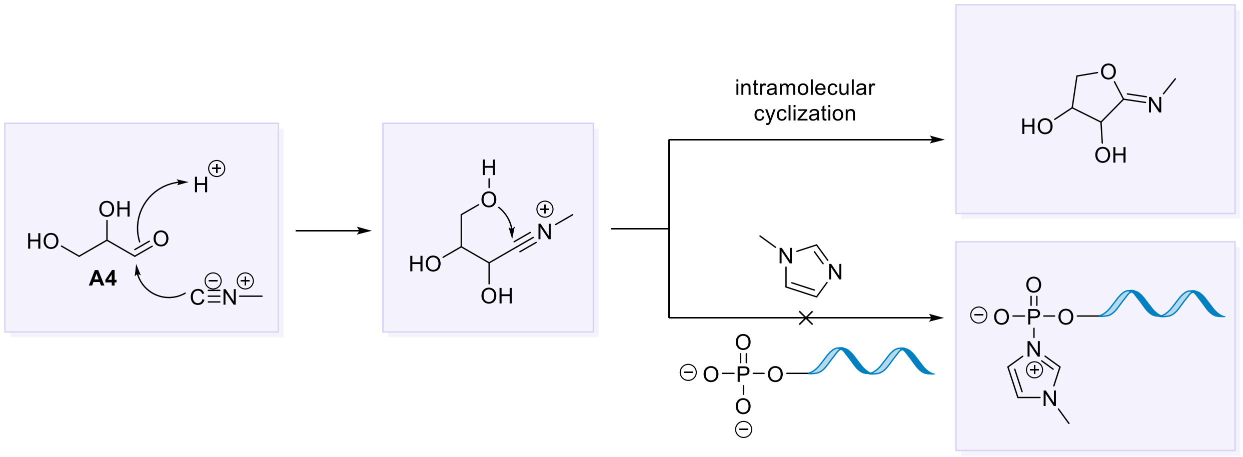

Figure S2. Plausible depletion of nitrilium ion via intramolecular nucleophilic attack. When glyceraldehyde is attacked by isocyanide, the resulting nitrilium ion may preferentially undergo intramolecular cyclization, preventing further activation of the phosphate group.

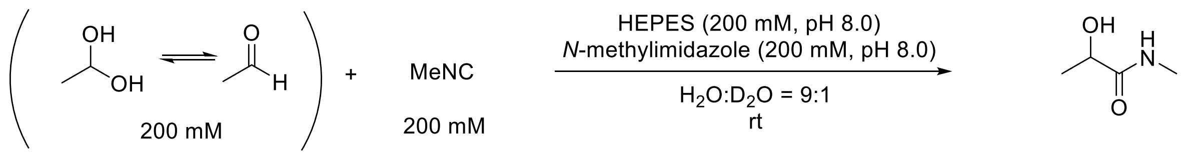

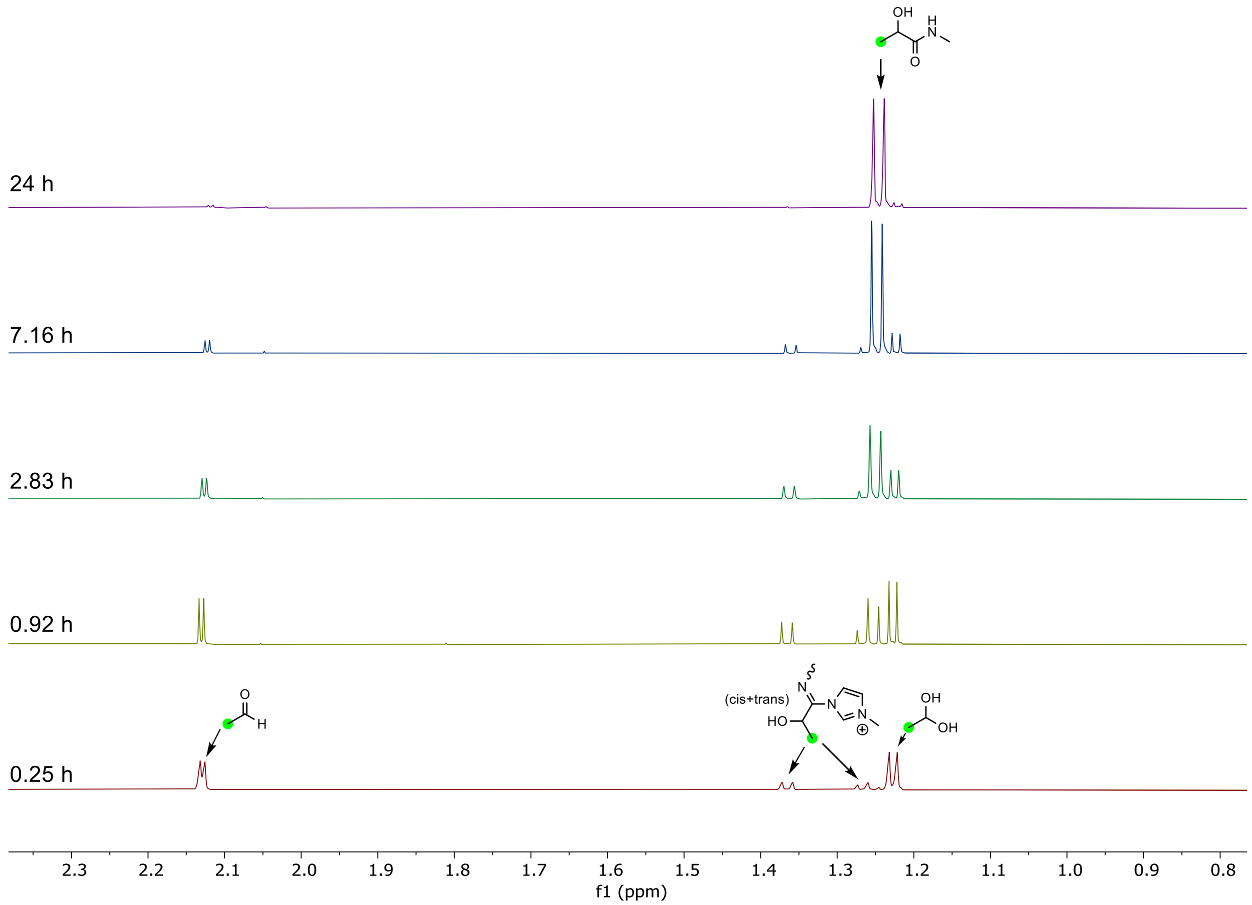

Figure S3. Acetaldehyde consumption over time. Most of the acetaldehyde is consumed after 24 hours in the presence of HEPES and *N*-methylimidazole.

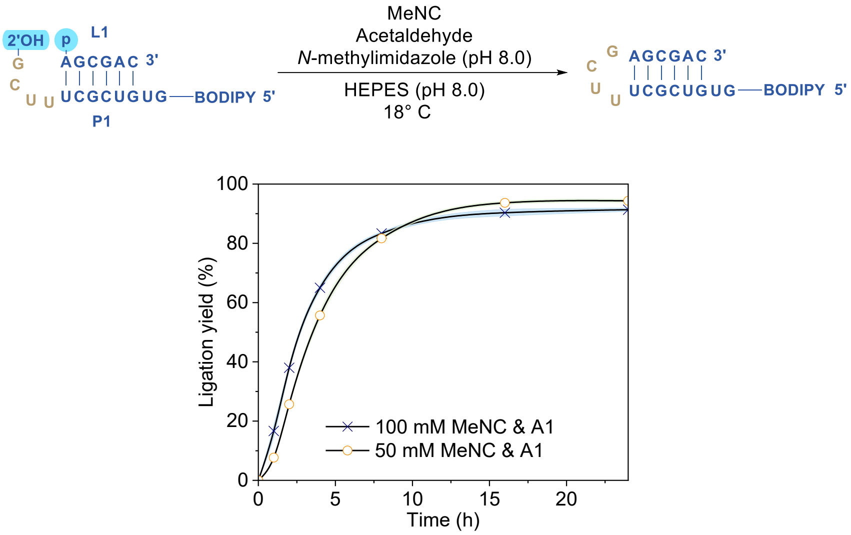

Figure S4. Time course of *in situ* loop-closing ligation. As shown in the figure, the reactions are nearly complete within 12 hours, with only a marginal increase when extending the reaction to 24 hours. The ligation yield is comparable at both 50 mM and 100 mM of activating agents (acetaldehyde and methyl isocyanide). Reactions were conducted with P1 (1 µM), L1 (2 µM), *N*-methylimidazole (200 mM, pH 8.0), HEPES (200 mM, pH 8.0), acetaldehyde (50 or 100 mM), MeNC (50 or 100 mM) at room temperature (18 °C) for 24 hours.

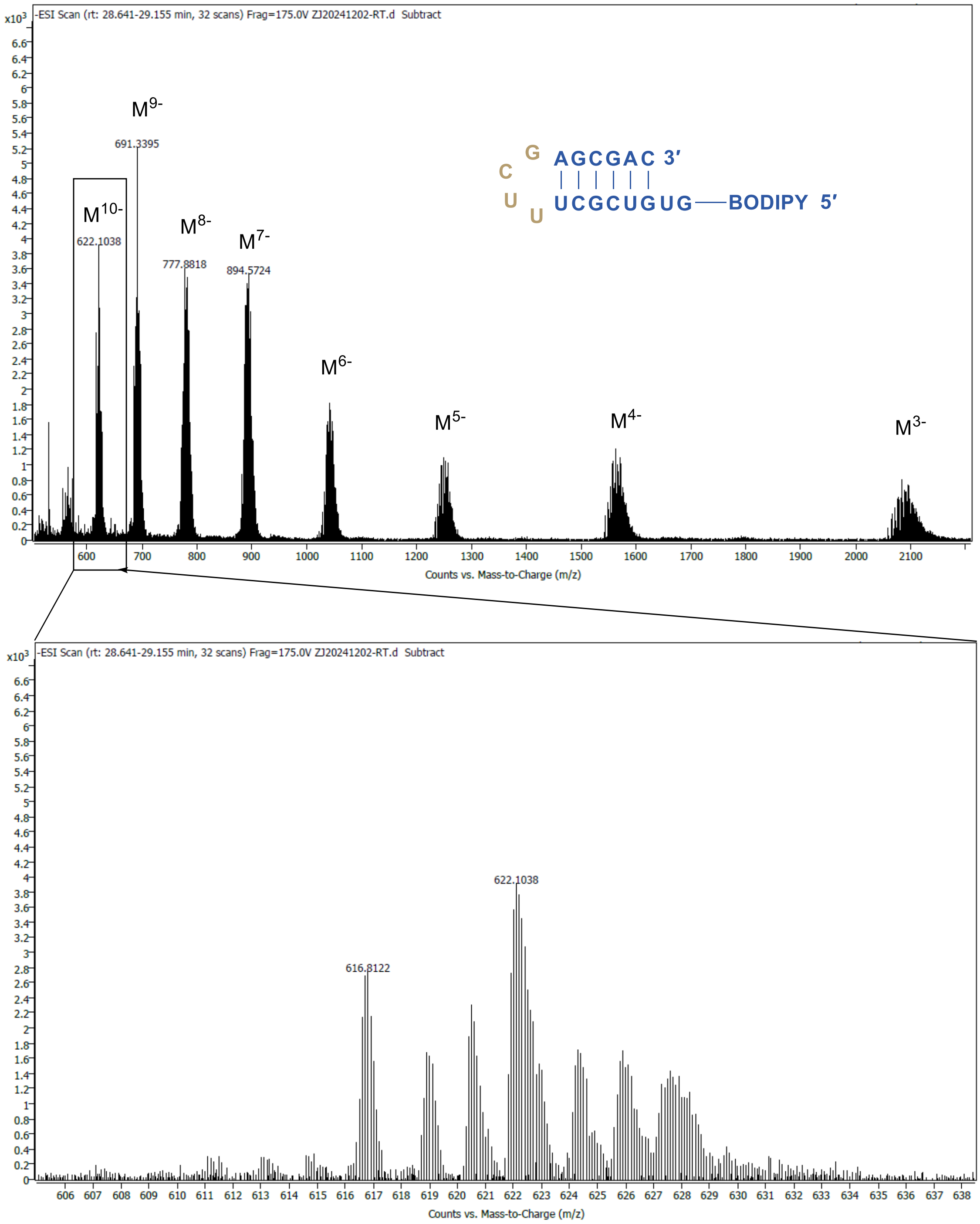

Figure S5. High resolution mass spectra of loop-closing ligation product H1. M^{n-} represents the ionized species bearing differing numbers of negative charges.

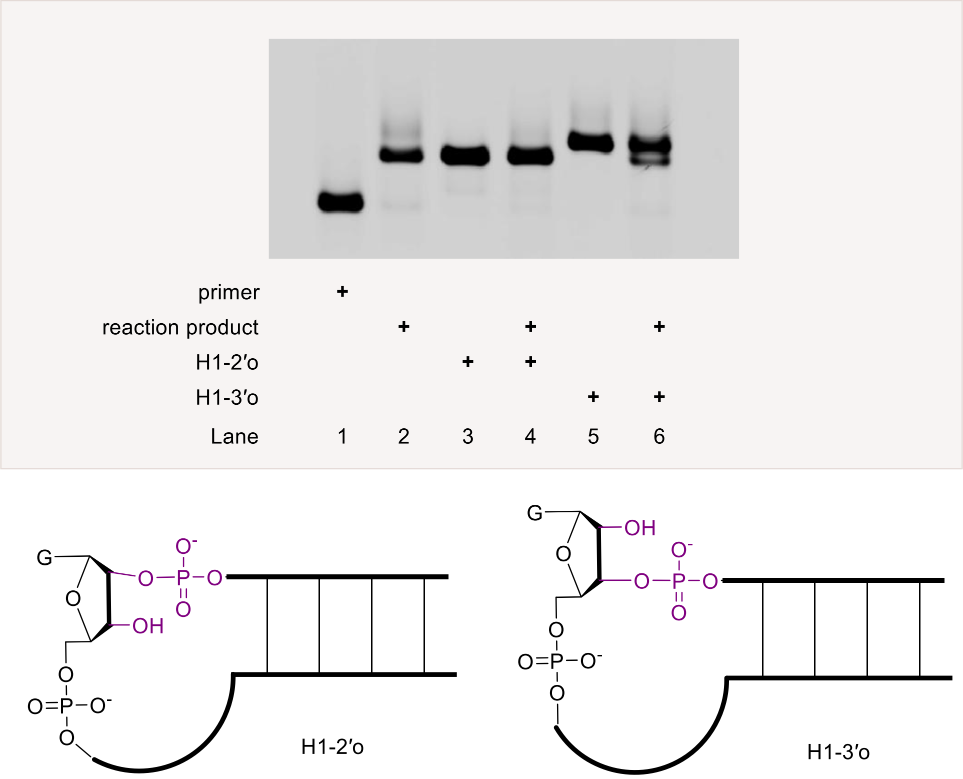

Figure S6. Denaturing PAGE image of *in situ* loop-closing ligation product and authentic samples. The gel image shows the formation of the ligated product in comparison to the authentic samples. The authentic samples, H1-2′o and H1-3′o, were prepared using an RNA synthesizer and contain the identical sequence as the expected ligation product. H1-2′o contains a 2′-5′ phosphodiester linkage at the ligation junction, while H1-3′o contains a 3′-5′ phosphodiester linkage. The PAGE analysis shows that the majority of the product migrates similarly to H1-2′o (Lanes 2-4) but differently from H1-3′o (Lanes 5 and 6). Therefore, the loop-closing ligation regioselectivity favors the 2′-5′ linkage.

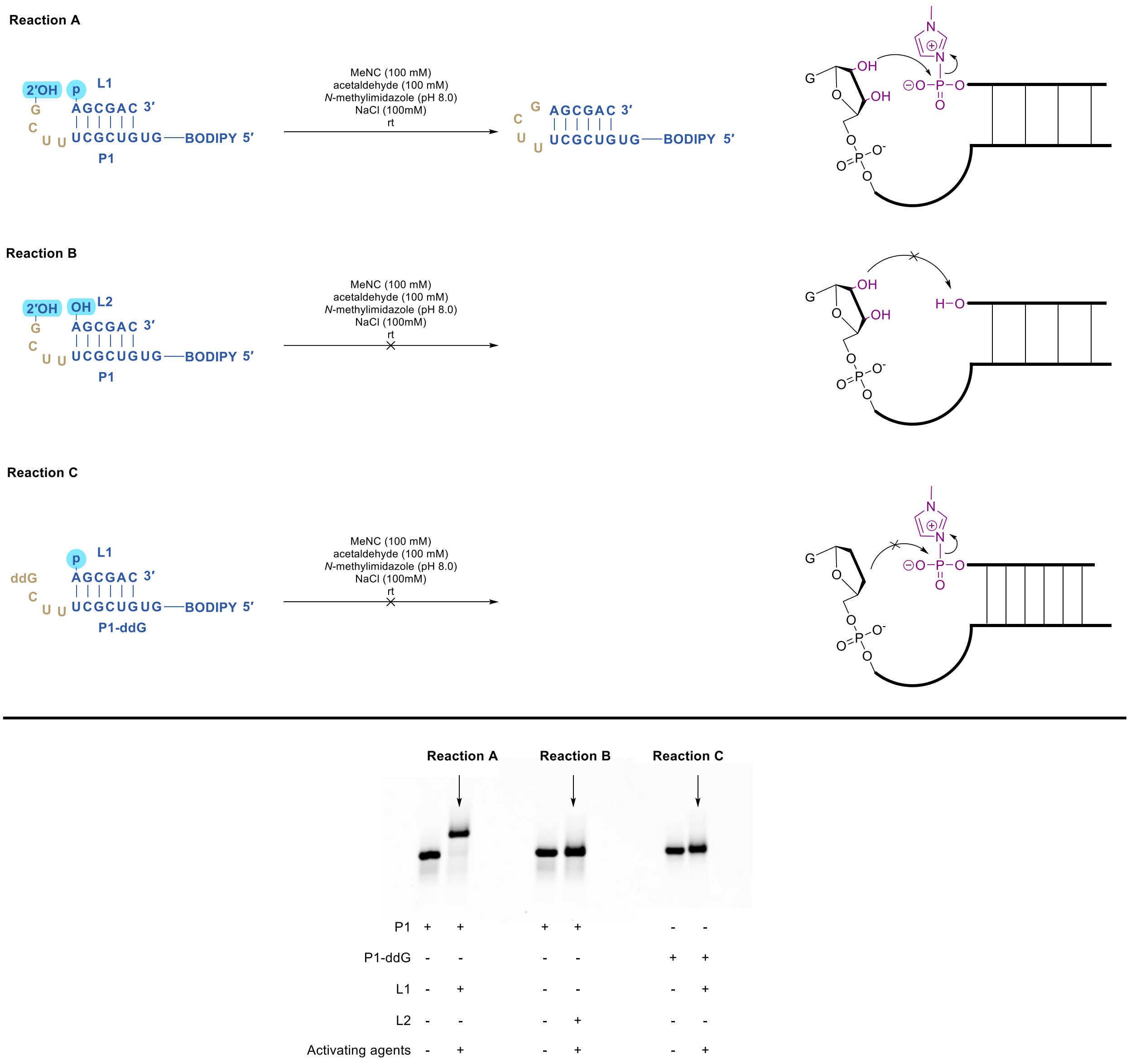

Figure S7. Control experiments to confirm the ligation between the hydroxyl group of the terminal nucleotide of primer P1 and the 5′-phosphate of ligator L1. (Top) Schematic representation of control experiments for loop-closing ligation. **(Bottom)** Denaturing PAGE analysis. No ligated product was observed when a ligator lacking a 5′-phosphate group (reaction B) or a primer terminated with a 2′,3′-dideoxynucleotide (reaction C) was used. Reactions were performed with a 12-nt primer (P1 or P1-ddG, 1 µM), a 6-nt ligator (L1 or L2, 2 µM), *N*-methylimidazole (200 mM, pH 8.0), NaCl (100 mM), acetaldehyde (100 mM), and MeNC (100 mM) at room temperature for 24 hours.

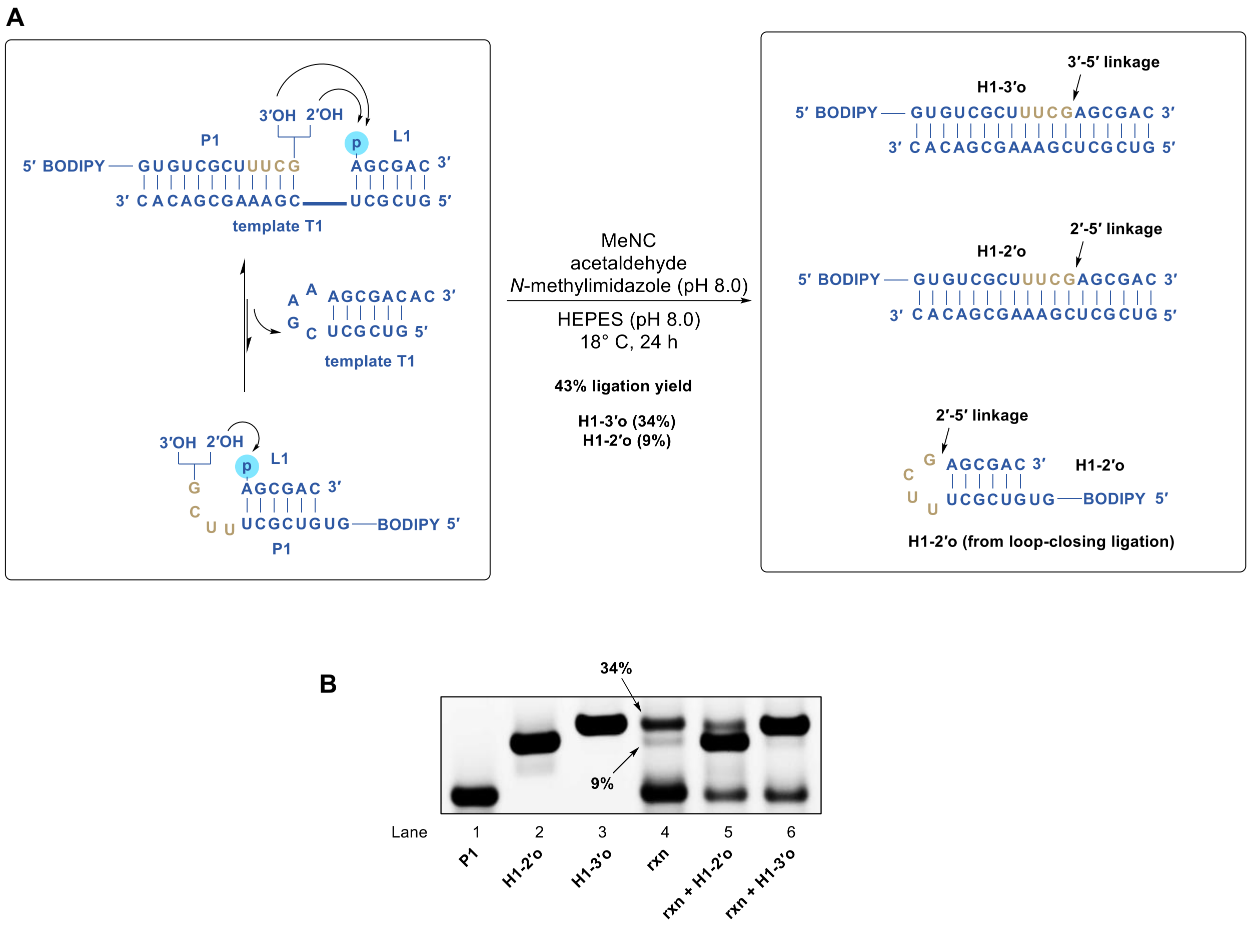

Figure S8. Template-directed ligation. (A) Schematic representation of template-directed ligation and potential competing loop-closing ligation. Reactions were performed with P1 (1 µM), L1 (2 µM), T1 (2 µM), *N*-methylimidazole (200 mM, pH 8.0), HEPES (200 mM, pH 8.0), acetaldehyde (200 mM), and MeNC (200 mM) at 18 °C for 24 hours. In this reaction mixture, both the 3′,5′-linked product (H1-3′o, 34%) and the 2′,5′-linked product (H1-2′o, 9%) were obtained. The total ligation yield was 43%. **(B)** PAGE image of ligation products in the presence of a template. Lane 1 shows the primer (P1). Lane 2 contains the hairpin product with a 2′-5′ linkage (H1-2′o), while lane 3 shows the hairpin product with a 3′-5′ linkage (H1-3′o). Lane 4 presents the reaction mixture in the presence of the template, with bands corresponding to H1-3′o (34%, top band), H1-2′o (9%, middle band), and unreacted P1 (57%, bottom band). Lane 5 shows the reaction mixture spiked with authentic H1-2′o, and lane 6 shows the reaction mixture spiked with authentic H1-3′o.

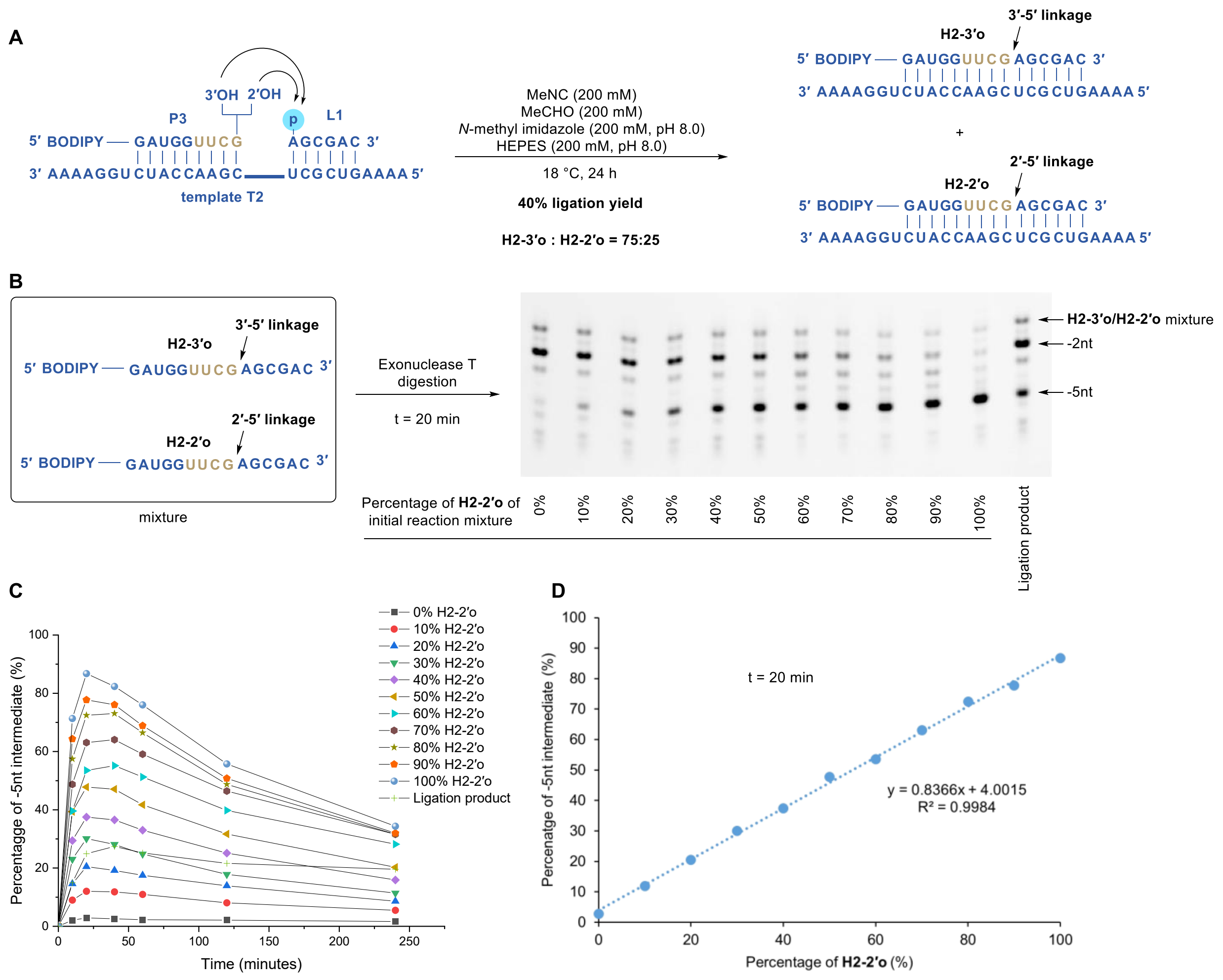

Figure S9. Template-directed ligation in the absence of potential competing loop-closing ligation, and results of Exonuclease T digestion. (A) Schematic representation of template-directed ligation. Reactions were performed using P3 (1 µM), L1 (2 µM), T2 (2 µM), *N*-methylimidazole (200 mM, pH 8.0), HEPES (200 mM, pH 8.0), acetaldehyde (200 mM), and MeNC (200 mM) at 18 °C for 24 hours. Both the 3′,5′-linked product (H2-3′o) and the 2′,5′-linked product (H2-2′o) were generated in this reaction. **(B)** PAGE analysis of Exonuclease T digestion at the 20-minute time point, starting with varying ratios of H2-3′o to H2-2′o and template-directed ligation product. The -2 nt digestion product was confirmed by mass spectrometry (Figure S10) and a synthesized reference. The -5 nt product was confirmed using a synthesized reference. (**C**) Time course showing the percentage of the -5 nt digestion intermediate. Digestion of H2-2′o showed a strong pause at the -5 nt intermediate, leading to its accumulation. In contrast, H2-3′o digestion proceeded without stalling at the -5 nt position, resulting in a lower percentage of this intermediate during the digestion process. The difference in -5 nt intermediate levels between the two linkages was most pronounced at the 20-minute time point. (**D**) The ratio of H2-3′o to H2-2′o (75:25) was determined based on a linear relationship between the percentage of the -5 nt intermediate at 20 minutes and the proportion of H2-2′o in the digestion mixture.

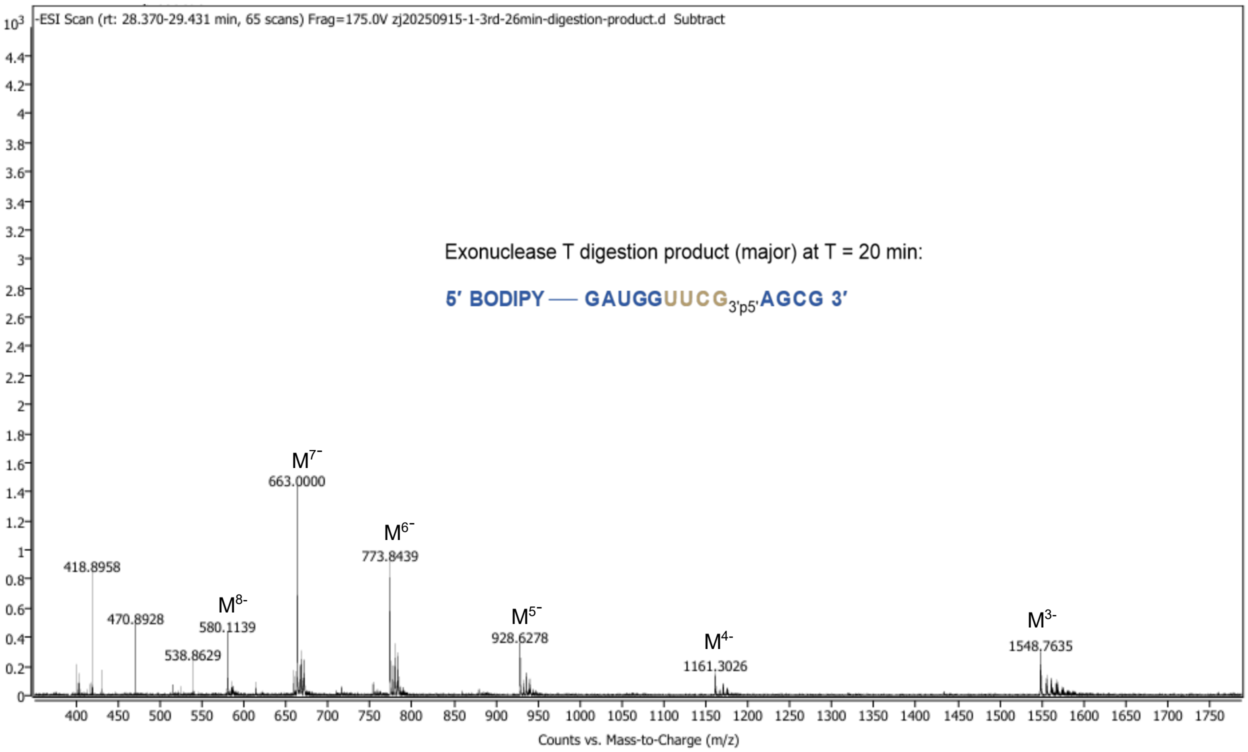

Figure S10. Mass spectrum of major digestion product of H2-3′o at 20-minute time point.

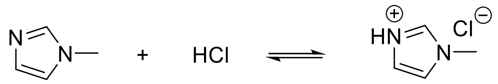

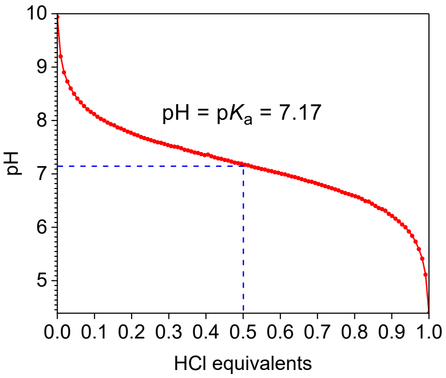

Figure S11. Titration curve of *N*-methylimidazole. The curve was obtained by incrementally adding HCl solution (~0.05 M) to a *N*-methylimidazole solution (0.05 M) and recording the pH value after each addition. According to the Henderson-Hasselbalch equation, the pH (7.17) at the half-equivalence point corresponds to the pK_a of the conjugate acid of *N*-methylimidazole. The pK_a value of 7.17 is consistent with the literature[3,4].

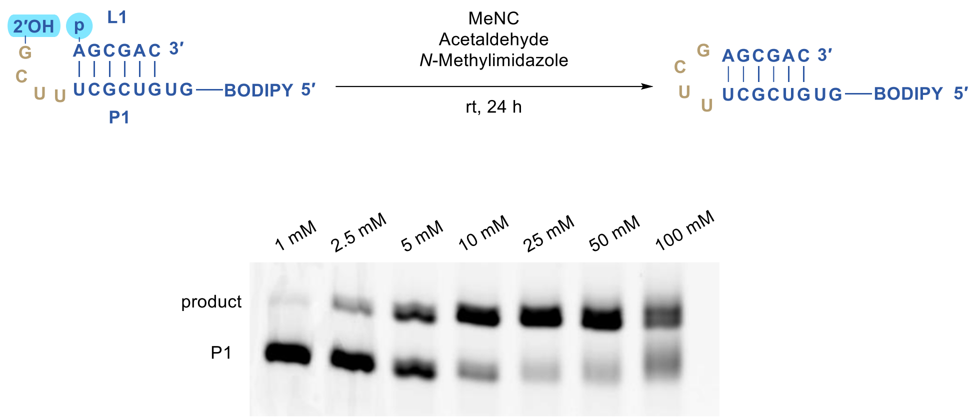

Figure S12. RNA modification by *in situ* activation chemistry revealed by denaturing PAGE. The smearing of the bands indicates potential modification of the oligonucleotides. Reaction conditions: P1 (1 µL), L1 (2 µL), MeNC (1-100 mM), acetaldehyde (1-100 mM), *N*-methylimidazole (200 mM), room temperature, 24 hours. The concentration of MeNC is equal to that of acetaldehyde in each reaction.

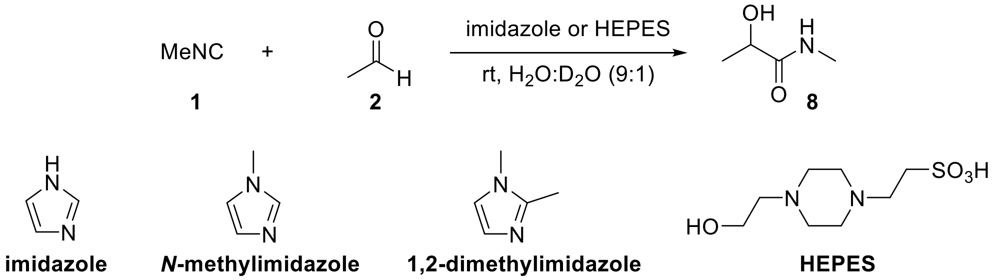

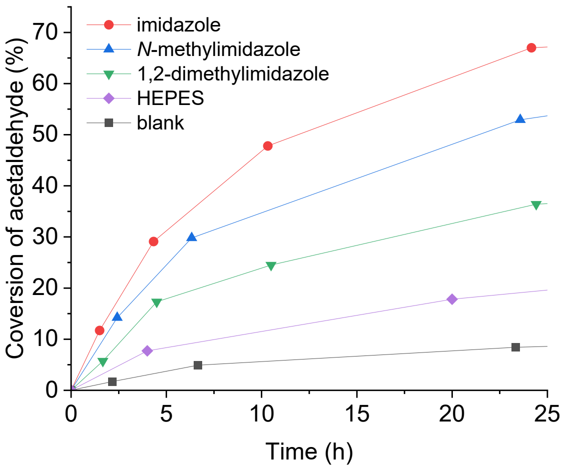

Figure S13. Passerini reaction promoted by imidazoles and HEPES. The reactions were conducted with 25 mM acetaldehyde, 25 mM MeNC, and 200 mM imidazole or HEPES at room temperature in an NMR tube, and monitored using ^{1}H NMR. The ^{1}H NMR recordings were performed on a 500 Hz or 600 Hz instrument to ensure sufficient resolution to differentiate the signals from the product, acetaldehyde, hydrated acetaldehyde, and intermediates. The solutions of imidazoles and HEPES were adjusted to pH 8.0 before use. In the cases of *N*-methylimidazole and 1,2-dimethylimidazole, the conversion rate of acetaldehyde does not represent the rate of product formation because quasi-stable intermediates were formed, with the relative percentage of each component over time shown in Figures S14 and S15.

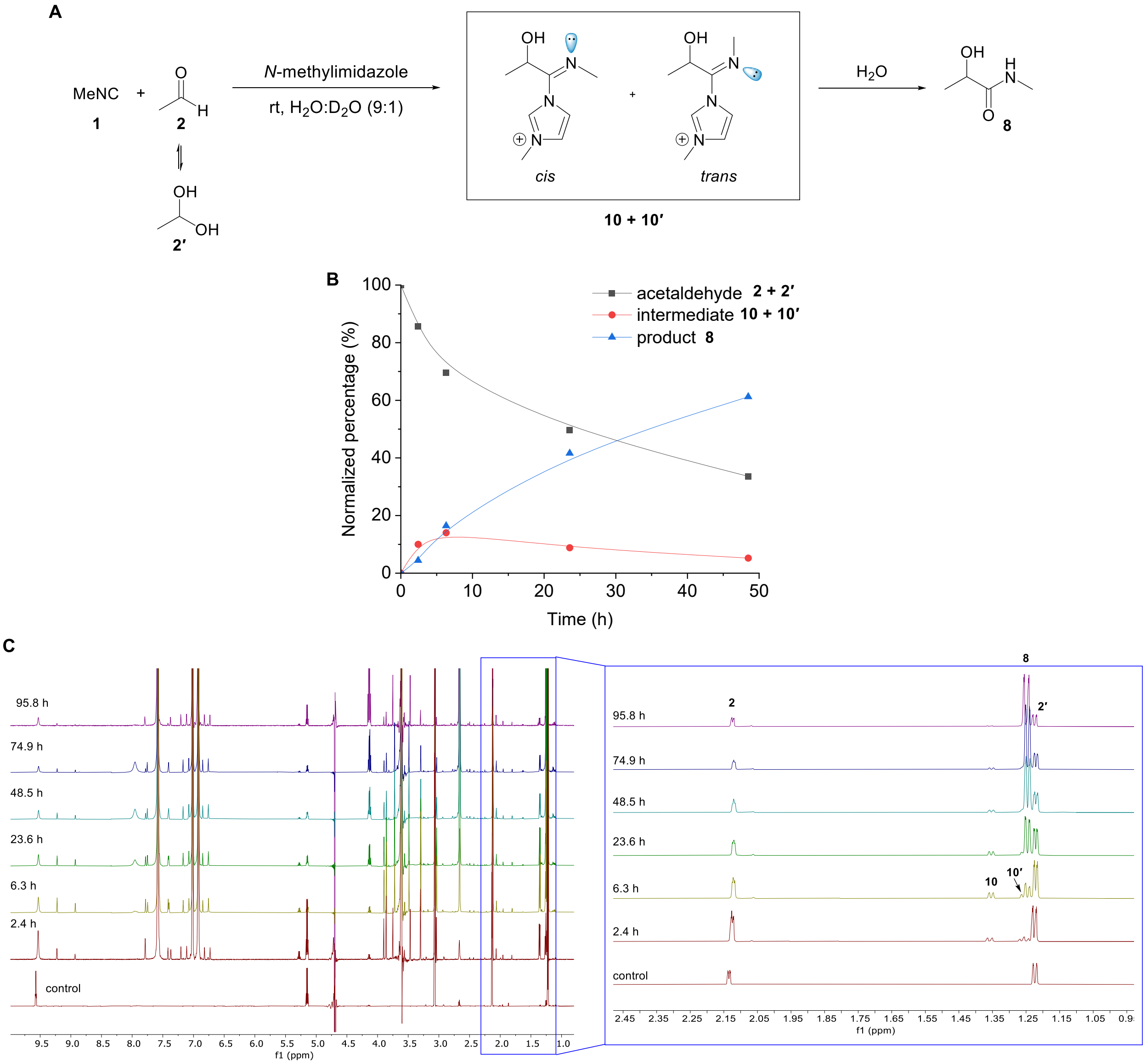

Figure S14. Passerini-type reaction promoted by *N*-methylimidazole. (A) Representation of the reaction of **1** and **2** in the presence of *N*-methylimidazole. The reactions were conducted with 25 mM acetaldehyde, 25 mM MeNC, and 200 mM *N*-methylimidazole at room temperature in an NMR tube and monitored using ^{1}H NMR. **(B)** Plots of the relative percentages of aldehyde **2** (including the hydrated form **2′**), intermediate **10** (including the minor isomer **10′**), and product **8** over time. **(C)** ^{1}H NMR of the reaction mixture of MeNC, acetaldehyde, and *N*-methylimidazole over time. The intermediate **10** (*cis* and *trans*) was produced during the reaction, and decayed over time. The zoomed-in region shows the relative amounts corresponding to the methyl groups on **2**, **2′**, **8**, **10**, and **10′** over time. The ^{1}H NMR recordings were performed on a 500 Hz or 600 Hz instrument to ensure sufficient resolution to differentiate the signals from the product **8**, acetaldehyde **2**, hydrated acetaldehyde **2′**, and the intermediate isomers **10** and **10′**. The solution of *N*-methylimidazole was adjusted to pH 8.0 before use.

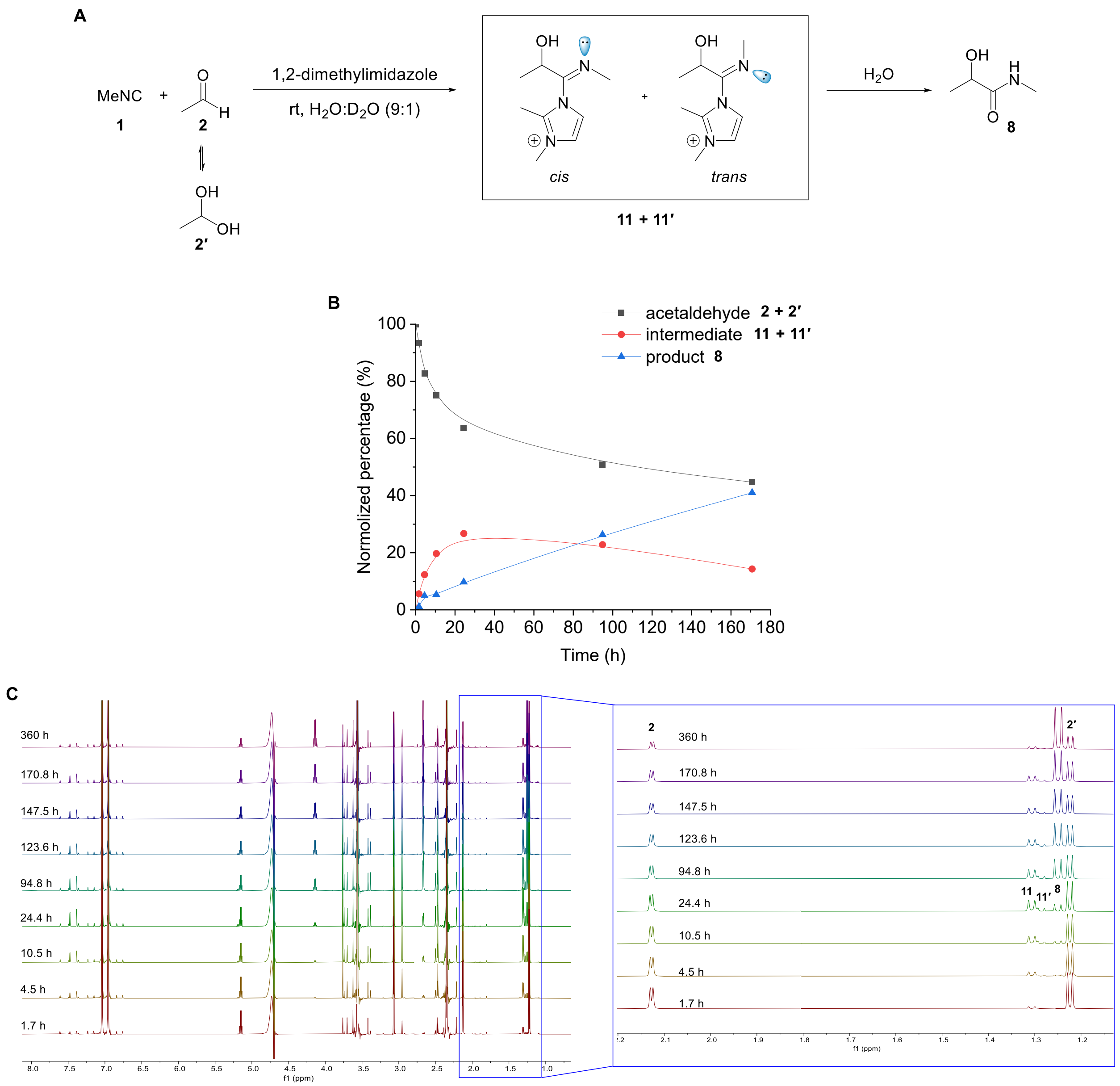

Figure S15. Passerini-type reaction promoted by 1,2-dimethylimidazole. (A) Representation of the reaction of **1** and **2** in the presence of 1,2-dimethylimidazole. The reactions were conducted with 25 mM acetaldehyde, 25 mM MeNC, and 200 mM 1,2-dimethylimidazole at room temperature in an NMR tube and monitored using ^{1}H NMR. **(B)** Plots of the relative percentages of aldehyde **2** (including the hydrated form **2′**), intermediate **11** (including the minor isomer **11′**), and product **8** over time. **(C)** ^{1}H NMR of the reaction mixture of MeNC, acetaldehyde, and 1,2-dimethylimidazole over time. The intermediate **11** (*cis* and *trans*) was produced during the reaction, and decayed over time. The zoomed-in region shows the relative amounts corresponding to the methyl groups on **2**, **2′**, **8**, **11**, and **11′** over time. The ^{1}H NMR recordings were performed on a 500 Hz or 600 Hz instrument to ensure sufficient resolution to differentiate the signals from the product **8**, acetaldehyde **2**, hydrated acetaldehyde **2′**, and the intermediate isomers **11** and **11′**. The solution of 1,2-dimethylimidazole was adjusted to pH 8.0 before use.

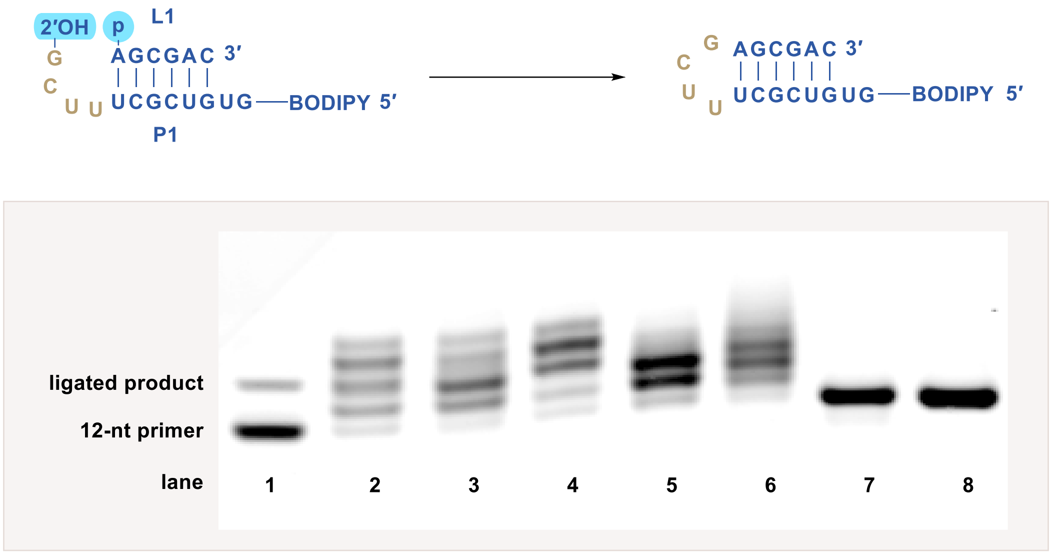

	1	2	3	4	5	6	7	8
HEPES	+*	+	-	+	+	+	+	+
$MgCl_2$	+	+	+	-	+	+	+	+
N-methylimidazole	+	+	+	+	-	+	+	+
6-nt ligator	+#	+	+	+	+	-	+	+
12-nt primer	+	+	+	+	+	+	+	+
MBA	-	+	+	+	+	+	-	+
MeNC	-	+	+	+	+	+	+	-
NaCl	+	-	-	-	-	-	-	-

Figure S16. Denaturing PAGE image showing band shifts caused by base modification. Lane 1 shows the primer and the loop-closing ligation product using a pre-activated ligator. The results in lanes 2, 3, and 4 exclude the possibility that NaCl, HEPES, or $MgCl_2$ promoted the modification. Lane 5 shows the modification of primer in the absence of *N*-methylimidazole. As shown in lane 6, several modified bands of the primer were still observed without the ligator when the primer was treated with 2-methylbutyraldehyde (MBA) and MeNC. No modification was observed in the absence of either MBA or MeNC, as shown in lanes 7 and 8, indicating that the modification resulted from the reaction between the RNA and the nitrilium ion. Reaction conditions: HEPES (200 mM), $MgCl_2$ (50 mM), *N*-methylimidazole (100 mM), 12-nt primer (1 µM), 6-nt ligator (2 µM), MBA (100 mM), MeNC (100 mM), NaCl (100 mM) at 18°C for 26 h. #100 mM HEPES. *The 6-nt ligator was pre-activated by 1-Ethyl-3-(3-dimethylaminopropyl)carbodiimide (EDC) and imidazole.

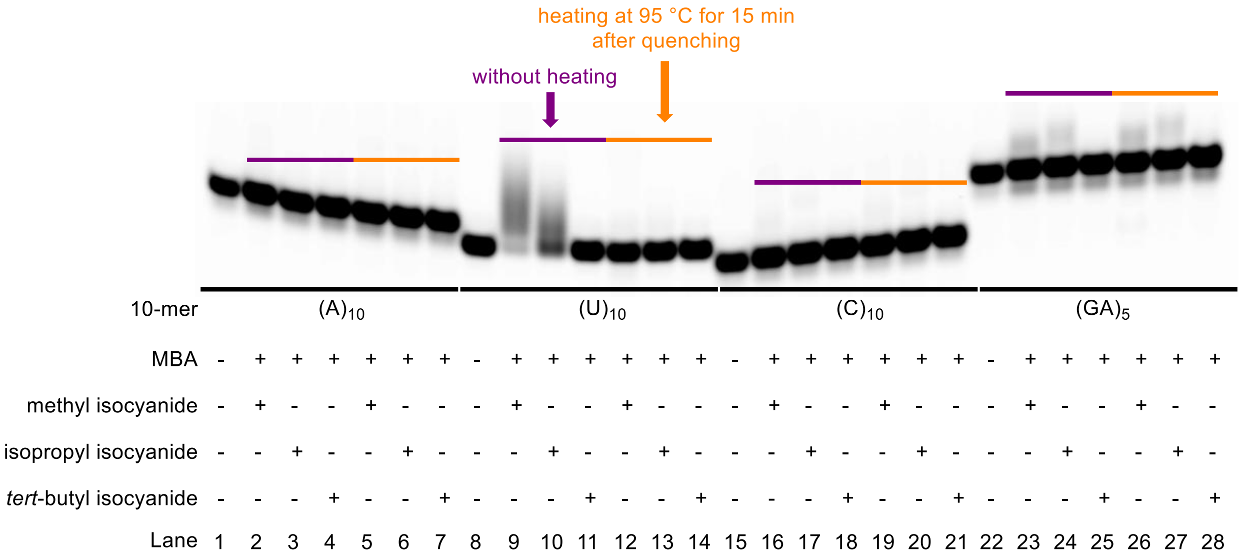

Figure S17. Base modification of oligoribonucleotide 10-mers. Four 10-mers that consist of only one (or two) monomer(s) were examined for modification with the addition of isocyanide and aldehyde. While the oligomer $(U)_{10}$ was modified (lanes 9 and 10) in the presence of methyl- or isopropyl isocyanide, $(A)_{10}$, $(C)_{10}$, $(GA)_5$ remained almost unmodified. This indicates base specific modification, while other functional groups including internal phosphate and 2′-hydroxyl remain intact under activation conditions. $(U)_{10}$ was not modified when *tert*-butyl isocyanide was used probably due to the low concentration or high steric hindrance of corresponding nitrilium ion, emphasizing again that the nitrilium ion is responsible for the modification. The modification is reversible, as unmodified $(U)_{10}$ was recovered after heating (lanes 12 and 13). A 4% modification was detected in lane 23, which was reduced to 1% upon heating (lane 26). Reaction conditions: 10-mer (1µM), HEPES (200 mM, pH 8.0), MBA (200 mM), isocyanide (200 mM), 18°C, 24h.

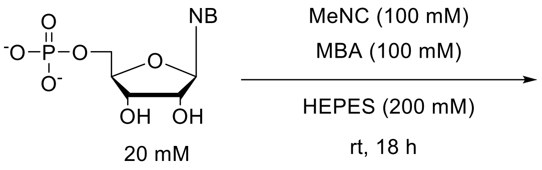

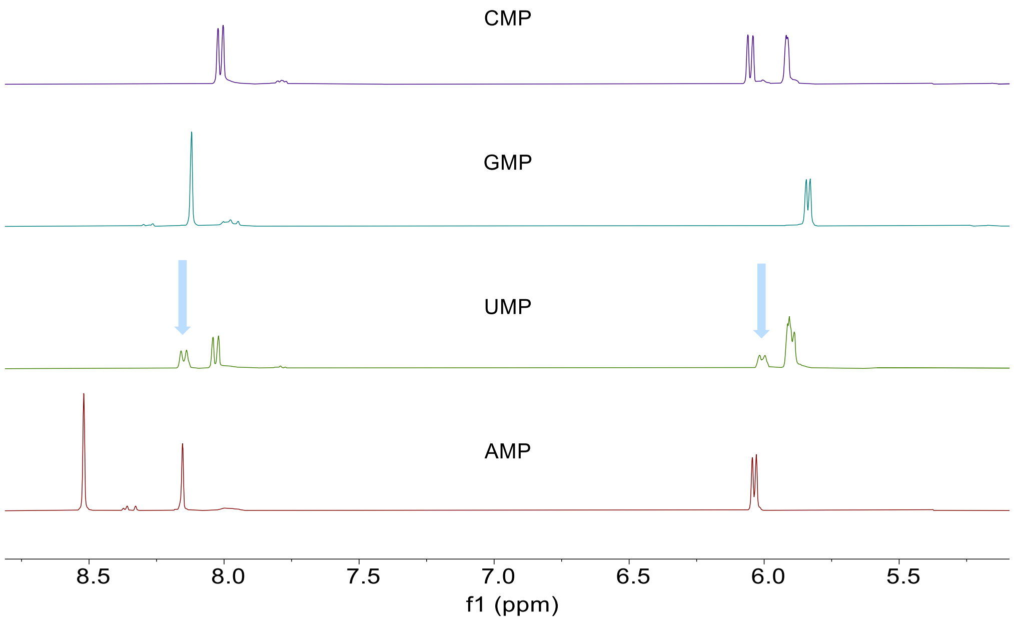

Figure S18. ¹H NMR spectra of ribonucleotides incubated with MeNC, MBA, and HEPES. When the four canonical ribonucleotides were treated with aldehyde and methyl isocyanide, UMP was found to undergo severe modification, as indicated by the ¹H NMR spectra. The ¹H NMR peaks from the modified product are highlighted by arrows.

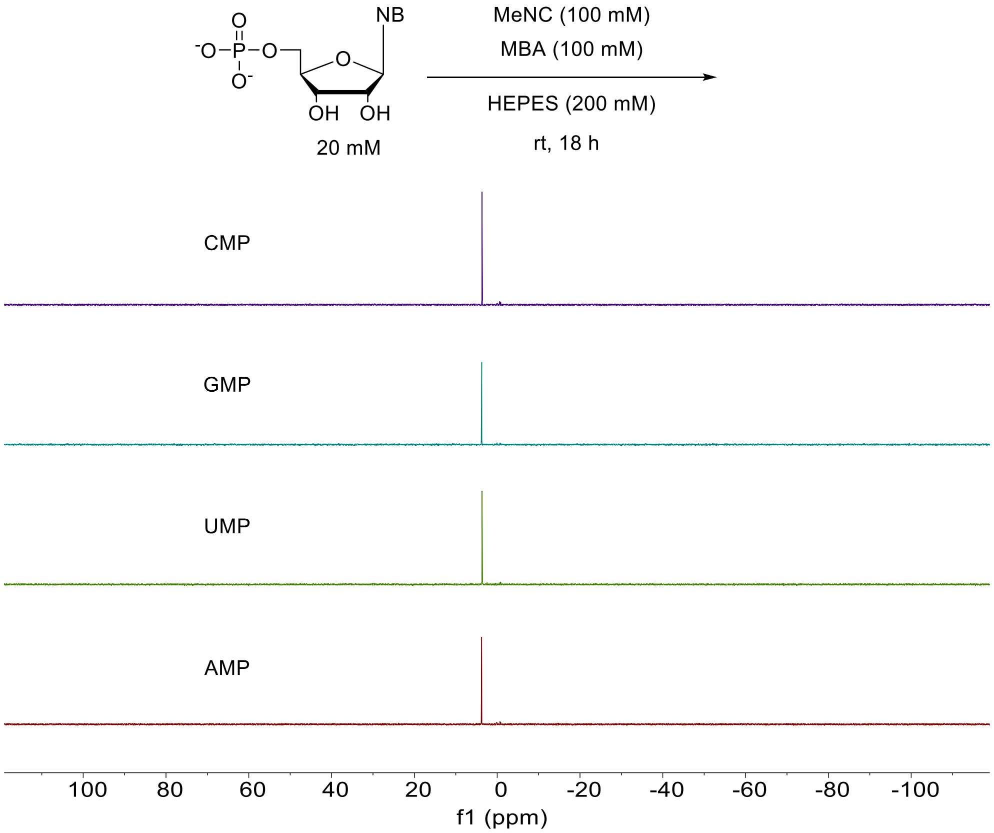

Figure S19. ^{31}P NMR spectra of ribonucleotides incubated with MeNC, MBA, and HEPES. No obvious difference in the ^{31}P NMR signal was observed between all four ribonucleotides after incubation in activating conditions, indicating that the modification is not on the phosphate group.

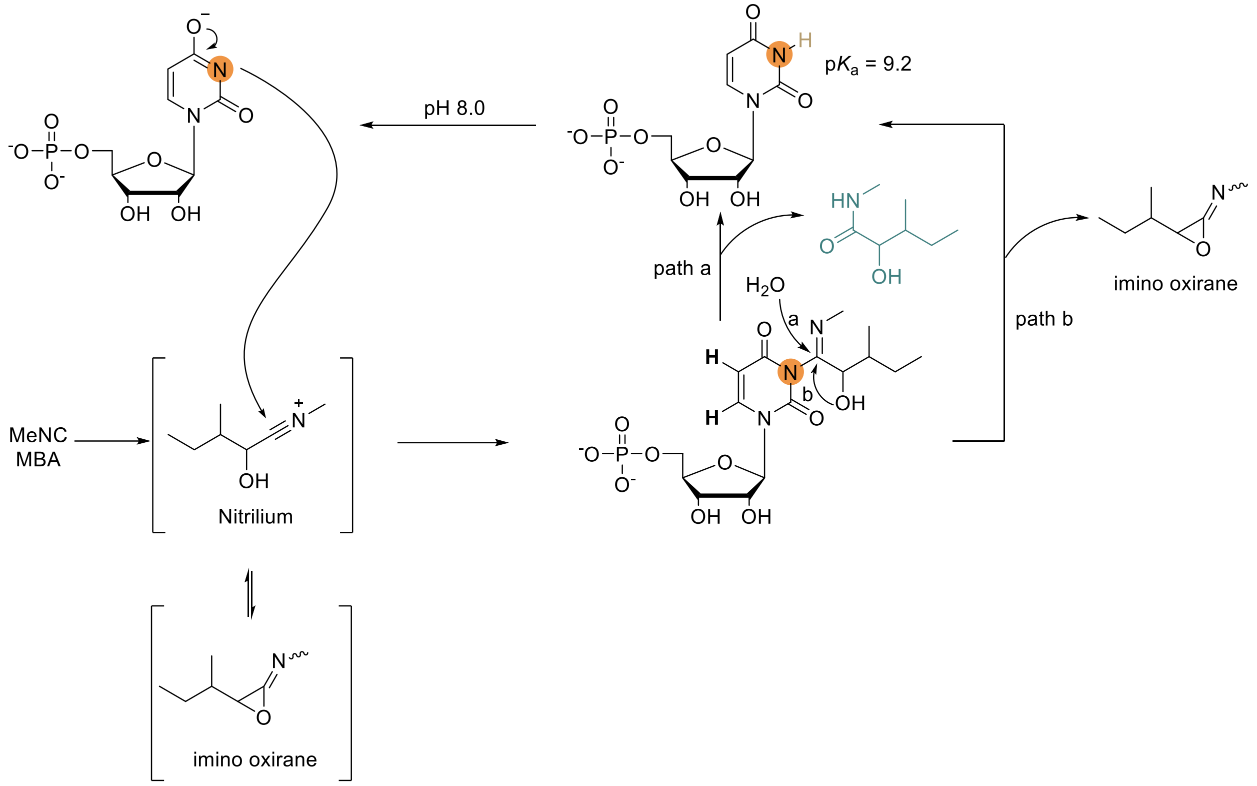

Figure S20. Proposed mechanism of nucleobase (uracil) modification. The pK_a of the nucleobase in uridine monophosphate is 9.2. The highlighted NH readily undergoes deprotonation at pH 8.0, which increases the nucleophilicity of N3 of uracil. Once the deprotonated nitrogen attacks the nitrilium ion, the nucleobase undergoes modification, forming an amidine intermediate. This unstable amidine subsequently undergoes hydrolysis upon heating, releasing uridine monophosphate. Two possible hydrolysis pathways are illustrated in Figure S20. Pathway a involves a direct attack by water, whereas pathway b proceeds via an intramolecular attack by a hydroxyl group, leading to the formation of an imino oxirane—another intermediate that may exist in equilibrium with the nitrilium ion[5].

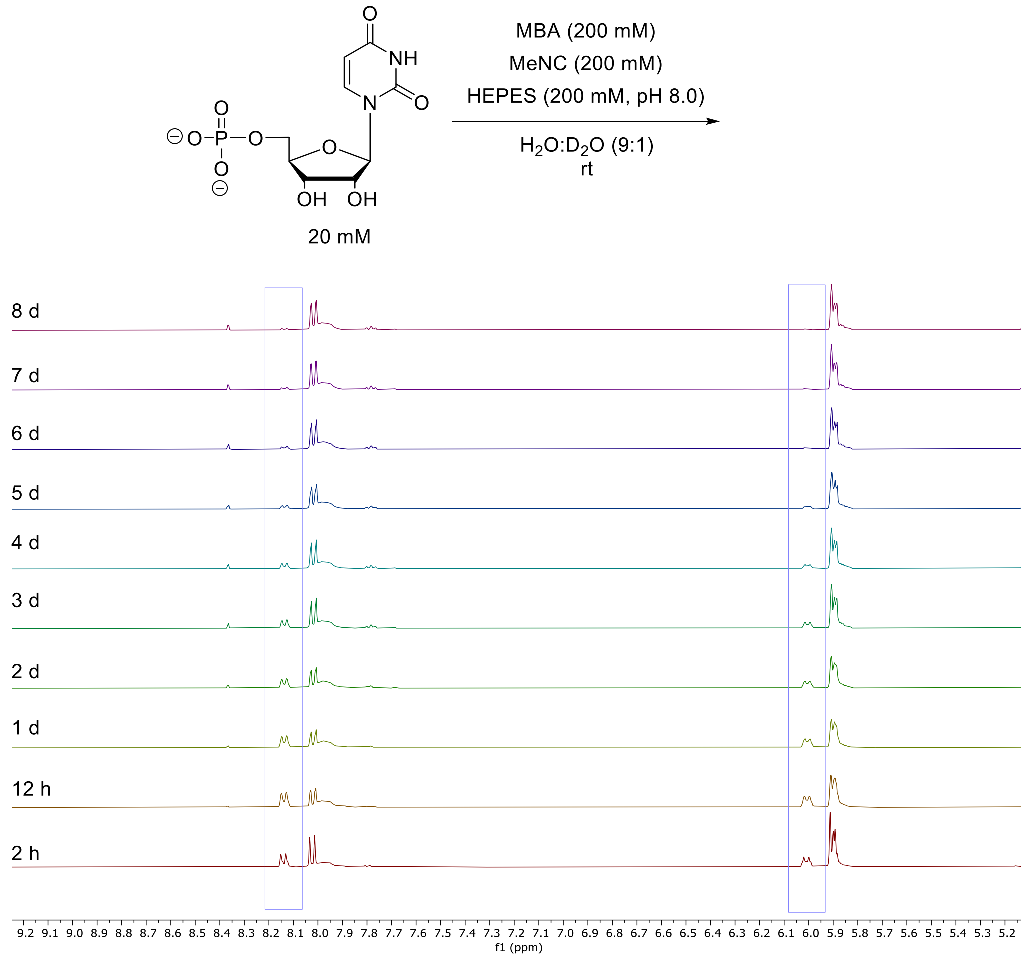

Figure S21. Time course of hydrolysis of the modified UMP product, as monitored by ^{1}H NMR spectra. This region of the spectra shows peaks corresponding to the protons on the uracil base. Boxed regions illustrate peaks arising from uracil modification. The modified product underwent hydrolysis slowly at room temperature over 8 days, returning to unmodified uracil.

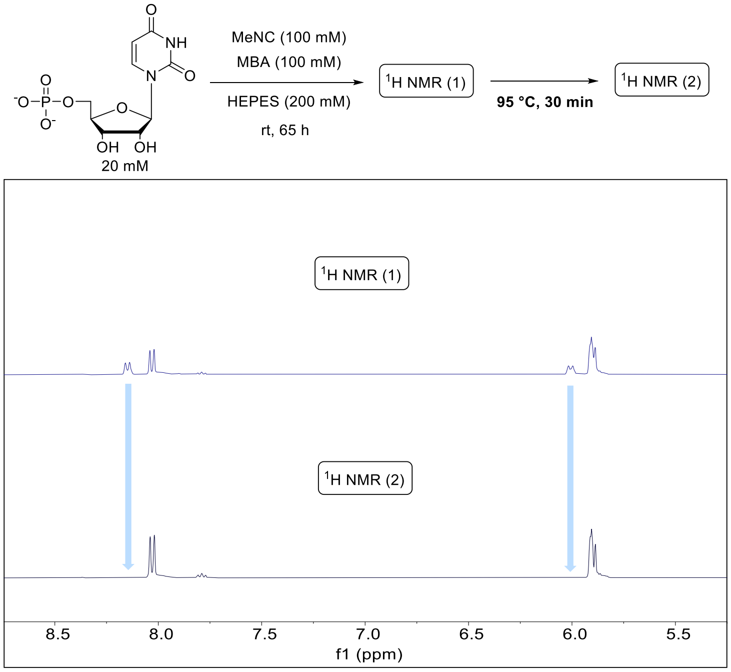

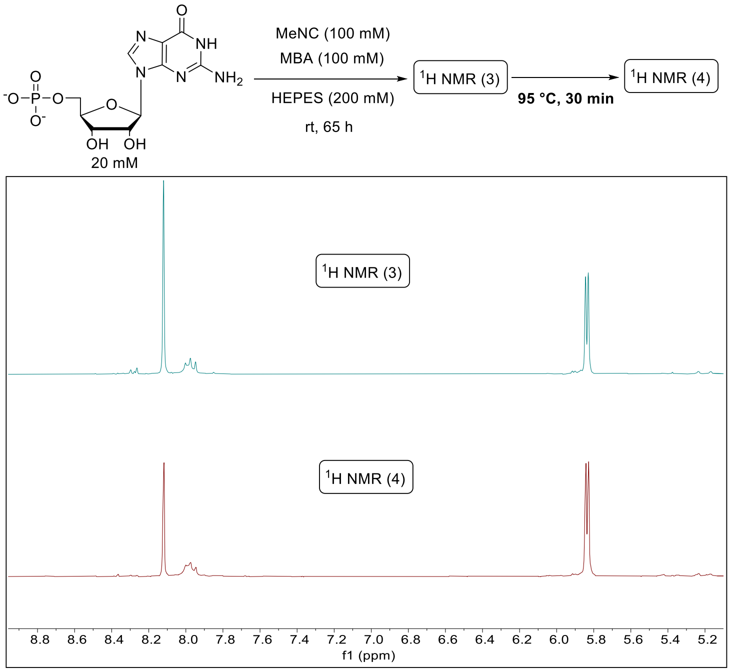

Figure S22. ^{1}H NMR spectra of UMP (top) and GMP (bottom) after incubation with MBA and MeNC for 65 hours and following subsequent heating. Heating accelerates the hydrolysis of the modified mononucleotides, with unmodified UMP recovered after 30 minutes at 95 °C.

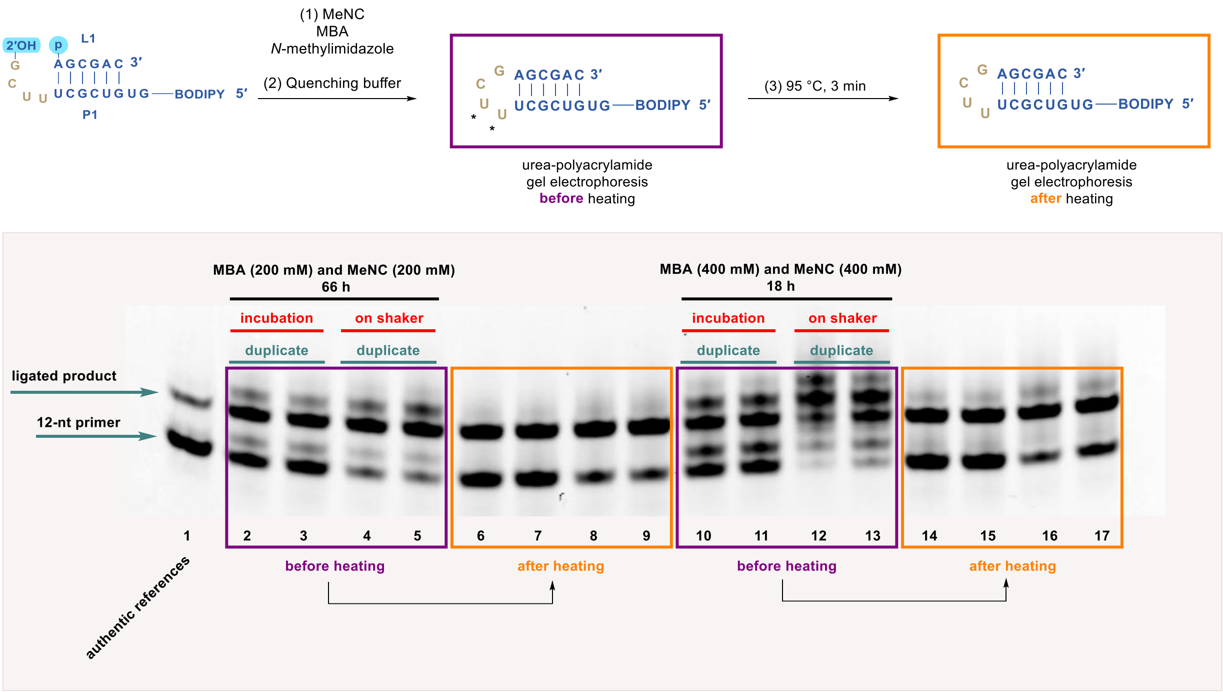

Figure S23. Rescue of modified oligoribonucleotides by heating. Both the primer and ligation product undergo modification in the presence of 2-methylbutyraldehyde (MBA) and methyl isocyanide. Obvious modification is only observed when the samples were analyzed directly by PAGE after quenching. However, if the samples were heated at 95°C for 3 minutes after quenching, most of the modified bands disappeared. Therefore, heating can be considered a rescue method to revert the modification. Reaction conditions: P1 (1 μM), L1(2 μM), MBA (200 or 400 mM), MeNC (200 mM or 400 mM), room temperature.

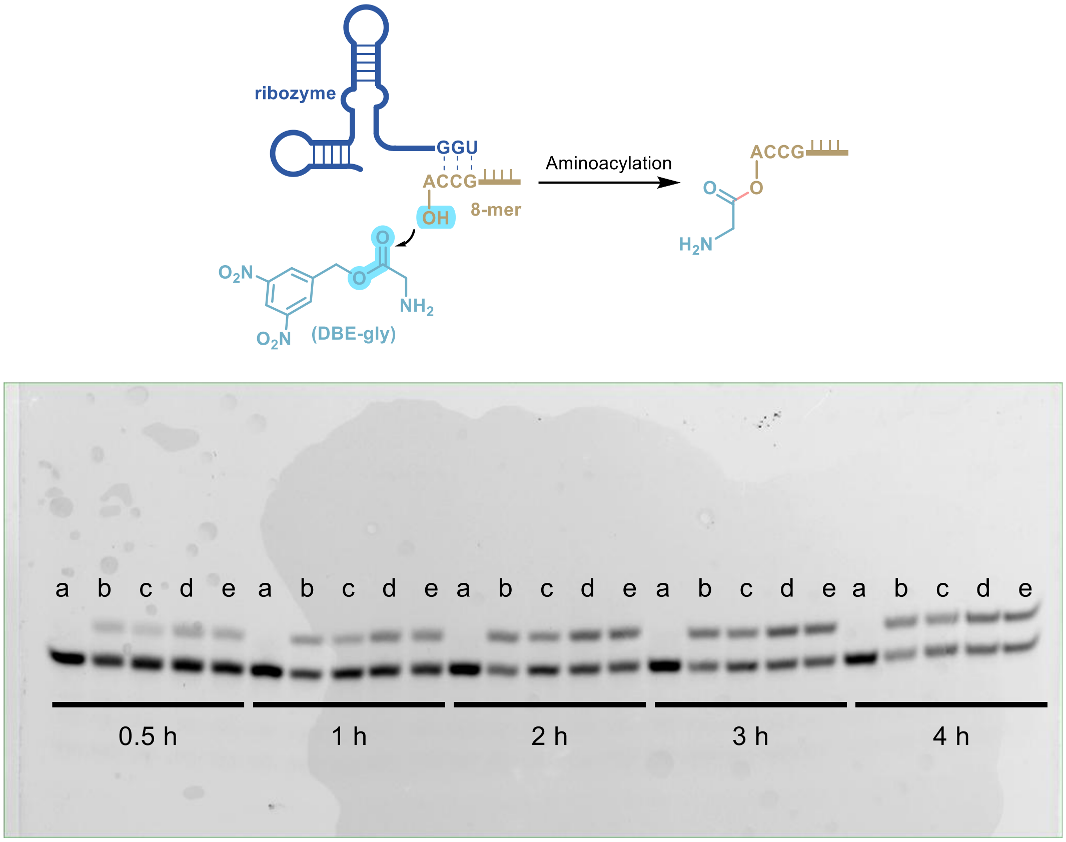

a: negative control without Flexizyme
b: dFx
c: dFx-mut
d: dFx-mut-a
e: dFx-mut-a*

Figure S24. PAGE image of Flexizyme-catalyzed aminoacylation. dFx, a reference ribozyme with previously demonstrated catalytic efficacy. **dFx-mut**, a mutated version prepared via RNA synthesizer. **dFx-mut-a**, a mutated version prepared via *in situ* phosphate activation and loop-closing ligation. **dFx-mut-a***, dFx-mut-a after heating-treatment at 95 °C for 1 hour to remove potential base modifications introduced during *in situ* activation and ligation. Reaction conditions for aminoacylation: 8-mer RNA substrate (0.5 µM), Flexizyme (0.5 µM), HEPES (100 mM, pH 8.0), $MgCl_2$ (100 mM), DBE-gly (5 mM) at 0 °C.

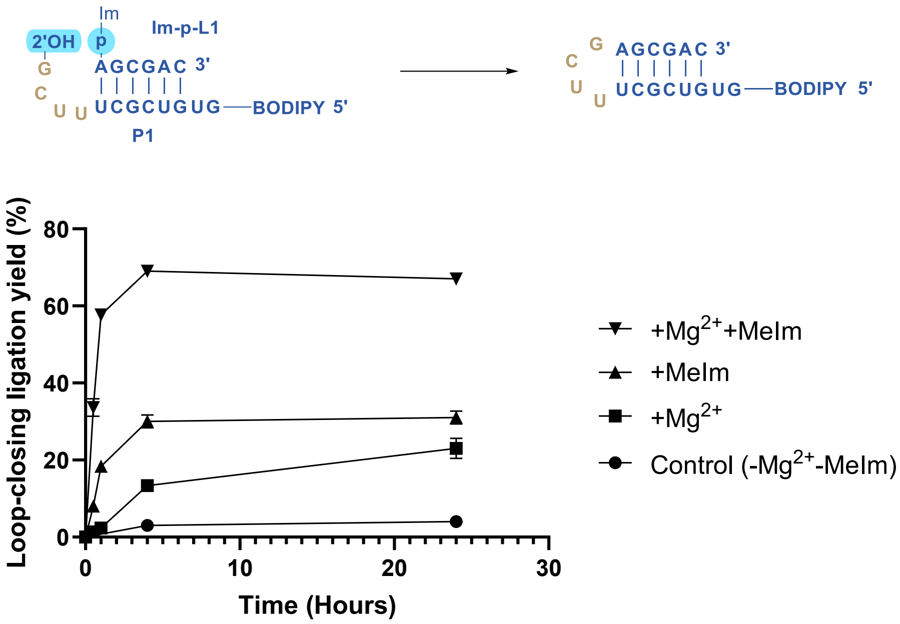

Figure S25. Loop-closing ligations using pre-activated ligator Im-p-L1. Error bars represent standard deviations from the mean, n = 3 replicates.

Control (-Mg^{2+}-MeIm) : P1 (1 μM), Im-p-L1 (1 μM), NaCl (100 mM) at 18 °C.

+Mg^{2+}: P1 (1 μM), Im-p-L1 (1 μM), $MgCl_2$ (50 mM), NaCl (100 mM) at 18 °C.

+MeIm: P1 (1 μM), Im-p-L1 (1 μM), *N*-methylimidazole (200 mM, pH 8.0), NaCl (100 mM) at 18 °C.

+Mg^{2+}+MeIm: P1 (1 μM), Im-p-L1 (1 μM), $MgCl_2$ (50 mM), *N*-methylimidazole (200 mM, pH 8.0), NaCl (100 mM) at 18 °C.

Characterization of *N*-imidoyl-*N'*-methylimidazolium (IMI) 10. The minor isomer is denoted by prime.

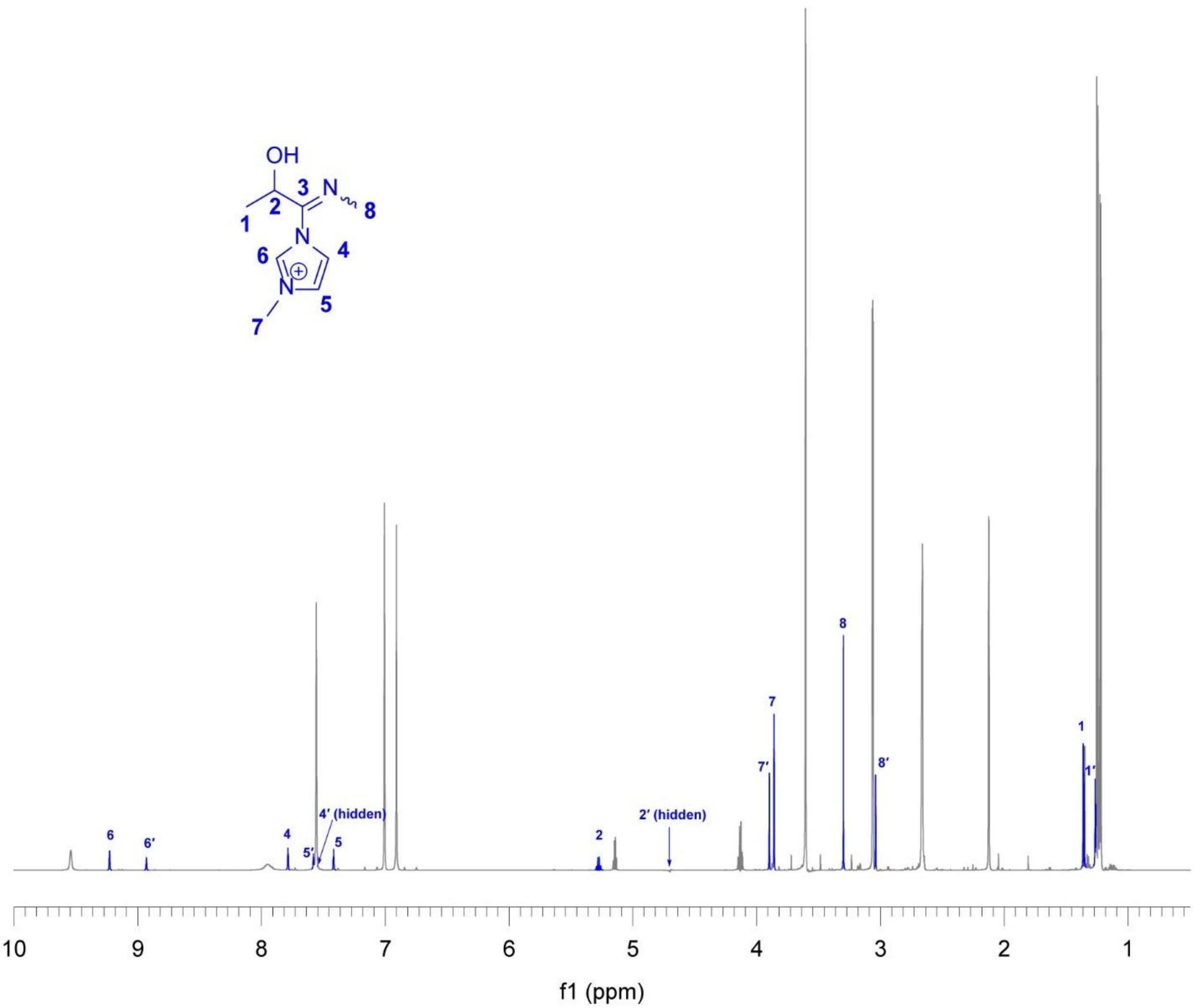

Figure S26. ^{1}H NMR of imidazolium 10 (600 MHz, 10% D_2O in H_2O).

[**major isomer**] δ 9.23 (s, 1H), 7.79-7.78 (m, 1H), 7.42-7.41 (m, 1H), 5.28 (q, *J* = 7.2 Hz, 1H), 3.86 (s, 3H), 3.30 (s, 3H), 1.36 (d, *J* = 7.2 Hz, 3H).

[**minor isomer**] δ 8.93 (s, 1H), 7.584-7.578 (m, 1H), 7.55 (1H, hidden), 4.64 (1H, hidden), 3.90 (s, 3H), 3.04 (s, 3H), 1.26 (d, *J* = 7.2 Hz, 3H).

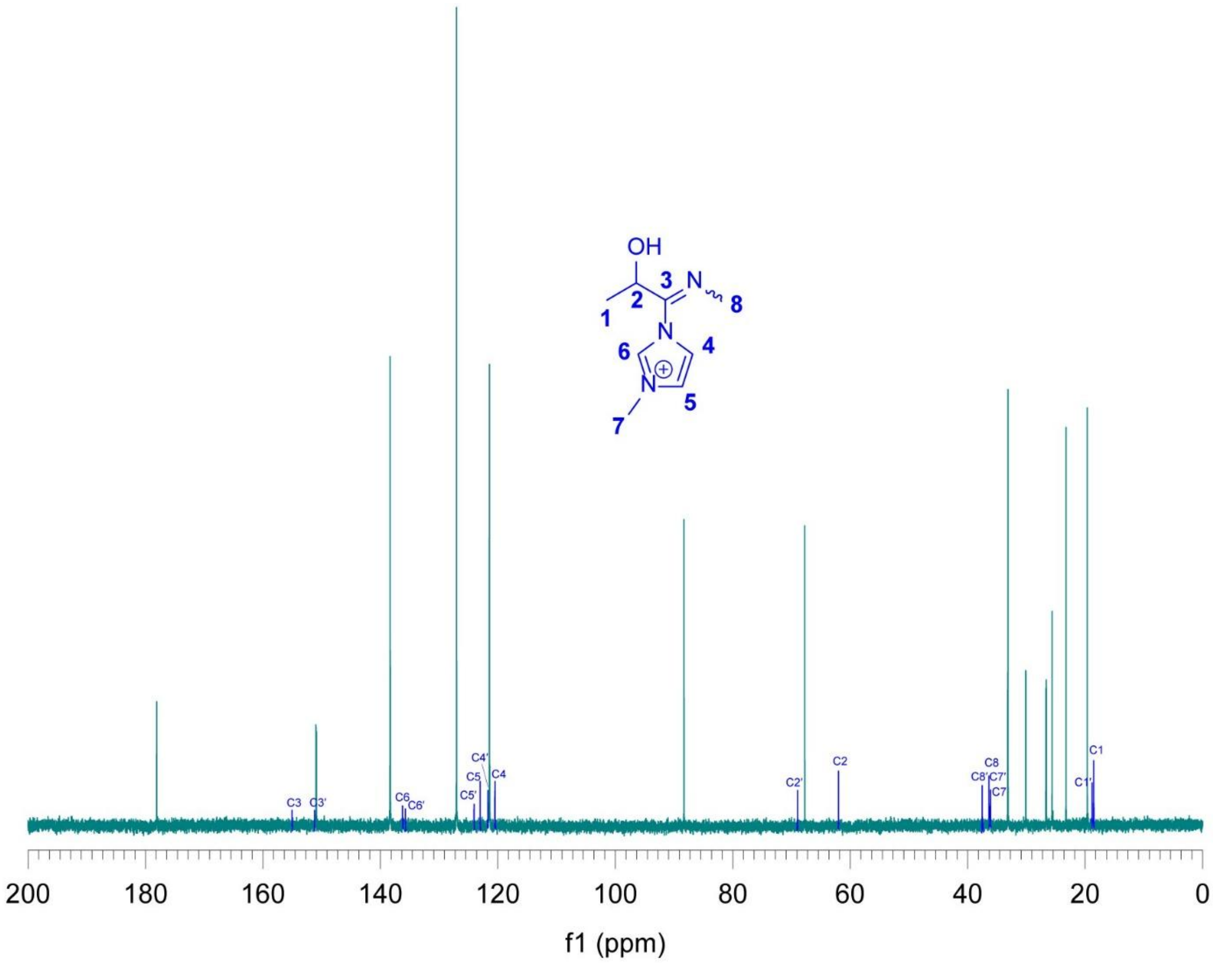

Figure S27. ^{13}**C NMR of imidazolium 10 (150 MHz, 10% D_2O in H_2O).**

[**major isomer**] δ 155.0 (C3), 136.2 (C6), 123.0 (C5), 120.5 (C4), 62.0 (C2), 36.4 (C8), 36.1 (C7), 18.6 (C1).

[**minor isomer**] δ 151.2 (C3′), 135.8 (C6′), 124.1 (C5′), 121.7 (C4′), 69.0 (C2′), 37.5 (C8′), 36.2 (C7′), 18.8 (C1′).

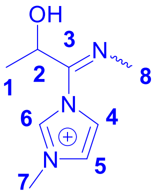

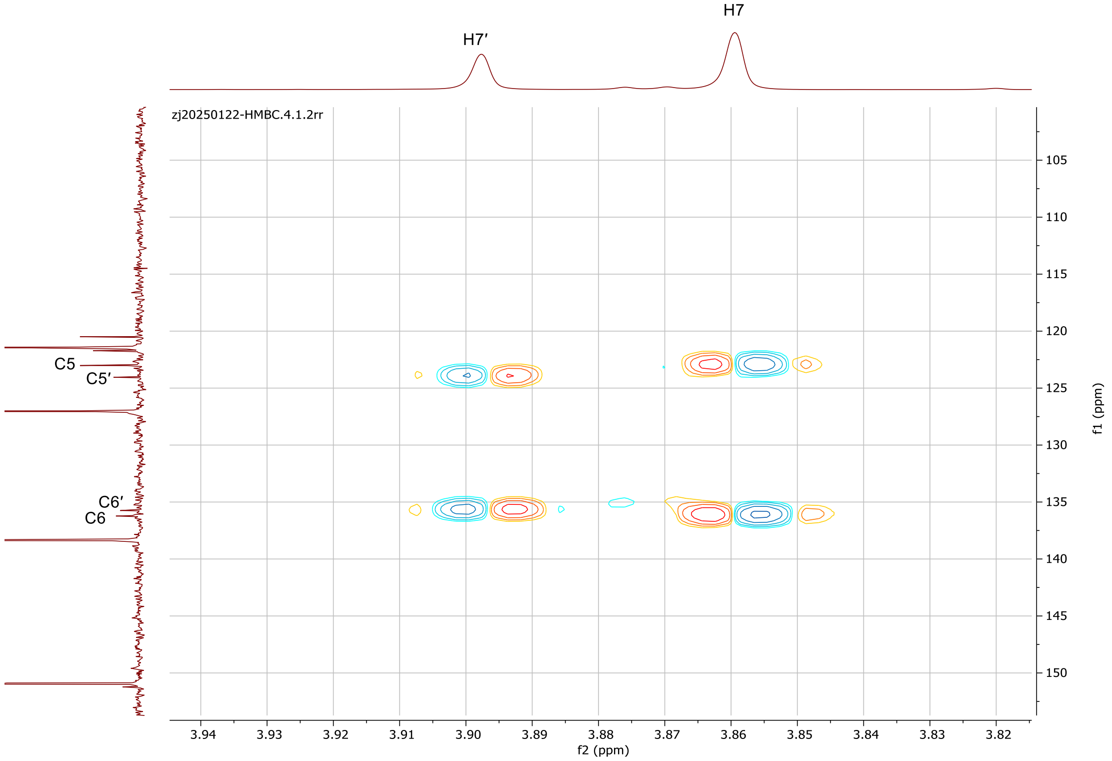

Figure S28. HMBC correlation spectrum of 10 showing the three-bond correlation of H7′-C5′ (3.90, 124.1 ppm), H7′-C6′ (3.90, 135.8 ppm), H7-C5 (3.86, 123.0 ppm), H7-C6 (3.86, 136.2 ppm).

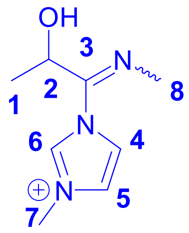

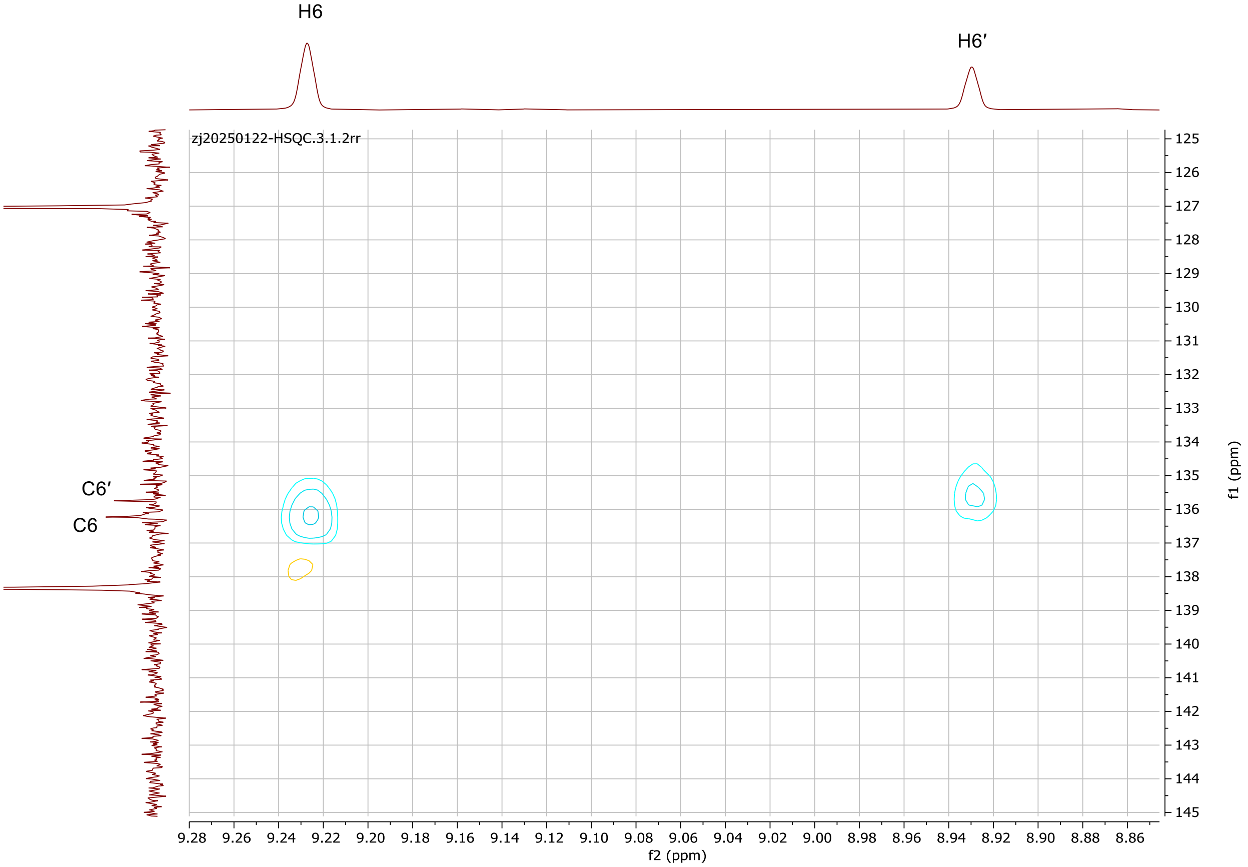

Figure S29. HSQC correlation spectrum of 10 showing the one-bond correlation of H6-C6 (9.23, 136.2 ppm), H6′-C6′ (8.93, 135.8 ppm).

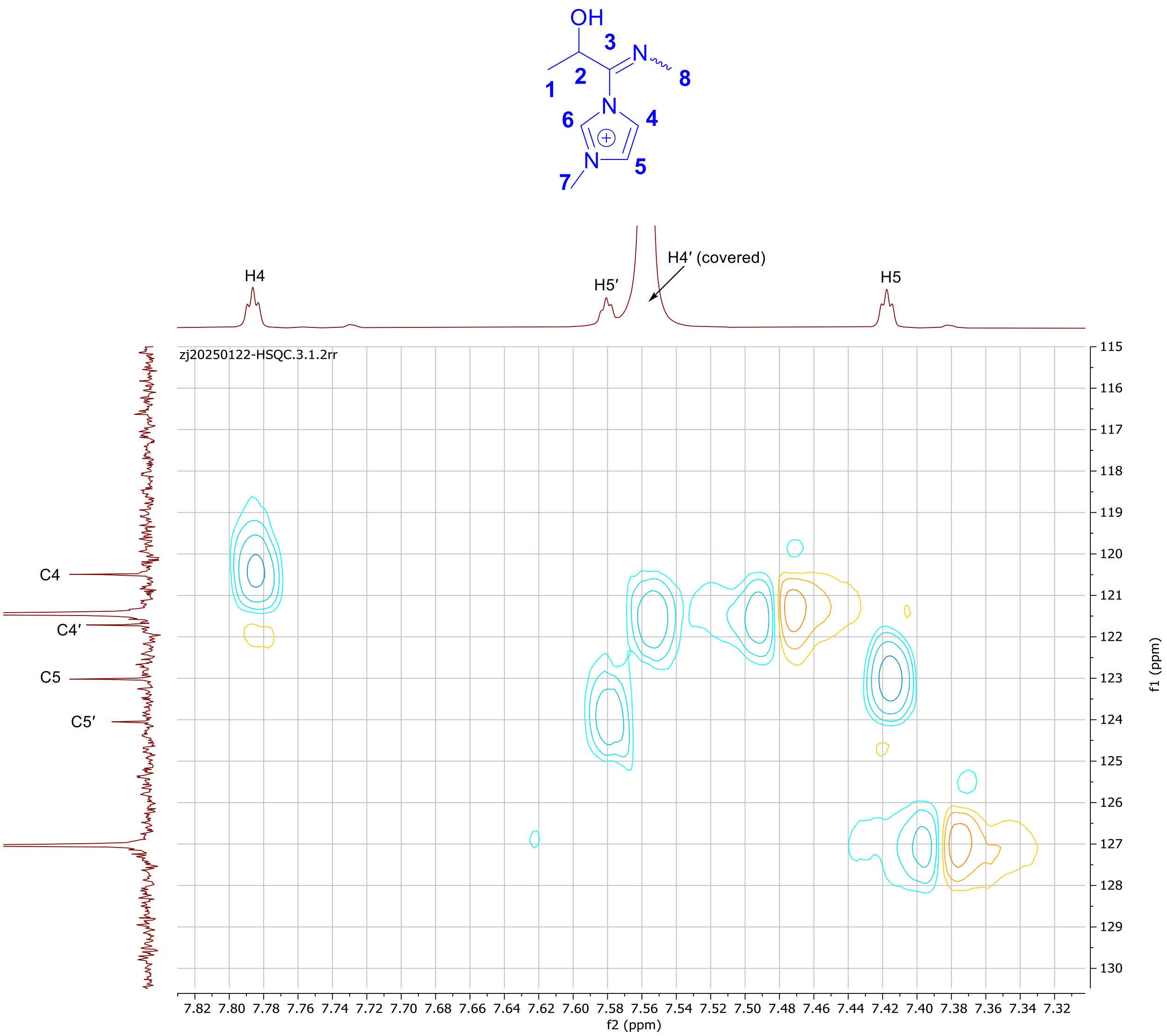

Figure S30. HSQC correlation spectrum of 10 showing the one-bond correlation of H4-C4 (7.79, 120.5 ppm), H5′-C5′ (7.58, 124.1 ppm), H4′-C4′ (7.55, 121.7 ppm), H5-C5 (7.42, 123.0 ppm).

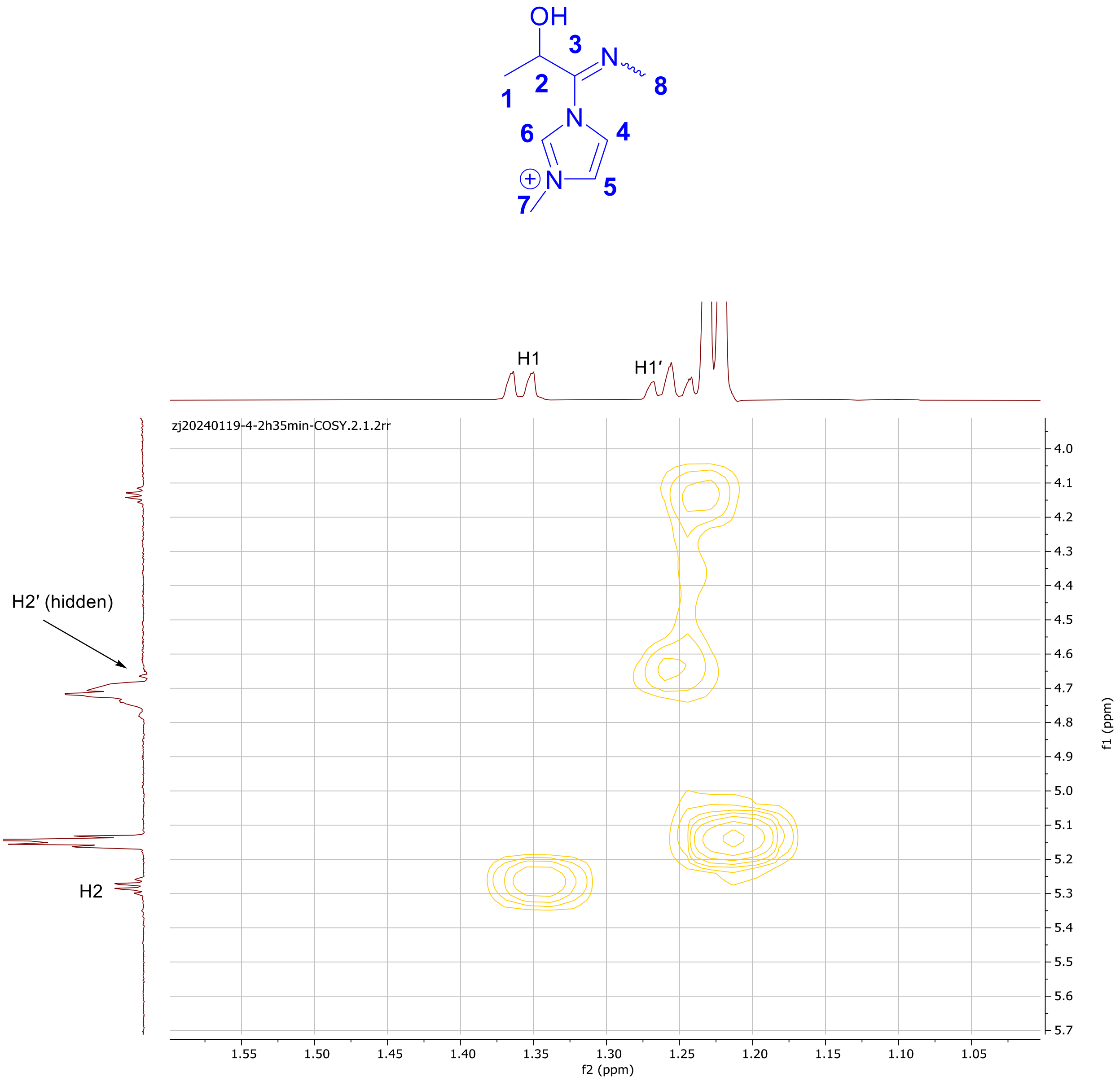

Figure S31. H-H COSY correlation spectrum of 10 showing the three-bond correlation of H1-H2 (1.36, 5.28 ppm), H1′-H2′ (1.26, 4.64 ppm).

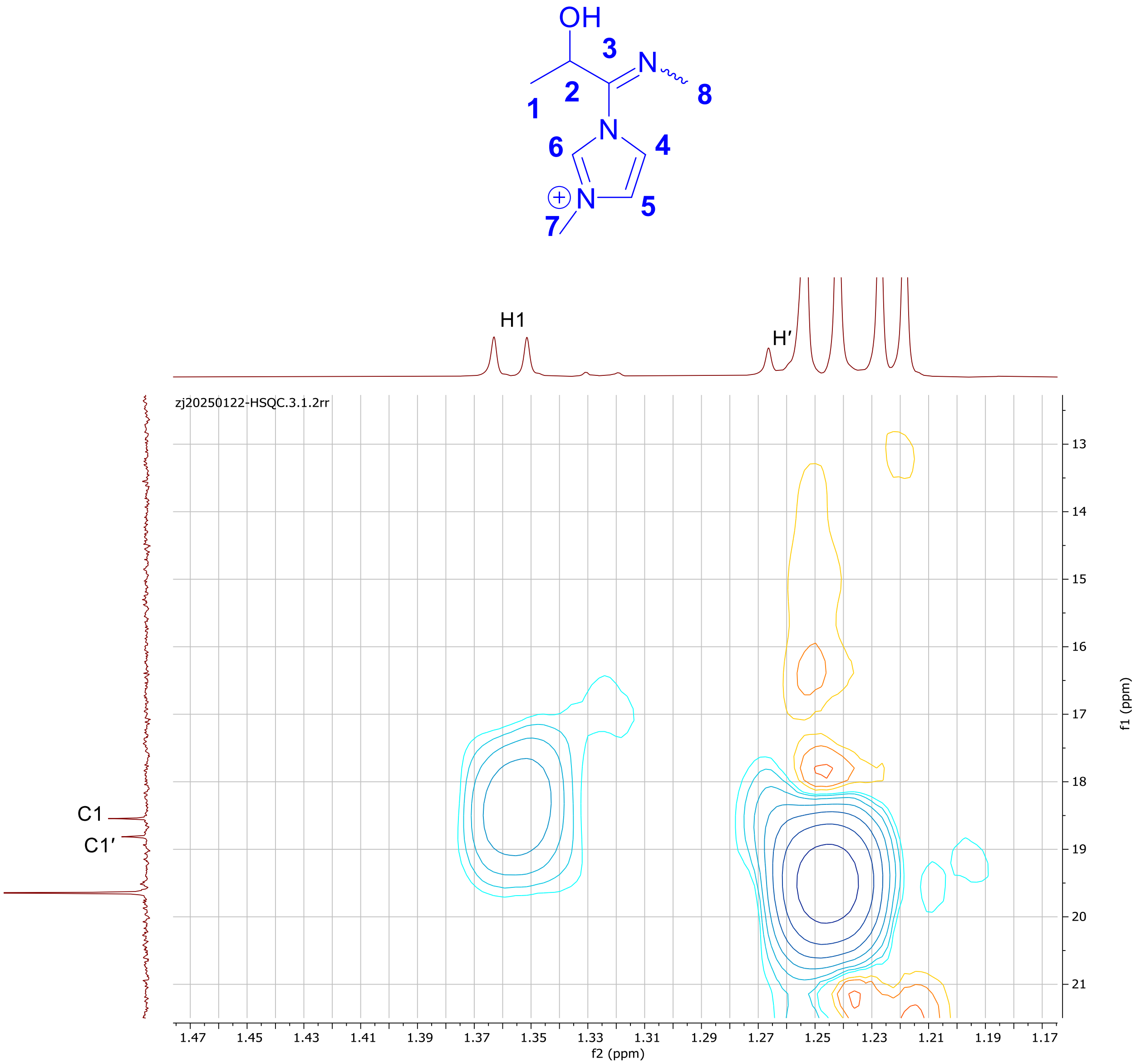

Figure S32. HSQC correlation spectrum of 10 showing the one-bond correlation of H1-C1 (1.36, 18.6 ppm), H1′-C1′ (1.26, 18.8 ppm).

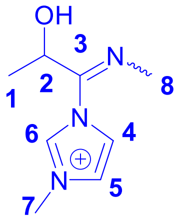

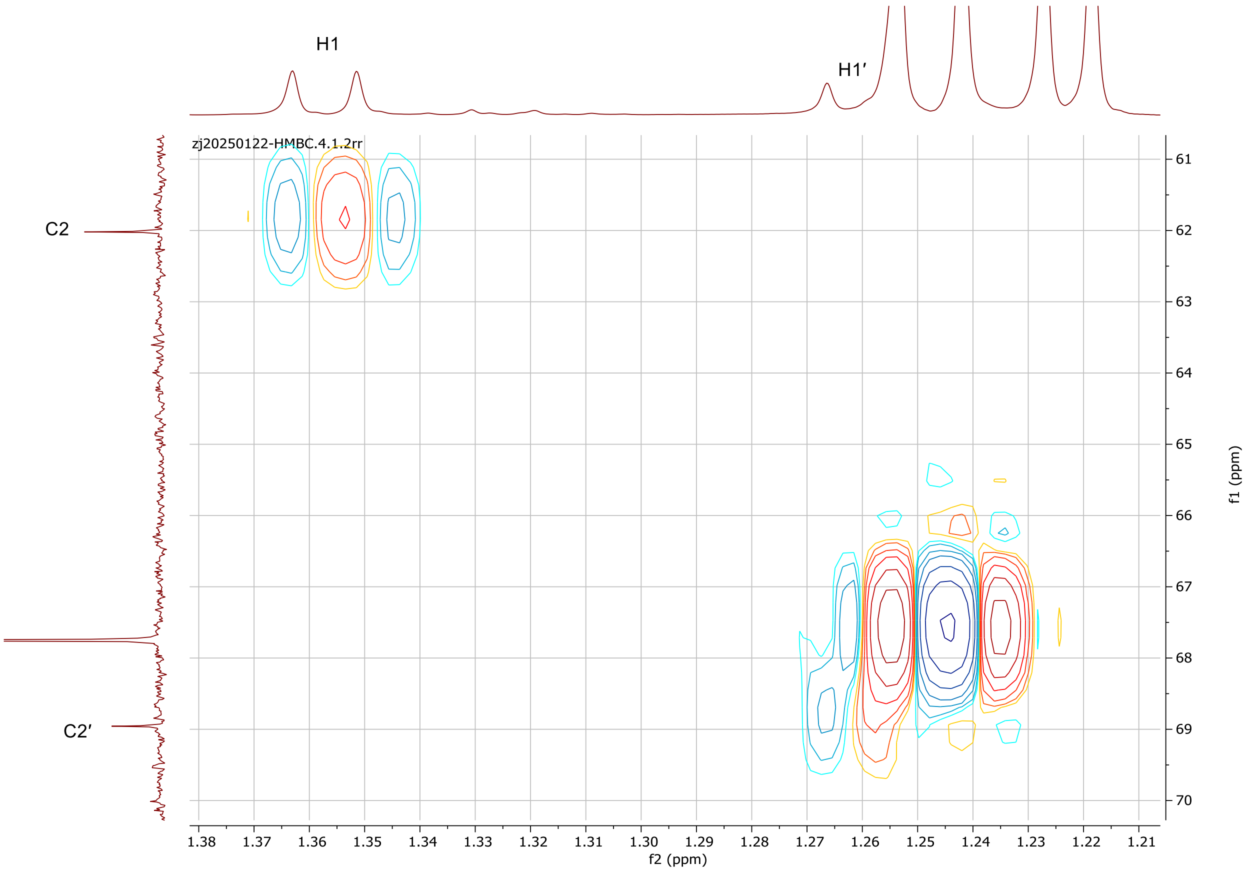

Figure S33. HMBC correlation spectrum of 10 showing the two-bond correlation of H1-C2 (1.36, 62.0 ppm), H1′-C2′ (1.26, 69.0 ppm).

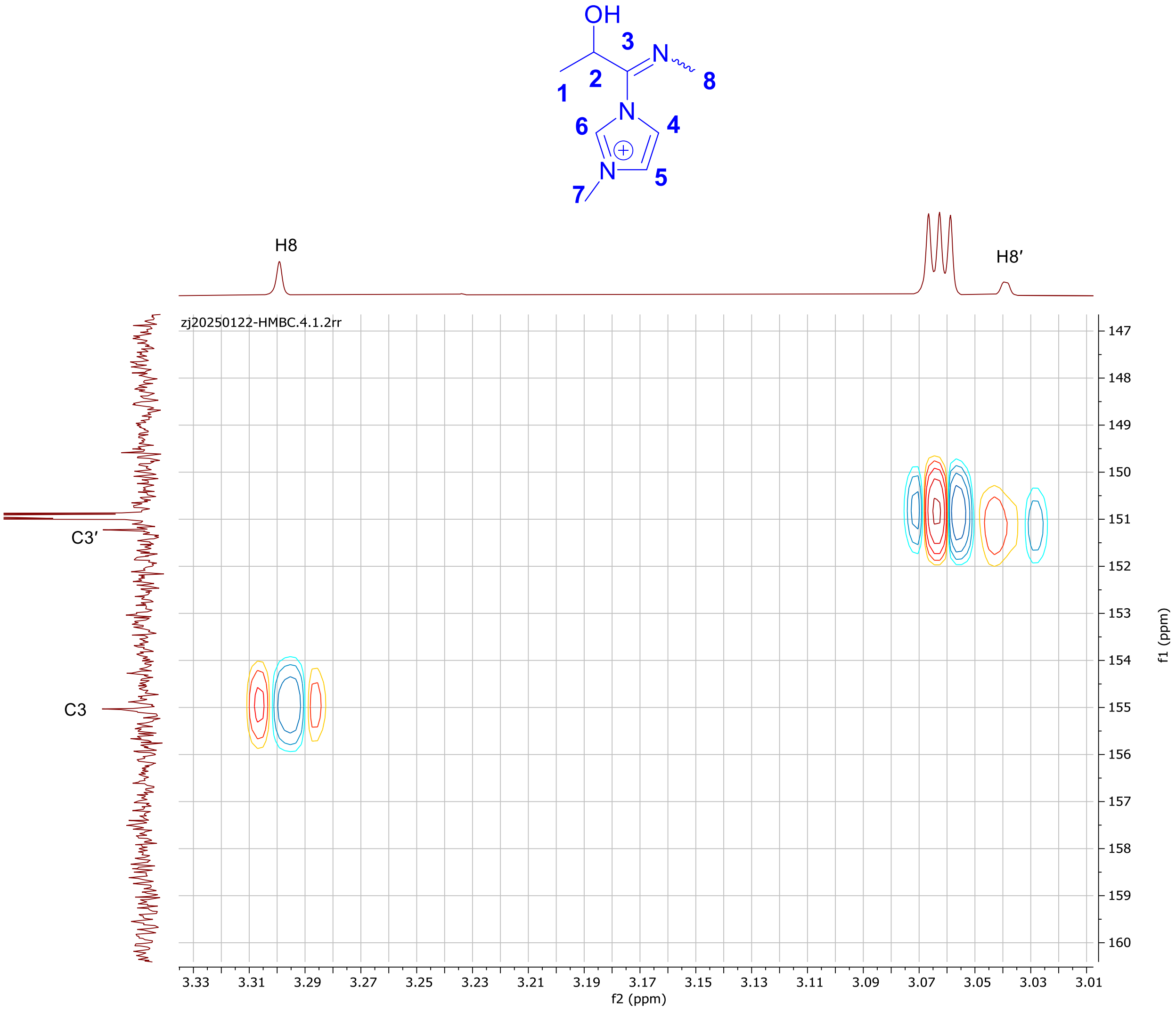

Figure S34. HMBC correlation spectrum of 10 showing the three-bond correlation of H8-C3 (3.30, 155.0 ppm), H8′-C3′ (3.04, 151.2 ppm).

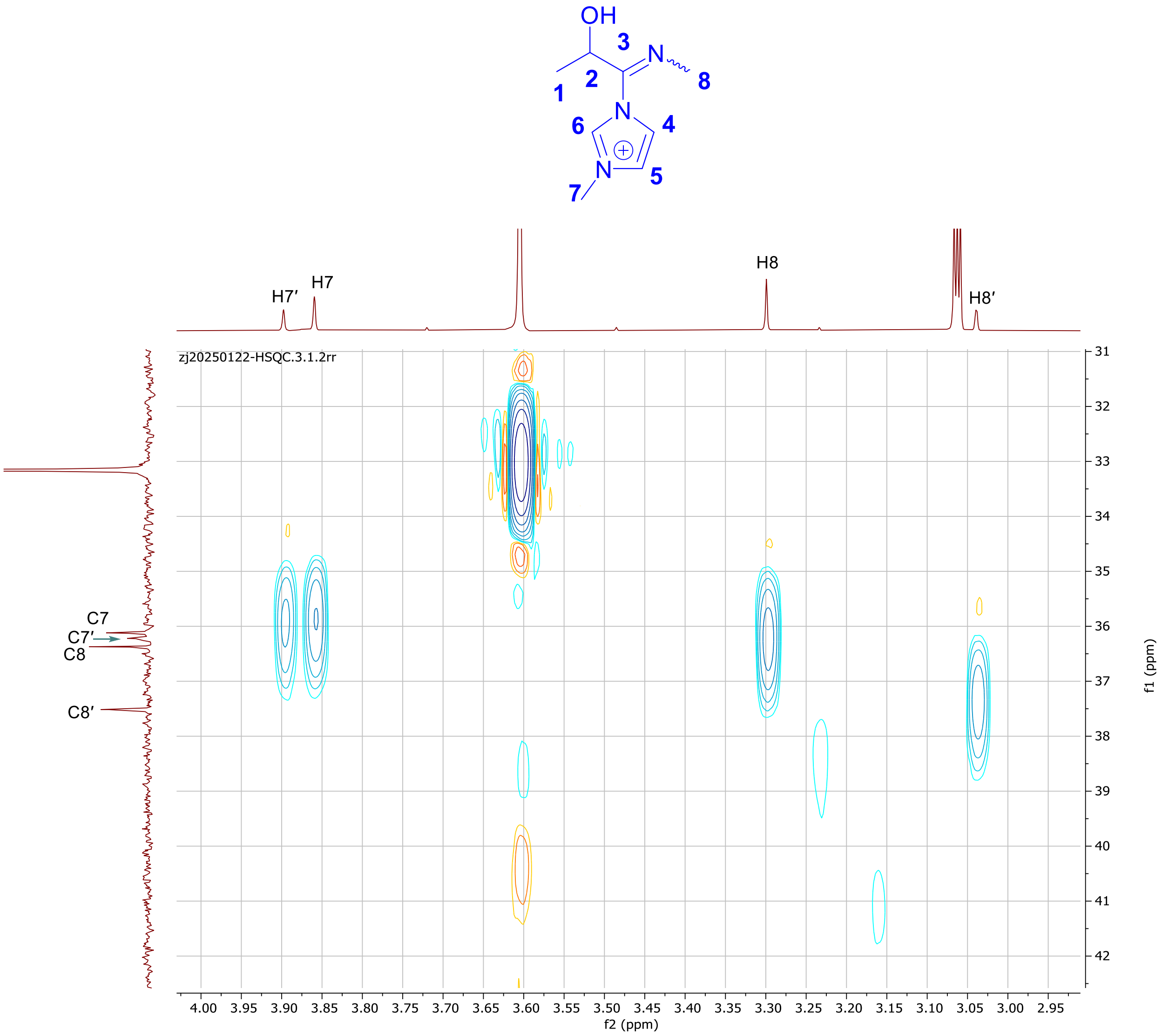

Figure S35. HSQC correlation spectrum of 10 showing the one-bond correlation of H7′-C7′ (3.90, 36.2 ppm), H7-C7 (3.86, 36.1 ppm), H8-C8 (3.30, 36.4 ppm), H8′-C8′ (3.04, 37.5 ppm).

Characterization of *N*-imidoyl-*N′*-methyl imidazolium (IMI) 11. The minor isomer is denoted by prime.

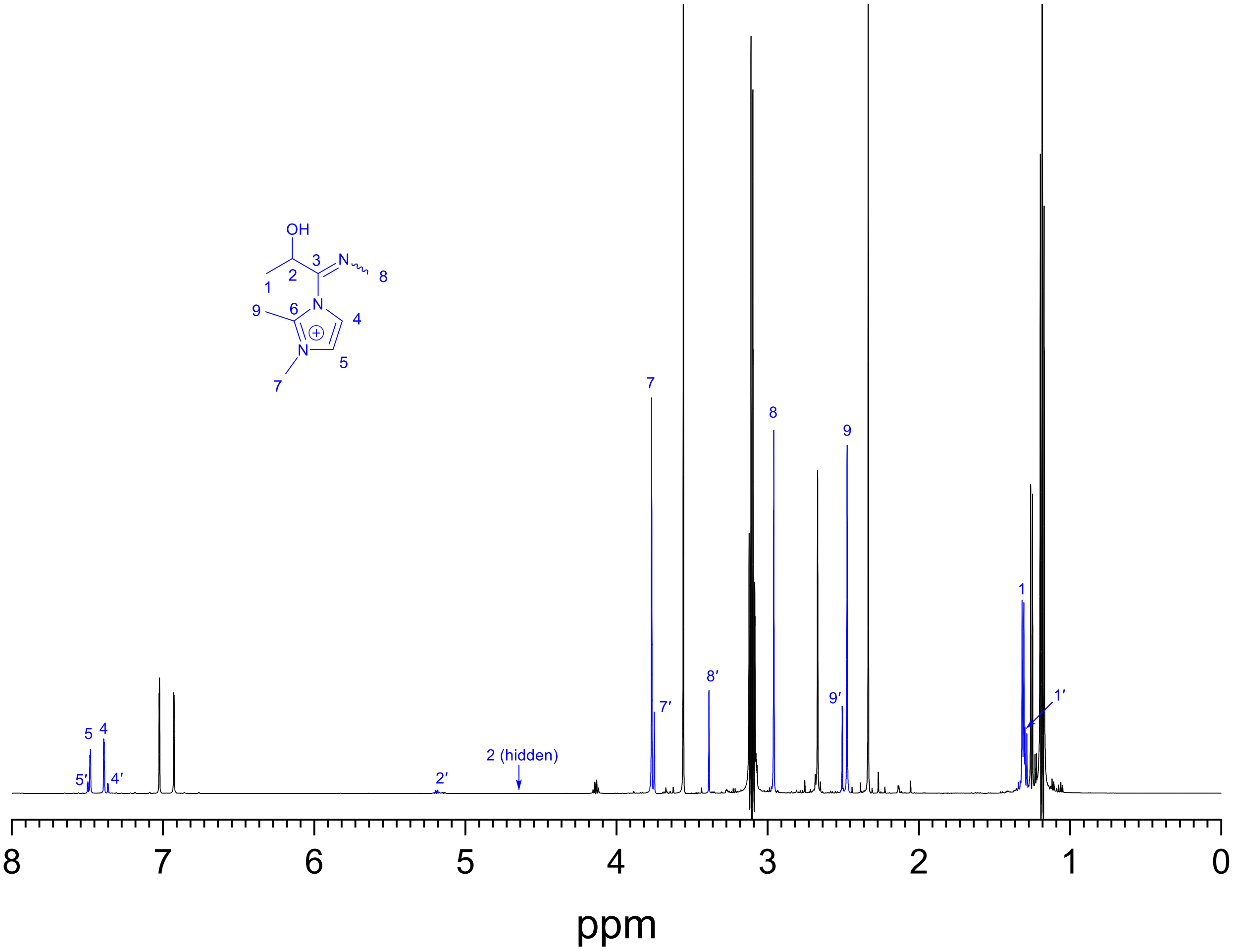

Figure S36. ^{1}H NMR of intermediate 11 (600 MHz, 10% D_2O in H_2O).

[**major isomer**] δ 7.484-7.480 (m, 1H), 7.39-7.38 (m, 1H), 4.61 (hidden, 1H), 3.77 (s, 3H), 2.96 (s, 3H), 2.48 (s, 3H), 1.31 (d, *J* = 7.2 Hz, 3H).

[**minor isomer**] δ 7.50-7.49 (m, 1H), 7.37-7.36 (m, 1H), 5.19 (q, *J* = 7.2 Hz, 1H), 3.75 (s, 3H), 3.39 (s, 3H), 2.51 (s, 3H), 1.29 (d, *J* = 7.2 Hz, 3H).

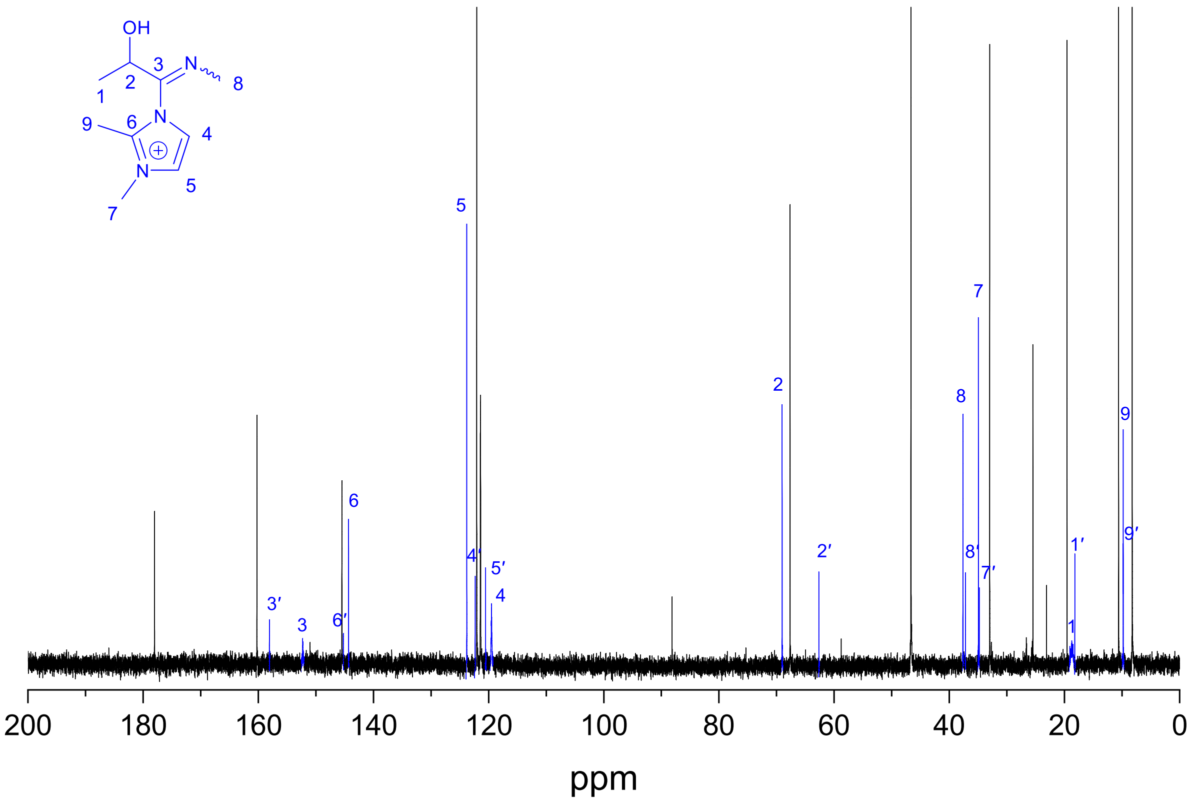

Figure S37. ^{13}**C NMR of intermediate 11 (150 MHz, 10% D_2O in H_2O).**

[**major isomer**] δ 152.4 (C3), 144.3 (C6), 123.8 (C5), 119.5 (C4), 69.0 (C2), 37.6 (C8), 34.9 (C7), 18.9 (C1), 9.79 (C9).

[**minor isomer**] δ 158.1 (C3′), 145.2 (C6′), 122.3 (C4′), 120.5 (C5′), 62.7 (C2′), 37.2 (C8′), 34.8 (C7′), 18.2 (C1′), 9.82 (C9′).

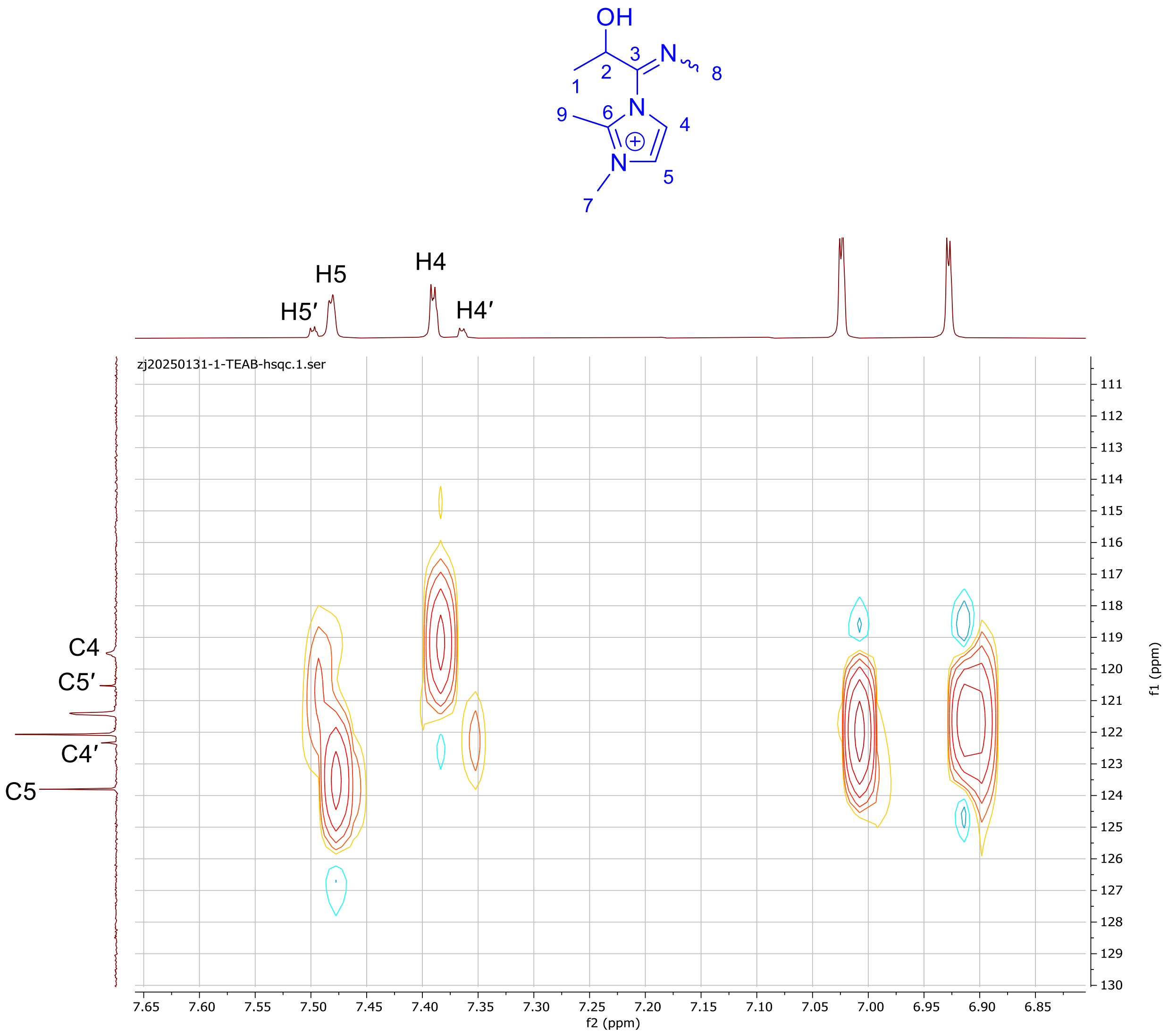

Figure S38. HSQC correlation spectrum of 11 showing the one-bond correlation of H5′-C5′ (7.50, 120.5 ppm), H5-C5 (7.48, 123.8 ppm), H4-C4 (7.39, 119.5 ppm), H4′-C4′ (7.36, 122.3 ppm).

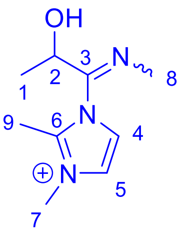

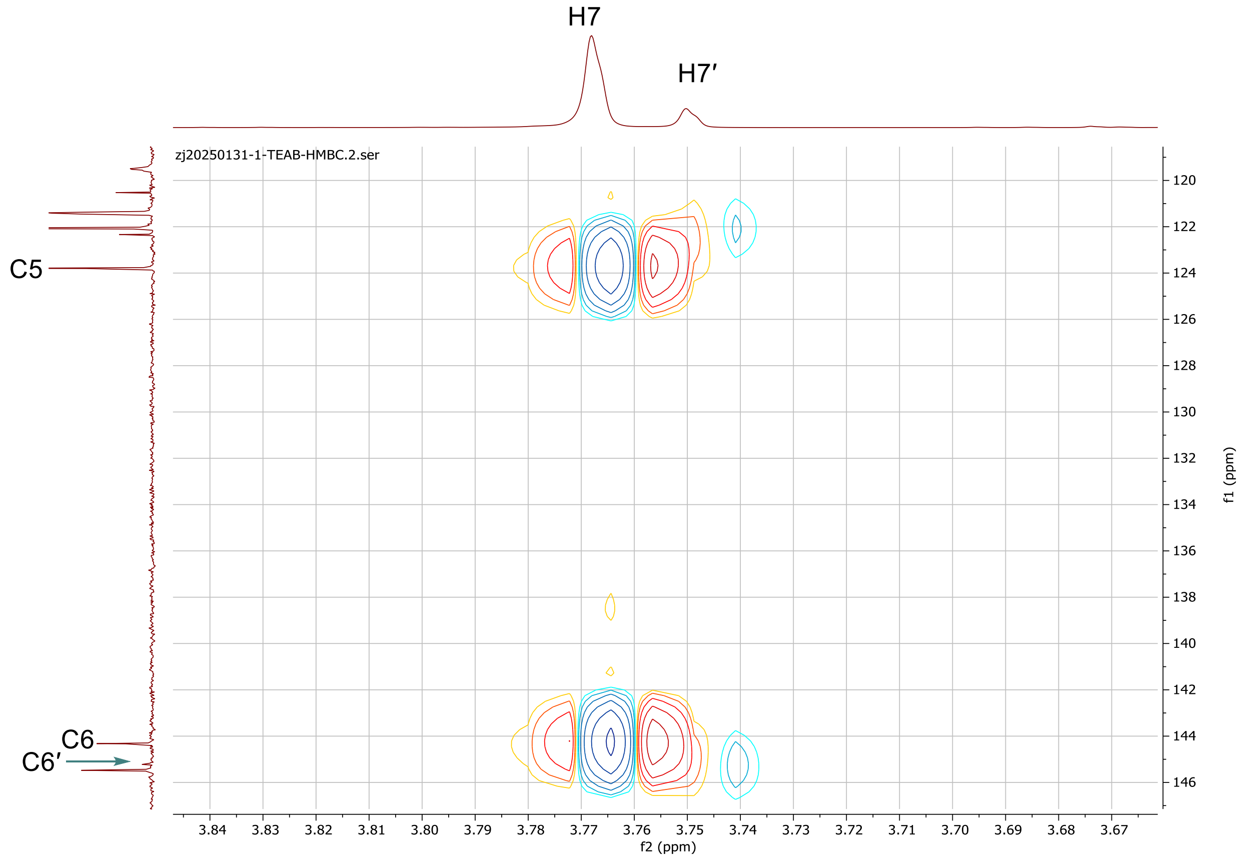

Figure S39. HMBC correlation spectrum of 11 showing the three-bond correlation of H7-C5 (3.77, 123.8 ppm), H7-C6 (3.77, 144.3 ppm).

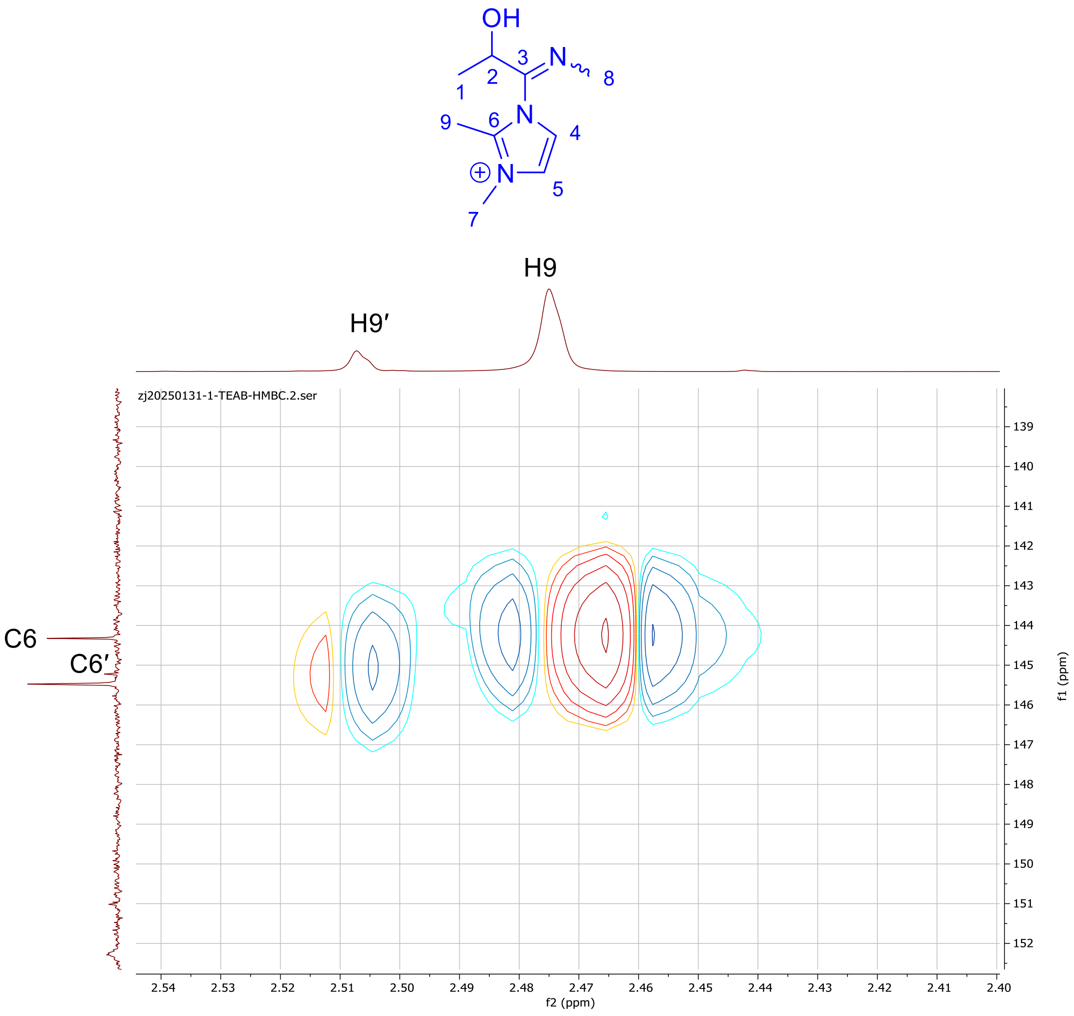

Figure S40. HMBC correlation spectrum of 11 showing the two-bond correlation of H9′-C6′ (2.51, 145.2 ppm), H9-C6 (2.48, 144.3 ppm).

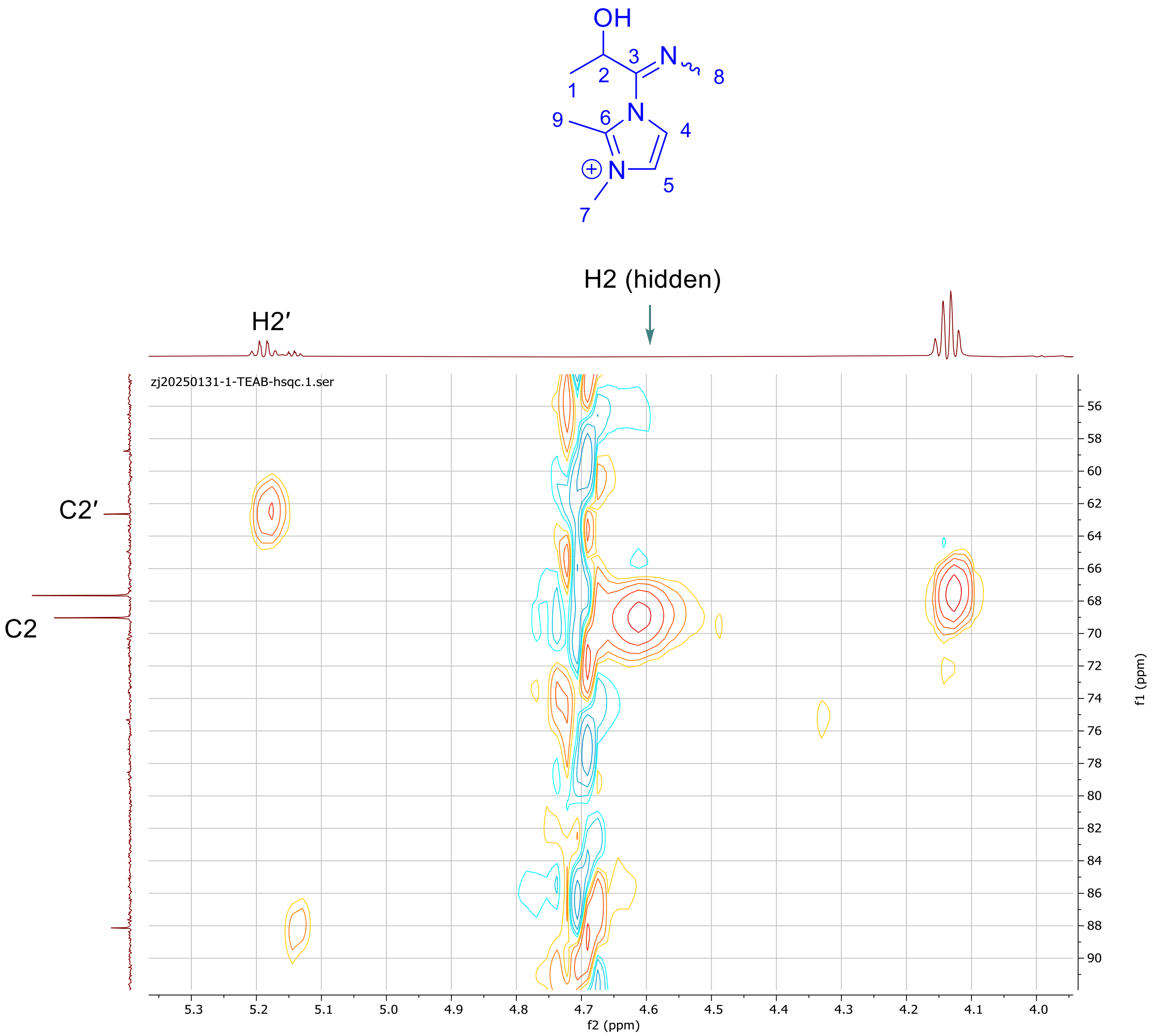

Figure S41. HSQC correlation spectrum of 11 showing the one-bond correlation of H2′-C2′ (5.19, 62.7 ppm), H2-C2 (4.61, 69.0 ppm).

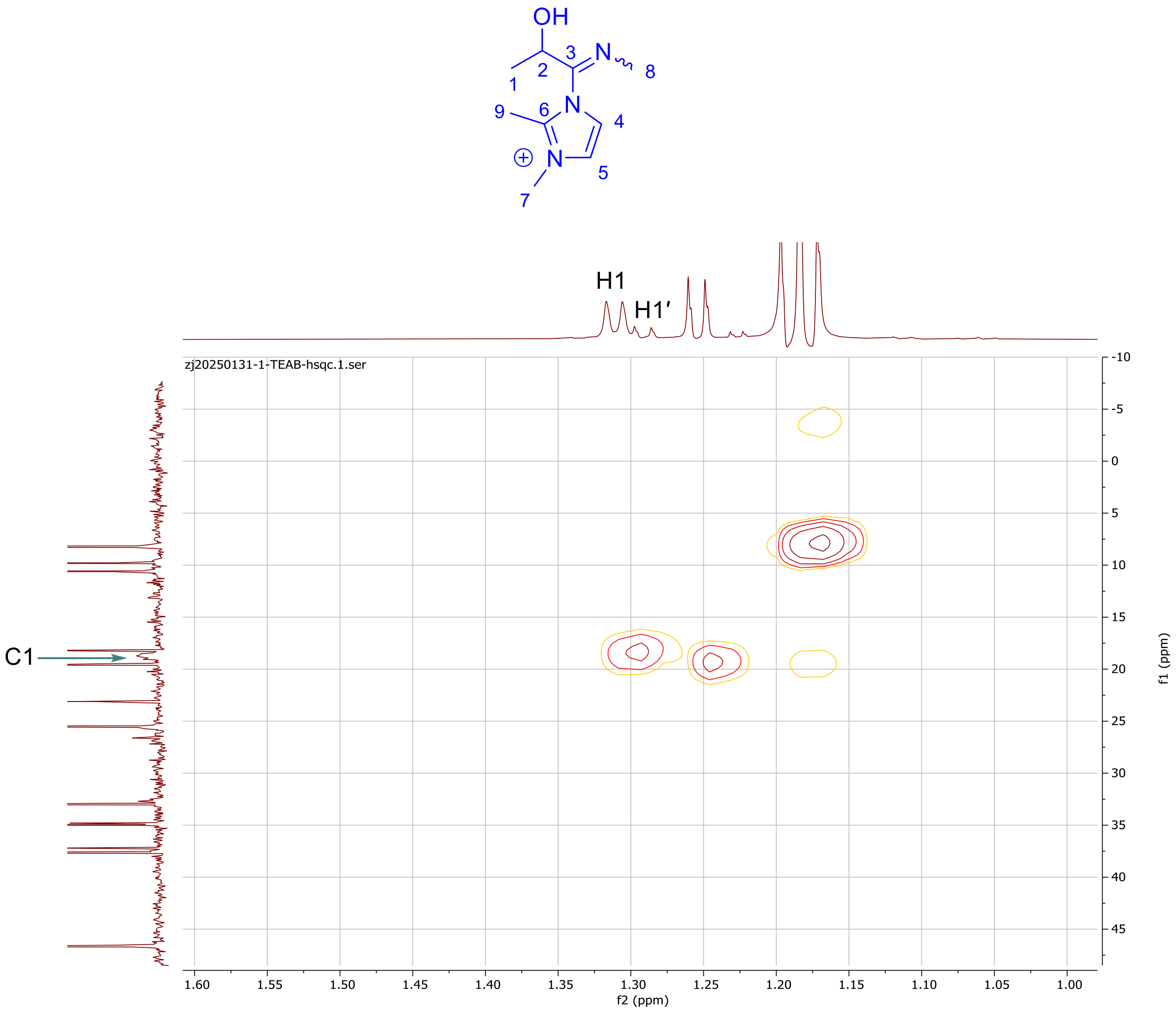

Figure S42. HSQC correlation spectrum of 11 showing the one-bond correlation of H1-C1 (1.31, 18.9 ppm).

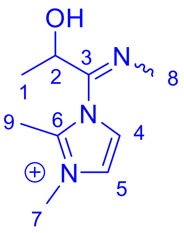

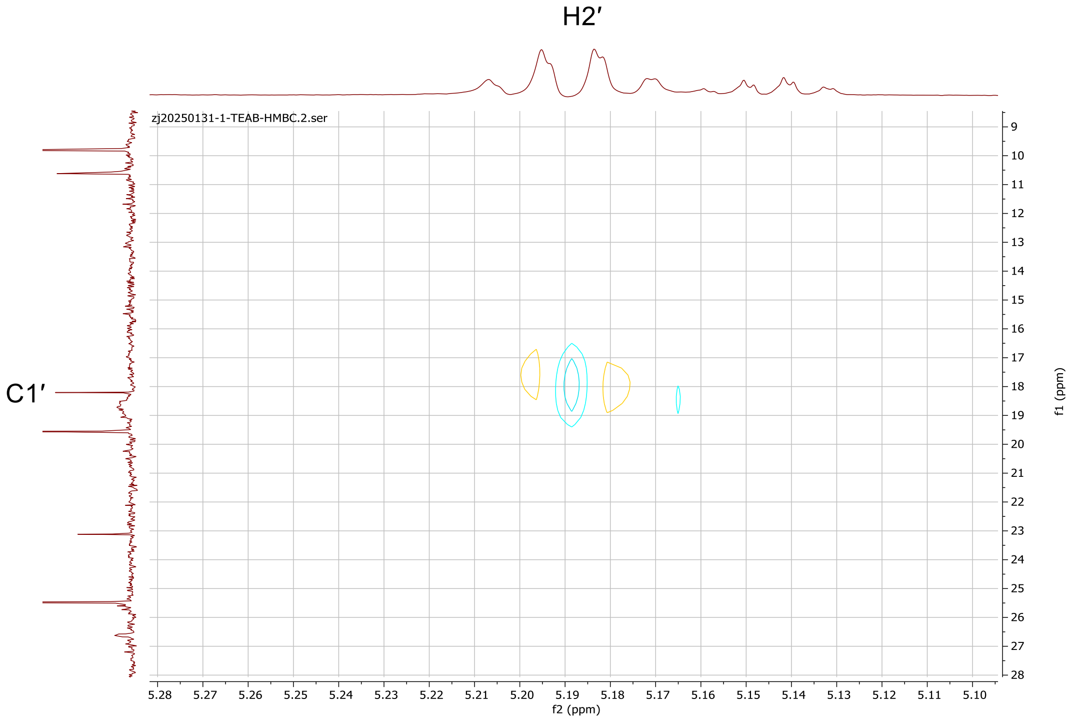

Figure S43. HMBC correlation spectrum of 11 showing the two-bond correlation of H2′-C1′ (5.19, 18.2 ppm).

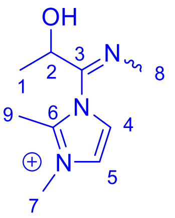

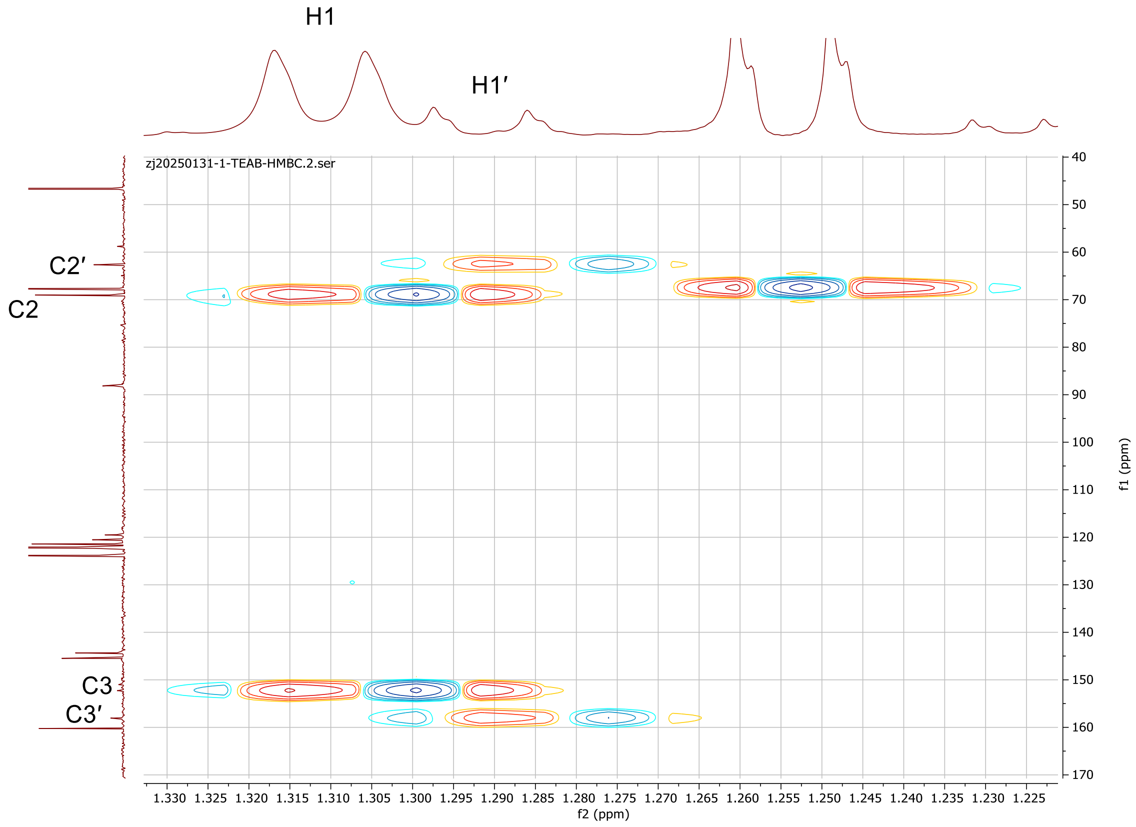

Figure S44. HMBC correlation spectrum of 11 showing the two-bond correlation of H1-C2 (1.31, 69.0 ppm), H1′-C2′ (1.29, 62.7 ppm) and three-bond correlation of H1-C3 (1.31, 152.4 ppm), H1′-C3′ (1.29, 158.1 ppm).

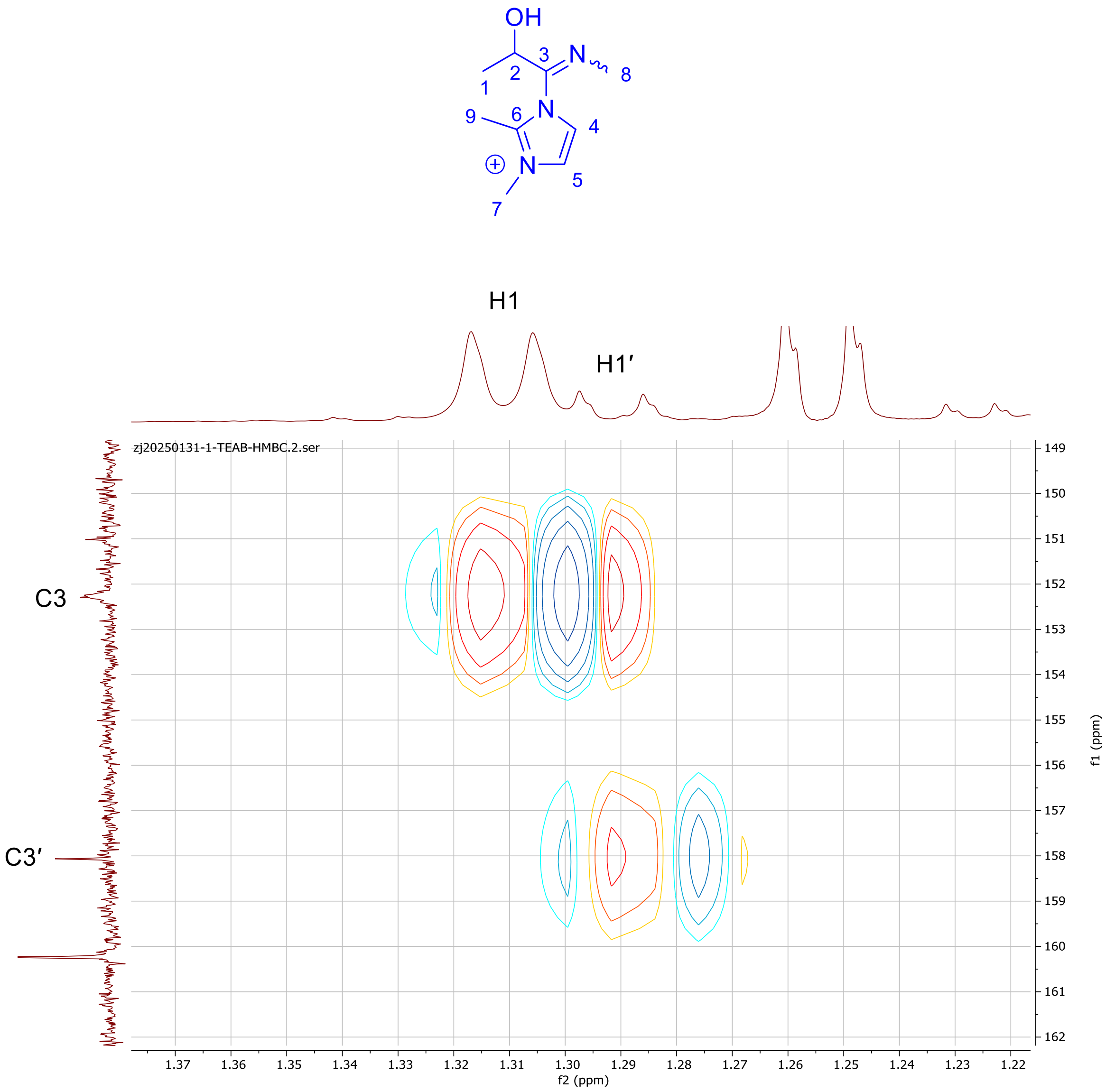

Figure S45. HMBC correlation spectrum of 11 showing three-bond correlation of H1-C3 (1.31, 152.4 ppm), H1′-C3′ (1.29, 158.1 ppm).

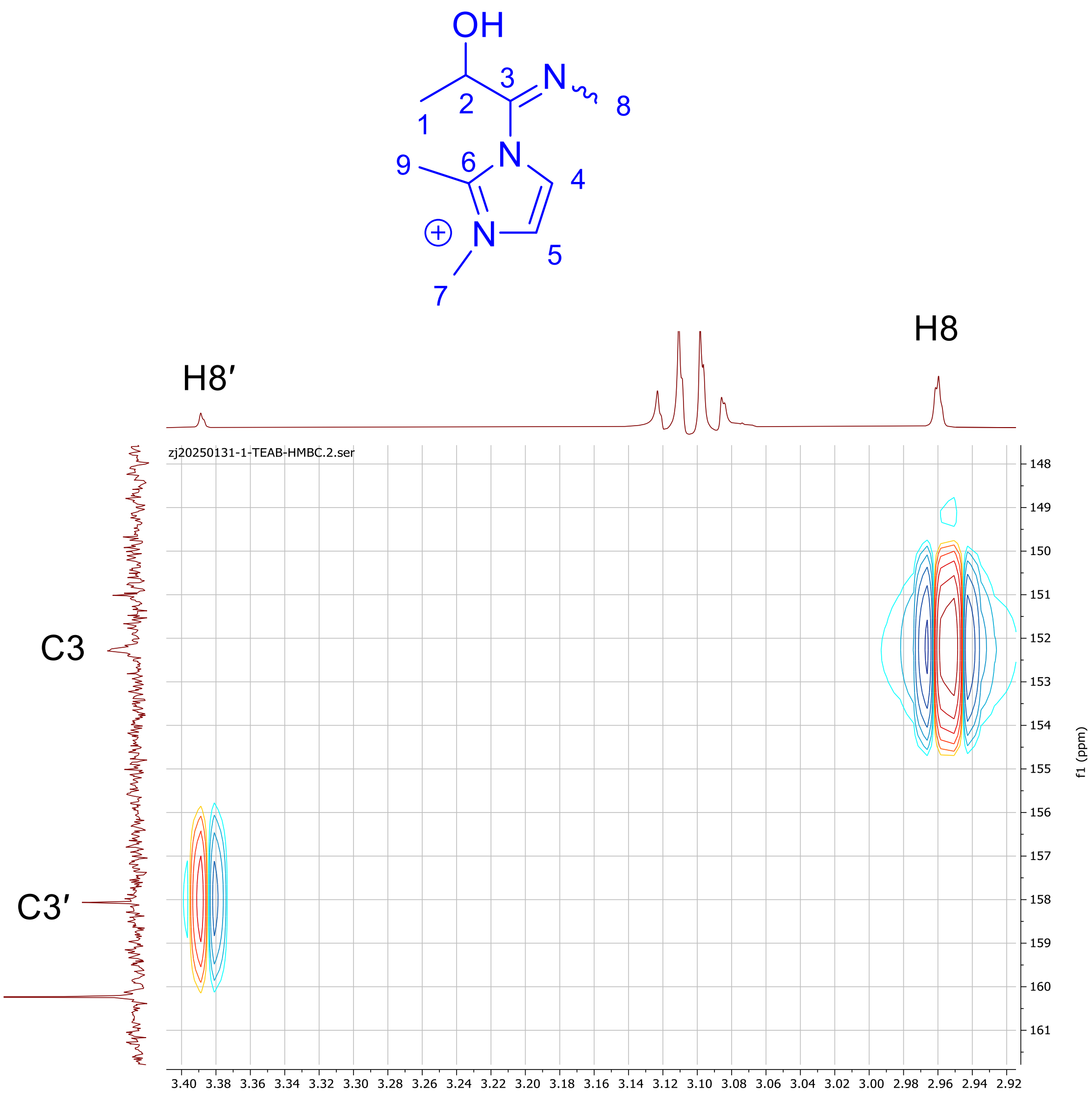

Figure S46. HMBC correlation spectrum of 11 showing the three-bond correlation of H8′-C3′ (3.39, 158.1 ppm), H8-C3 (2.96, 152.4 ppm).

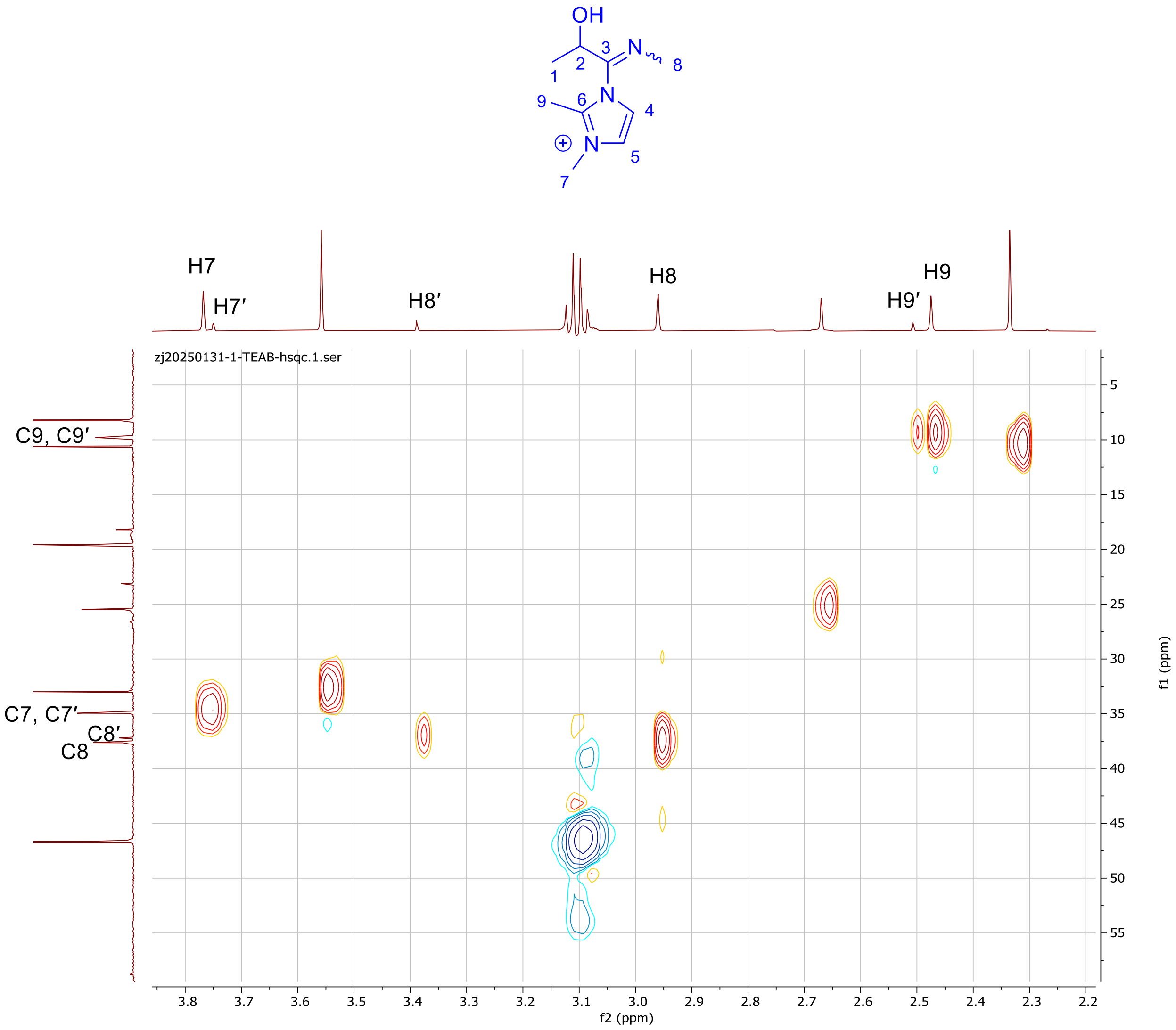

Figure S47. HSQC correlation spectrum of 11 showing the one-bond correlation of H7-C7 (3.77, 34.9 ppm), H8′-C8′ (3.39, 37.2 ppm), H8-C8 (2.96, 37.6 ppm), H9′-C9′ (2.51, 9.82 ppm), H9-C9 (2.48, 9.79 ppm).

Characterization of Passerini product 7

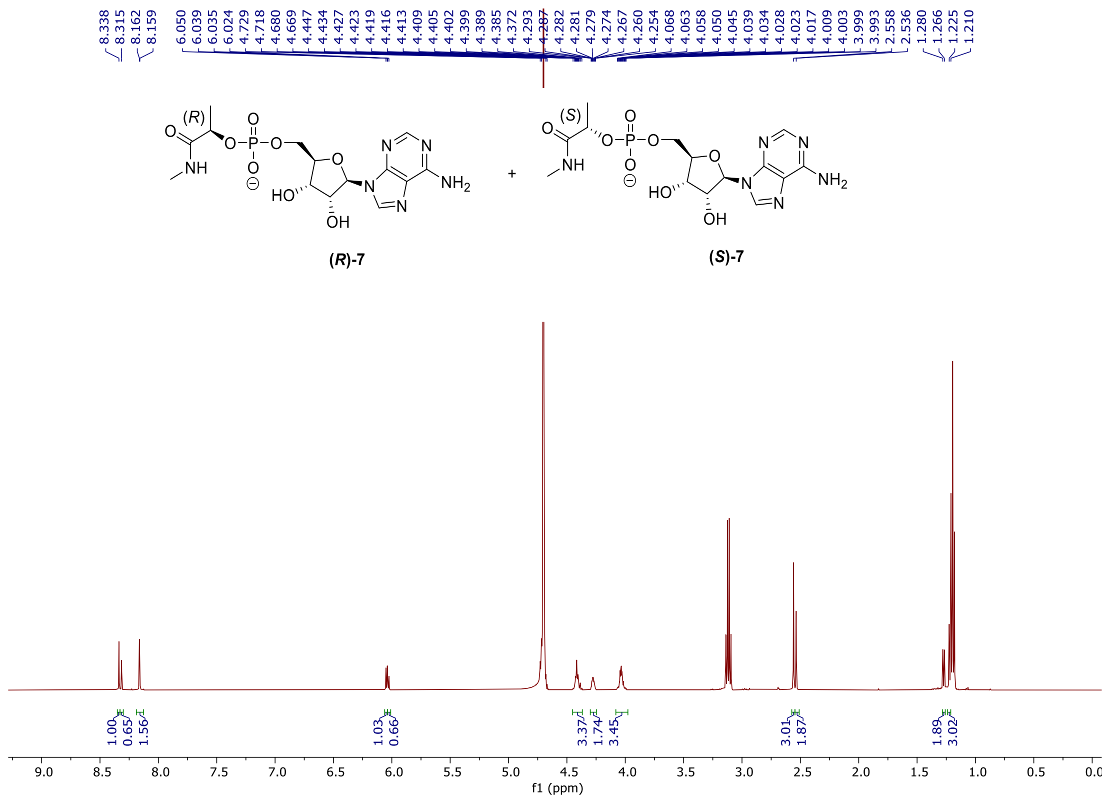

Figure S48. ¹H NMR of Passerini product 7 (600 MHz, D_2O). The ¹H NMR shows a mixture of diastereomers in a ratio of 1:0.6. This uneven ratio was introduced during the separation process and is not a result of the reaction itself. δ 8.34 (s, 1H), 8.32 (s, 0.6H), 8.162 (1H), 8.159 (0.6H), 6.05 (d, J = 6.6 Hz, 1H), 6.03 (d, J = 6.6 Hz, 0.6H), 4.73-4.67 (m, 1.6H, masked by H_2O peak), 4.44-4.37 (m, 3.2H), 4.29-4.25 (m, 1.6H), 4.07-3.99 (m, 3.2H), 2.56 (s, 3H), 2.54 (s, 1.8H), 1.27 (d, J = 8.2 Hz, 1.8H), 1.22(d, J = 8.2 Hz, 3H).

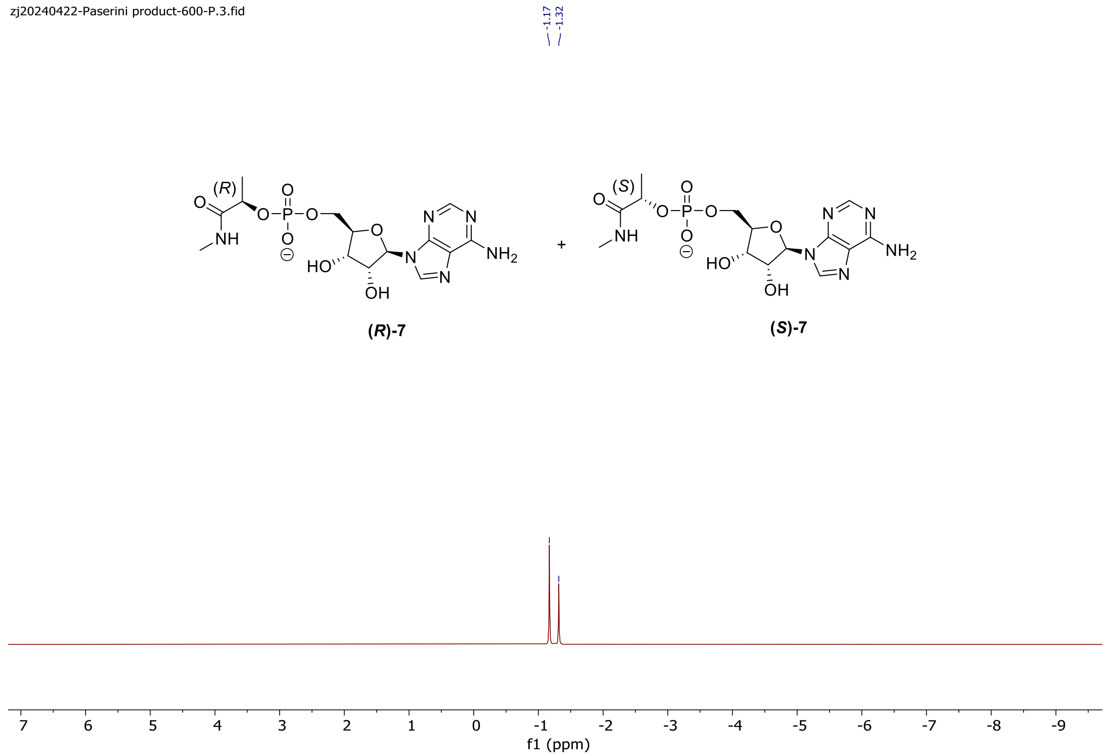

Figure S49. ^{31}P NMR of Passerini product 7 (243 MHz, D_2O). δ -1.17, -1.32.

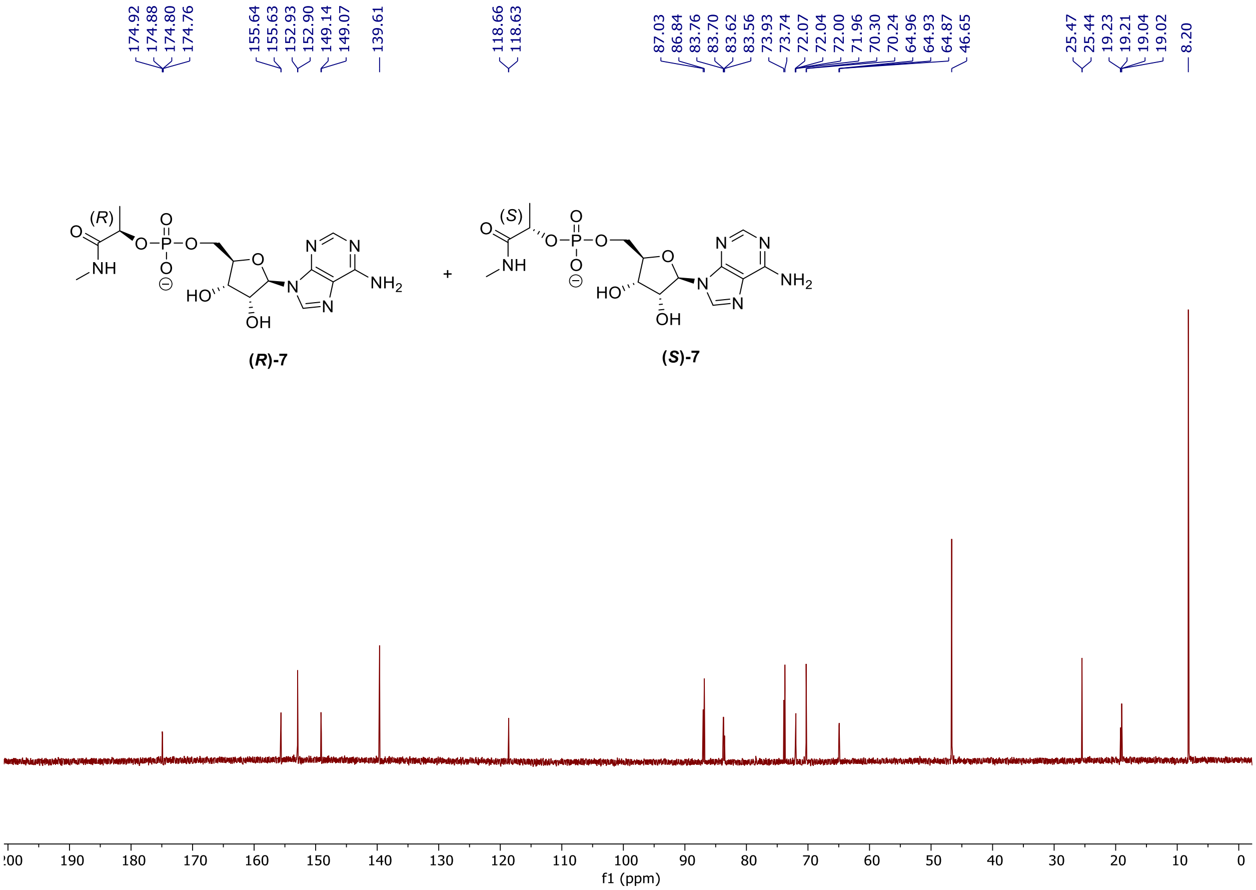

Figure S50. ^{13}C NMR of Passerini product 7 (150 MHz, D_2O).

[**major diastereomer**] δ 174.9 (d, *J* = 6.3 Hz), 155.64, 152.93, 149.14, 139.6, 118.63, 86.8, 83.7 (d, *J* = 8.1 Hz), 73.7, 71.97 (d, *J* = 5.4 Hz), 70.3, 64.94 (d, *J* = 5.7 Hz), 25.47, 19.0 (d, *J* = 3.3 Hz).

[**minor diastereomer**] δ 174.8 (d, *J* = 6.2 Hz), 155.63, 152.90, 149.07, 139.6, 118.66, 87.0, 83.6 (d, *J* = 8.9 Hz), 73.9, 72.05 (d, *J* = 5.4 Hz), 70.2, 64.89 (d, *J* = 5.4 Hz), 25.44, 19.2 (d, *J* = 3.3 Hz).

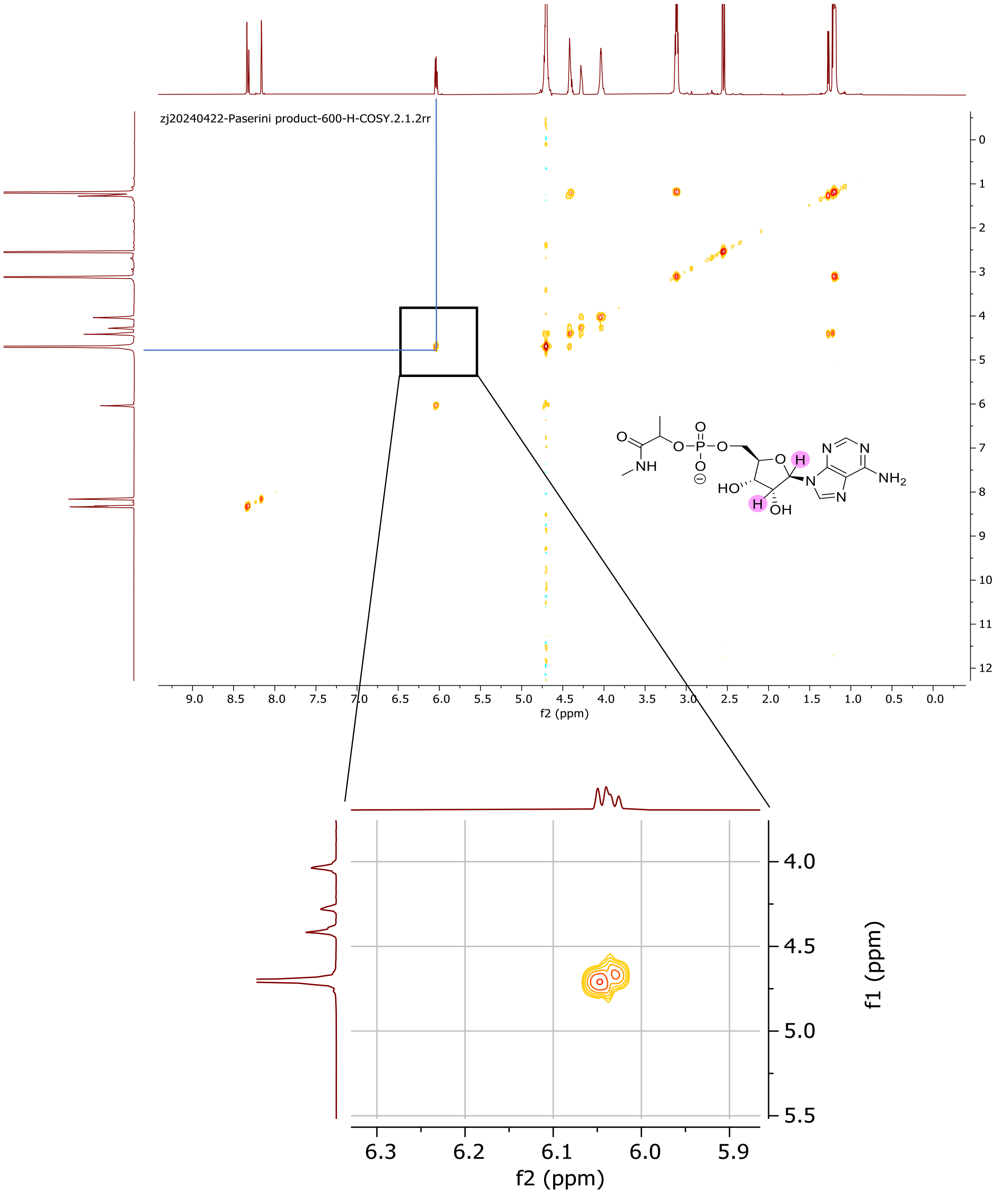

Figure S51. H-H COSY spectrum of Passerini product 7. The 2′H (coupling to 1′H) is masked by the peak of HOD.

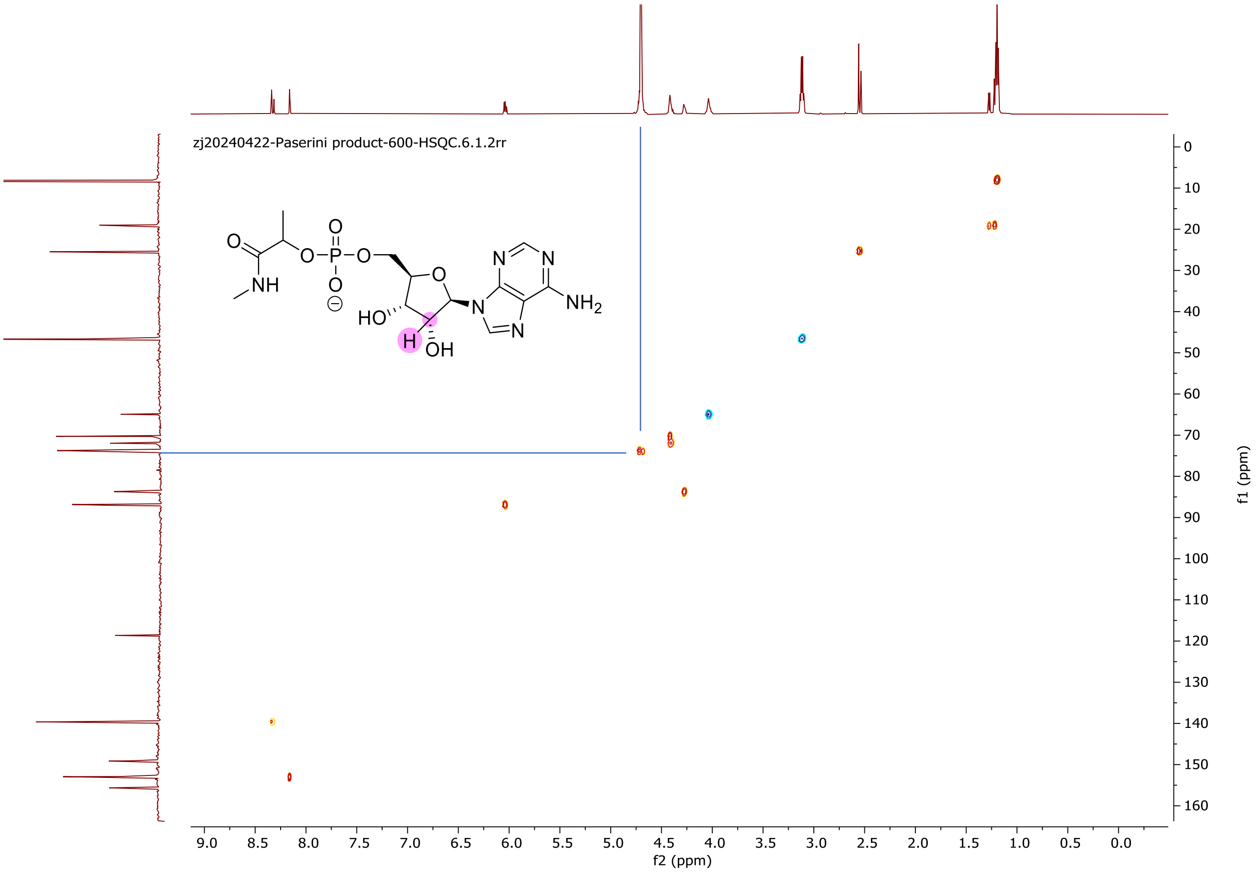

Figure S52. HSQC spectrum of Passerini product 7. The signal of 2′H is masked by HOD.

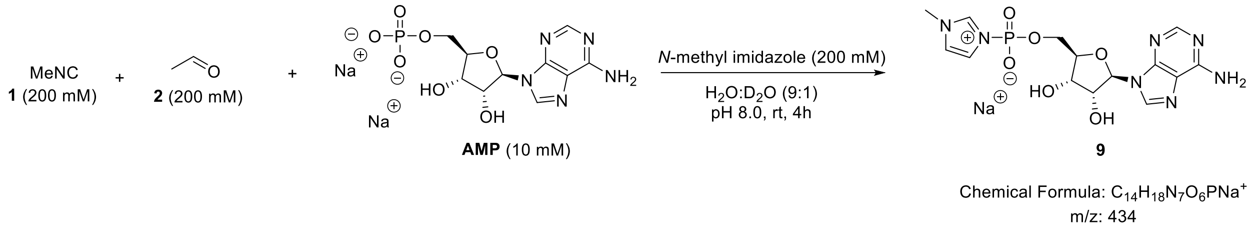

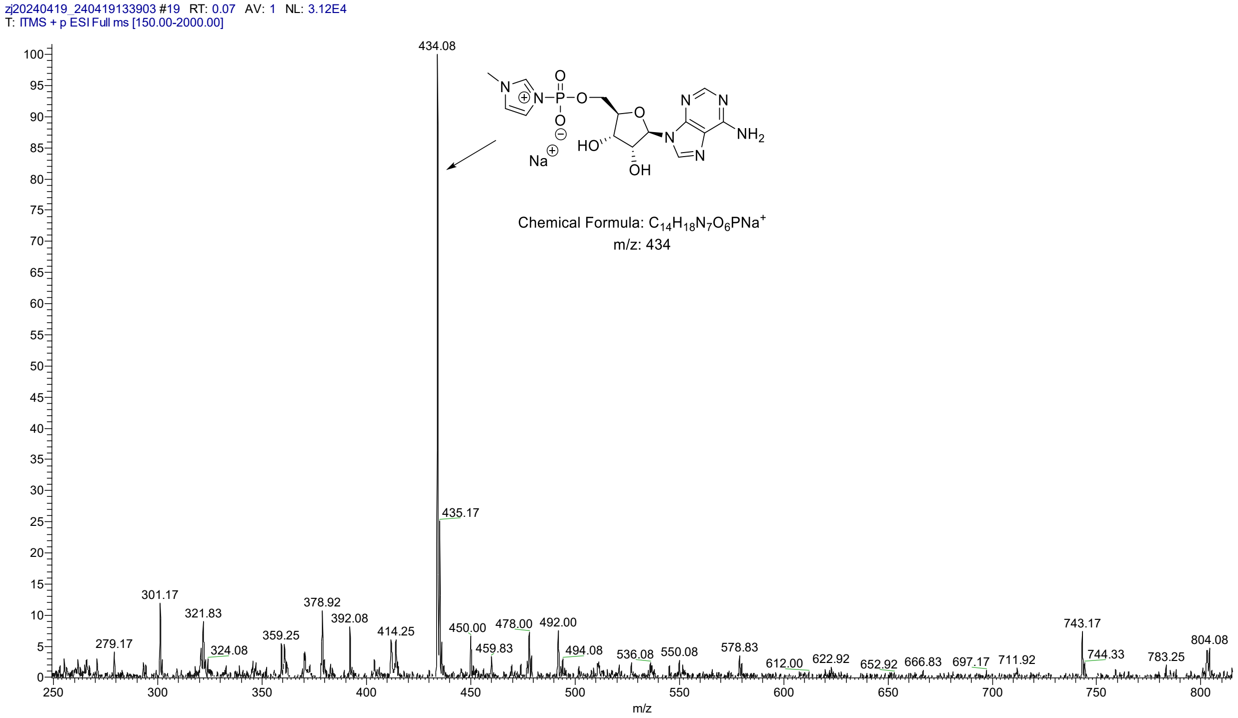

Figure S53. Mass spectrum of phosphorimidazolium 9 that derives from *N*-methylimidazole intercepted Passerini reaction.

Table S1. Sequences of oligoribonucleotides in this work.

oligo	Sequences	Resource
P1-amino	5′-NH_2-GUGUCGCUUUCG-3′	IDT
P1	5′-BODIPY-GUGUCGCUUUCG-3′	Prepared from P1-amino
P1-ddG	5′-Cy3-GUGUCGCUUUC$_{dd}$G-3′	In-house
L1	5′-p-AGCGAC-3′	In-house
Im-p-L1	5′-Im-p-AGCGAC-3′	In-house
L2	5′-AGCGAC-3′	IDT
P2-amino	5′-NH_2-GUGUCGCUCAGG-3′	IDT
P2	5′-BODIPY-GUGUCGCUCAGG-3′	Prepared from P2-amino
H1-3′o-amino	5′-NH_2-GUGUCGCUUUCG$_{3'p5'}$AGCGAC-3′	IDT
H1-2′o-amino	5′-NH_2-GUGUCGCUUUCG$_{2'p5'}$AGCGAC-3′	In house
H1-3′o	5′-BODIPY-GUGUCGCUUUCG$_{3'p5'}$AGCGAC-3′	Prepared from H1-3′o-amino
H1-2′o	5′-BODIPY-GUGUCGCUUUCG$_{2'p5'}$AGCGAC-3′	Prepared from H1-2′o-amino
T1	5′-GUCGCUCGAAAGCGACAC-3′	In-house
c-T1	5′-GUGUCGCUUUCGAGCGAC-3′	In-house
P3-amino	5′-NH_2-GAUGGUUCG-3′	In house
P3	5′-BODIPY-GAUGGUUCG-3′	Prepared from P3-amino
T2	5′- AAAAGUCGCUCGAACCAUCUGGAAAA-3′	In-house
c-T2	5′- UUUUCCAGAUGGUUCGAGCGACUUUU-3′	In-house
H2-3′o-animo	5′-NH_2-GAUGGUUCG$_{3'p5'}$AGCGAC-3′	In-house
H2-2′o-animo	5′-NH_2-GAUGGUUCG$_{2'p5'}$AGCGAC-3′	In house
H2-3′o	5′-BODIPY-GAUGGUUCG$_{3'p5'}$AGCGAC-3′	Prepared from H2-3′o-amino
H2-2′o	5′-BODIPY-GAUGGUUCG$_{2'p5'}$AGCGAC-3′	Prepared from H2-2′o-amino
$(A)_{10}$	5′-AAAAAAAAAA-Cy3-3′	In-house

$(U)_{10}$	5′-UUUUUUUUUU-Cy3-3′	In-house
$(C)_{10}$	5′-CCCCCCCCCC-Cy3-3′	In-house
$(GA)_5$	5′-GAGAGAGAGA-Cy3-3′	In-house
F1	5′-Cy3-GGACCUUCG-3′	In-house
F2	5′-p-GGUCCCGCAUCCCAGUCUUCG-3′	In-house
F3	5′-p-GACUGGUACAUGGCGUUAGGU-3′	In-house
dFx	5′- GGAUCGAAAGAUUUCCGCAUCCCCGAAAGGGUA CAUGGCGUUAGGU-3′	In-house
dFx-mut	5′-Cy3- GGACCUUCG GGUCCCGCAUCCCAGUCUUCG GACUGGUACAUGGCGUUAGGU-3′	In-house
8-mer substrate	5′-FAM-AGAAGCCA-3′	In-house

Note of Table S1:

1. All oligonucleotides were synthesized from the 3′ to 5′ end, except for P1-ddG, which was synthesized from the 5′ to 3′ end.
2. P1, P2, P3, H1 and H2 were prepared by labeling precursor RNAs that bear an amino group (P1-amino, P2-amino, P3-amino, H1-2′o-amino, H1-3′o-amino, H2-2′o-animo, H2-3′o-animo).
3. 5′-FAM (Fluorescein) labeling was done at the synthesis stage using 5′-Fluorescein phosphoramidite.
4. 5′-Cy3 labeling was done at the synthesis stage using Cyanine 3 phosphoramidite.
5. 3′-Cy3 labeling was done at the synthesis stage using Cyanine 3 CPG.
6. dFx-mut was purified first by PAGE and then by high-pressure liquid chromatography (HPLC) using a Nexera HPLC/UHPLC system (Shimadzu) with an Atlantis™ T3, 3 µm, 4.6 x 150 mm column. Detection was done at 260 nm using an SPD-M30A UHPLC Photodiode Array Detector.
7. 3′o refers to the 3′-5′ phosphodiester linkage between G and A shown as $G_{3'p5'}A$.
8. 2′o refers to the 2′-5′ phosphodiester linkage between G and A shown as $G_{2'p5'}A$.
9. Unless specified, all the oligomers are RNAs.

Table S2. Chemical structures of fluorescently labeled oligoribonucleotides.

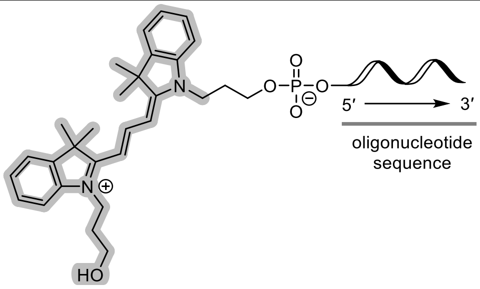 General structure of 5′-Cy3 labeled oligo
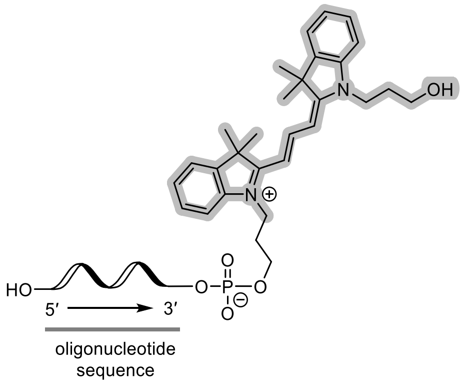 General structure of 3′-Cy3 labeled oligo
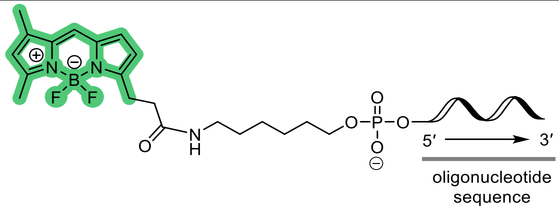 General structure of 5′ BODIPY labeled oligo
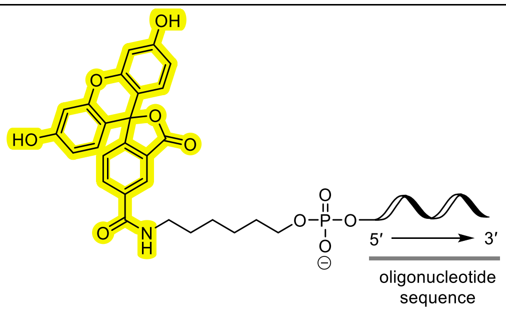 General structure of 5′-FAM labeled oligo

References

1. Schuster, R. E.; Scott, J. E.; Casanova Jr., J. Methyl Isocyanide. *Org. Synth.* **1966**, *46*, 75-75. DOI:10.15227/orgsyn.046.0075

2. Radakovic, A.; Wright, T. H.; Lelyveld, V. S.; Szostak, J. W. A Potential Role for Aminoacylation in Primordial RNA Copying Chemistry. *Biochemistry* **2021**, *60* (6), 477-488. DOI: 10.1021/acs.biochem.0c00943

3. Barszcz, B.; Hodorowicz, S. A.; Stadnicka, K.; Jabłońska-Wawrzycka, A. A Comparison of the Coordination Geometries of Some 4-Methylimidazole-5-carbaldehyde Complexes with Zn (II), Cd (II) and Co (II) Ions in the Solid State and Aqueous Solution. *Polyhedron* **2005**, *24* (5), 627-637. DOI: 10.1016/j.poly.2005.01.016

4. Vellé, A.; Cebollada, A.; Macías, R.; Iglesias, M.; Gil-Moles, M.; Sanz Miguel, P. J. From Imidazole toward Imidazolium Salts and *N*-Heterocyclic Carbene Ligands: Electronic and Geometrical Redistribution. *ACS Omega* **2017**, *2* (4), 1392-1399. DOI: 10.1021/acsomega.7b00138

5. Saegusa, T.; Taka-Ishi, N.; Fujii, H. Reaction of carbonyl compound with isocyanide. *Tetrahedron* **1968**, *24* (10), 3795-3798.
